# The Alternative Sigma Factor SigX Controls Bacteriocin Synthesis and Competence, the Two Quorum Sensing Regulated Traits in *Streptococcus mutans*

**DOI:** 10.1371/journal.pgen.1005353

**Published:** 2015-07-09

**Authors:** Michael Reck, Jürgen Tomasch, Irene Wagner-Döbler

**Affiliations:** Helmholtz-Centre for Infection Research, Department of Medical Microbiology, Group Microbial Communication, Braunschweig, Germany; Indiana University, UNITED STATES

## Abstract

Two small quorum sensing (QS) peptides regulate competence in *S*. *mutans* in a cell density dependent manner: XIP (sigX inducing peptide) and CSP (competence stimulating peptide). Depending on the environmental conditions isogenic *S*. *mutans* cells can split into a competent and non-competent subpopulation. The origin of this population heterogeneity has not been experimentally determined and it is unknown how the two QS systems are connected. We developed a toolbox of single and dual fluorescent reporter strains and systematically knocked out key genes of the competence signaling cascade in the reporter strain backgrounds. By following signal propagation on the single cell level we discovered that the master regulator of competence, the alternative sigma factor SigX, directly controls expression of the response regulator for bacteriocin synthesis ComE. Consequently, a SigX binding motif (cin-box) was identified in the promoter region of *comE*. Overexpressing the genetic components involved in competence development demonstrated that ComRS represents the origin of bimodality and determines the modality of the downstream regulators SigX and ComE. Moreover these analysis showed that there is no direct regulatory link between the two QS signaling cascades. Competence is induced through a hierarchical XIP signaling cascade, which has no regulatory input from the CSP cascade. CSP exclusively regulates bacteriocin synthesis. We suggest renaming it mutacin inducing peptide (MIP). Finally, using phosphomimetic *comE* mutants we show that unimodal bacteriocin production is controlled posttranslationally, thus solving the puzzling observation that in complex media competence is observed in a subpopulation only, while at the same time all cells produce bacteriocins. The control of both bacteriocin synthesis and competence through the alternative sigma-factor SigX suggests that *S*. *mutans* increases its genetic repertoire via QS controlled predation on neighboring species in its natural habitat.

## Author Summary

*Streptococcus mutans* is a bacterium of the human dental plaque that contributes to caries development. It controls two important survival mechanisms via a cell-density dependent communication system (quorum sensing): The synthesis of peptide antibiotics, and of a membrane apparatus for genetic competence, i.e. the ability to take up external DNA and integrate it into its own genome. *S*. *mutans* synthesizes two different signalling peptides to this end. It has remained elusive, how exactly these signals are propagated within the cell and why only a fraction of the population becomes competent. To actually observe under the microscope which bacterium in the population is activated, and which genes are required for the activation, we constructed strains of *S*. *mutans* that reported on the transcription of a gene by starting to fluoresce green. We even constructed strains that reported on two genes simultaneously, by fluorescing either green or blue or both. With these tools, and by additionally knocking out or modifying key genes as needed, we investigated the complete signaling cascade under various conditions. Thus we discovered a central regulatory switch. *S*. *mutans* makes sure that external DNA is available when it becomes genetically competent–by killing cells in the environment.

## Introduction

Horizontal gene transfer in prokaryotes is mediated via three distinct mechanisms comprising conjugation, transduction, and transformation [1,2]. Studying those mechanisms in detail is needed because they are among the reasons for the spread of antibiotic resistance and virulence determinants between bacteria [3]. Natural transformation, i.e. the uptake of extracellular DNA from the environment via genetic competence, is a powerful process able to expand and modify the gene inventory in both Proteobacteria and Firmicutes [4]. It requires a multi-protein complex localized in the cell membrane, many elements of which are highly conserved, and was studied in most detail in *Bacillus subtilis* and *Vibrio cholerae* [5,6].

In streptococci competence is a tightly controlled transient state whose activation involves detection of quorum sensing (QS) signaling peptides, which in streptococci are termed pheromones. In all streptococci studied so far the proximal master regulator of competence, and final receiver of the transduced signals, is the alternative sigma factor SigX (previously termed ComX). SigX binding to the RNA polymerase activates transcription of a core set of ~ 20 “late” competence effector genes [7]. They carry a nine bp cin-box in their promoter region and mediate the synthesis of proteins for DNA uptake and recombination [8]. The SigX regulon has been found in all streptococci sequenced to date, suggesting that genetic competence is ubiquitous in streptococci, although until now it could only be demonstrated experimentally in very few [9].

Although all streptococci use peptide pheromones for density dependent activation of *sigX* expression as well as bacteriocin synthesis, the details of signal transduction and integration differ widely between the different species. Two principal types of pheromones and signal detection mechanisms have been found [10]: Pheromones derived from pre-peptides carrying a double-glycine leader sequence are cleaved and exported by the membrane localized ComAB complex, and after an additional processing step the mature pheromone is detected in the extracellular environment through the transmembrane sensor histidine kinase of a two component signal transduction system that phosphorylates its corresponding response regulator. The phosphorylated response regulator induces transcription of the alternative sigma-factor *sigX*. The competence-stimulating peptide (CSP) of *S*. *pneumoniae* belongs into this group. The second type of pheromones was recently discovered and shown to be conserved in streptococci [7,11–13]. A pre-peptide is synthesized, exported and processed to yield a short hydrophobic mature peptide termed XIP (*sigX* inducing peptide), which is then re-imported by a permease. Its detection occurs by an intracellular transcription factor that interacts directly with the peptide signal [10]. The activated, dimeric regulator induces transcription of *sigX* by binding to its promoter region [12], much like the LuxR type QS regulators that are activated by binding of acylated homoserine lactons (AHLs), the QS signals of Proteobacteria.

In *S*. *mutans* competence is regulated via both types of QS signaling peptides and the medium composition determines which signal is active. Moreover the medium also determines whether competence is induced uni-modally [14] or only in a subpopulation of cells [7,15].

**Fig 1:**
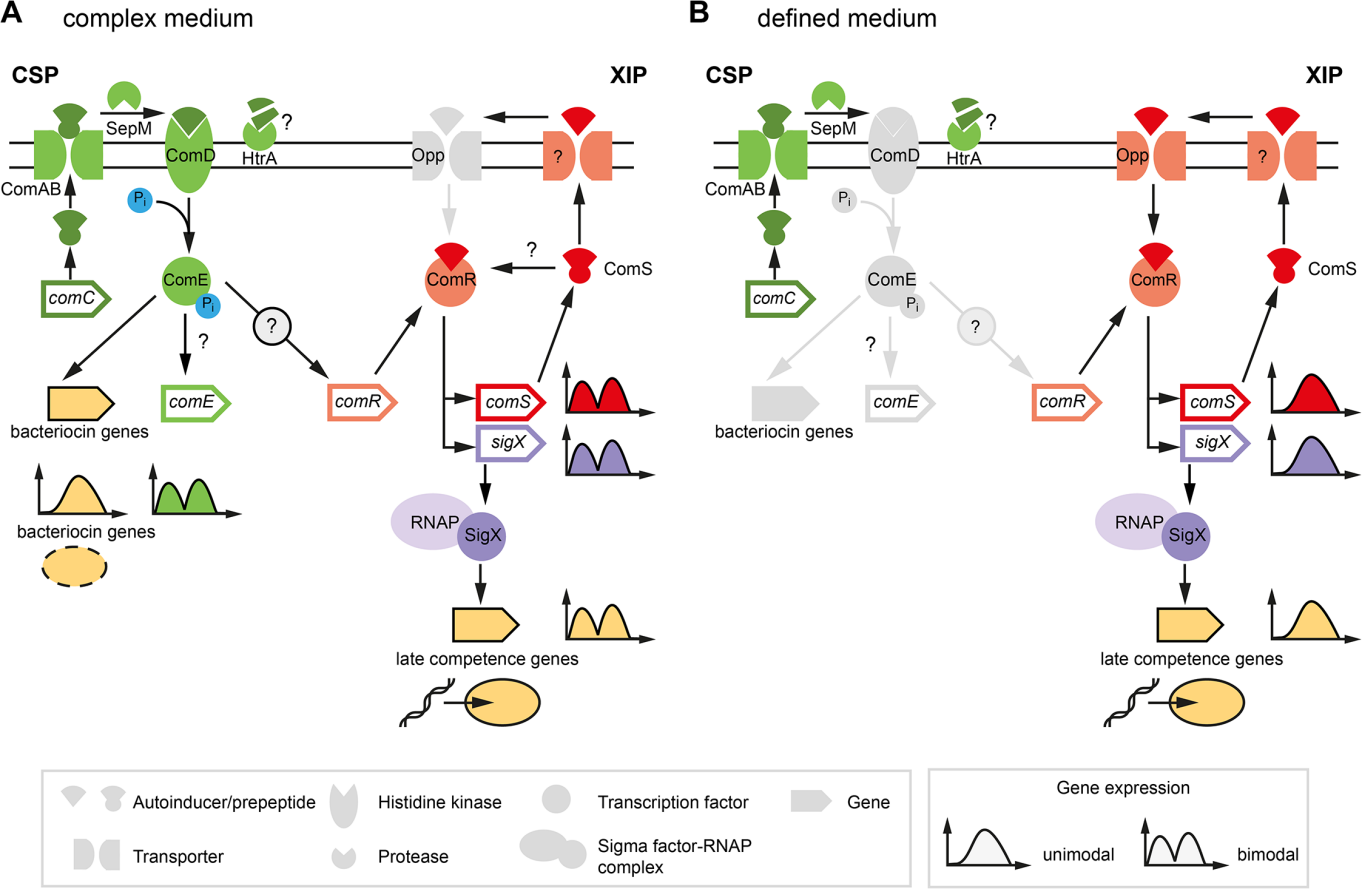
Previous model of competence development in *S*. *mutans* in complex (A) and defined medium (B). The different classes of structural components of the competence network are explained below in the legend (grey box in the left). Two quorum sensing pathways regulate competence in *S*. *mutans*. Components of the ComDE circuit with the autoinducer CSP are shown in green while components of the ComRS circuit and the autoinducer XIP are illustrated in red color. Inactive elements of the circiuts are shown in grey. The two QS pathways are operating in parallel and converge on the central comRS system. Both circuits are connected via an unknown link between ComDE and ComRS in complex medium. The medium determines which autoinducer is biologically active and whether competence is induced bimodally or unimodally across the population. This is illustrated by the gene expression shemes (grey box in the right). The modality of the expression of involved genes is illustrated in the same coloring as used for the gene symbol. The output of the systems, bacteriocin expression and competence, are symbolized below the bacteriocin encoding genes and the late competence genes in yellow. Bacteriocin expression is exclusively observed in complex medium while competence development in defined medium is not coupled to bacteriocin expression.

The current understanding of competence development in *S*. *mutans* is shown in Fig 1. In complex media competence can only be induced by CSP (Fig 1A). The 46 amino acid CSP precursor is encoded by the *comC* gene and processed and exported by the *comAB* encoded ABC transporter, yielding the extracellular 21 residue CSP-peptide [16]. This is cleaved to its biologically active form by the protease SepM, thereby removing the 3 C-terminal amino acids [17]. Binding of CSP-18 to the histidine kinase ComD induces autophosphorylation of the protein and results in the transfer of the phosphorylgroup to a conserved aspartic acid residue of the cognate response regulator ComE. Phosphorylated ComE binds to two direct repeats in the promoter regions of genes encoding bacteriocins and their corresponding immunity proteins resulting in transcription of the “early competence genes” [18–20]. Via an unknown link *comDE* activation induces *comRS* expression and finally competence development in a subpopulation of the cells [7,15].

In the peptide free chemically definde medium (CDM), competence can only be induced by XIP, the second autoinducer of *S*. *mutans* (Fig 1B), via the ComRS system, that is also present in other mutans, pyogenic and bovis group streptococci [7,21]. It consists of an Rgg type transcriptional regulator (ComR) and a small XIP peptide, encoded by the *comS* gene [7]. The XIP pre-peptide is processed and the active seven residue pheromone is secreted into the environment by an unknown process. After accumulation extracellular XIP is internalized, most likely via the Opp permease, and binds to ComR in a proposed stoichiometry of 2:1 (XIP:ComR) [7,12]. Resulting conformational changes promote dimerization of the XIP2/ComR complex and binding to DNA targets containing the ComR binding motif. In *S*. *mutans* binding motifs for ComR were found upstream of the *comS* and the *sigX* gene [7,12]. Thus XIP controls its own expression in a positive auto-regulatory feedback loop. XIP mediated competence induction is exclusively observed in chemically defined media and results in uni-modal expression of competence.

The effect of the medium is hypothesized to be caused by differences in transport and degradation, respectively, of the autoinducers. In *S*. *pneumoniae* it was shown that CSP is degraded by the membrane bound protease HtrA, and that this degradation is reduced in the presence of unfolded proteins [22]. Therefore, it was hypothesized that the homologous HtrA of *S*. *mutans* might be inhibited from CSP degradation by small peptides present in complex medium, resulting in active CSP in complex but not in minimal medium [15]. Conversely, XIP is active in defined, but not in complex media. It was suggested that the import of XIP might be inhibited in complex media due to the clogging of the Opp permease [15]. Small amounts of peptides that are added to the cultivation medium completely eliminate the activity of XIP [15]. However, it remains unclear how under CSP induced conditions ComR might be activated by XIP, if this signal cannot be imported into the cell (Fig 1A).

Bacteria often respond to environmental stimuli in a non-uniform manner; even in isogenic cultures and under homogenous conditions the appearance of multiple phenotypes (phenotypic variation) is observed [23]. Phenotypic variability has strong implications for the treatment of bacterial infections and is relevant for cellular differentiation [24]. Two distinct phenotypes occurring simultaneously are referred to as bimodal and were observed e.g. for lactose utilization, chemotaxis and persister cell occurence in *E*.*coli* [25] and for competence development and sporulation in *B*. *subtilis* [23,26,27]. It was previously shown that phenotypic heterogeneity enhances the overall fitness of the population under fluctuating conditions and helps bacteria to colonize different ecological niches within an ecosystem [23,28]. Thus phenotypic variability can be considered as a bet-hedging strategy and an evolvable trait.

Signaling in bacteria is never discrete since stochastic fluctuations of the components that determine a cellular state occur [29]. This phenomenon is called noise and it is most pronounced for processes involving a limited number of molecules such as transcription and translation [30]. Noise is one critical determinant for the establishment of phenotypic variation [23,29]. Phenotypic heterogeneity can also be a result of the architecture of regulatory networks comprising positive feedback loops or toggle switches (two regulators that negatively regulate each other) which can amplify signals and respond nonlinearly to stimuli [23,24]. Noisy gene expression in conjunction with such networks can convert a graded signal into a bimodal response which is stably maintained in the population.

An intriguing feature of the competence cascade in *S*. *mutans* is its bimodality under CSP induced conditions in THBY medium. It was hypothesized to be caused by the auto-regulatory feedbackloop of ComS [15]. A different hypothesis was developed by Lemme *et al*. [14] who found that the entire population expressed the bacteriocin related genes in an uni-modal way, while only a subpopulation expressed *sigX* and entered the competent state. However, bimodal expression of *comE* was observed at the same time. Since *comE* is supposed to act upstream of ComRS signaling, it was therefore hypothesized that *comE* expression might be the origin of bimodality [14].

Finally, an intriguing question is the autolysis of a fraction of the population after stimulation by XIP [31] or CSP [14,32,33]. In *S*. *pneumoniae*, it is the non-competent subpopulation which is killed by the competent siblings, a phenomenon termed fratricide [9]. The CbpD murein hydrolase which is responsible for this process has a homologue termed LytFsm in *S*. *mutans*. It has however been shown that lysis occurs in a fraction of the competent cells [14,34] and that LytFsm is a self-acting autolysin, not a fratricin [32].

The QS signaling cascade of *S*. *mutans* thus comprises two different signals, one that is detected in the external environment (CSP), and one that is detected intracellularly (XIP). They are linked with each other in different ways in different media. The system has a temporal dimension (“early” and “late” competence genes), and it can result in phenotypic heterogeneity of the population. Here we asked (1) At which level in the competence signaling cascade is bimodal gene expression triggered? (2) How is the ComE signaling cascade connected to the ComRS signaling cascade? (3) How can bacteriocin expression be unimodal, although *comE* expression is bimodal? (4) How is the medium effect on signaling mediated—is CSP really degraded in CDM, and how can an active XIP/ComR complex be formed in THBY?

To answer these questions, it was necessary to follow the activation of the key genes along the signaling cascade on the single cell level. We developed a tool-box of integrative reporter plasmids carrying a fusion of the promoter of interest with a fluorescent protein. Those constructs were integrated into the chromosome of *S*. *mutans*. We used the wild-type, as well as strains that had key genes of the signaling cascade deleted, over-expressed, or modified. To monitor co-expression of “early” and “late” competence genes, we developed dual-fluorescent reporter strains that clearly demonstrated which of the cells induced upstream became finally competent. Population heterogeneity was observed directly under the microscope or using flow cytometry. RNA sequencing was used to determine gene expression of all involved components during the first 30 min post induction. The data result in a new understanding of the QS regulatory circuit of *S*. *mutans* that resolves the discordant observations described above.

## Results

### Signal propagation through the competence cascade

**Fig 2:**
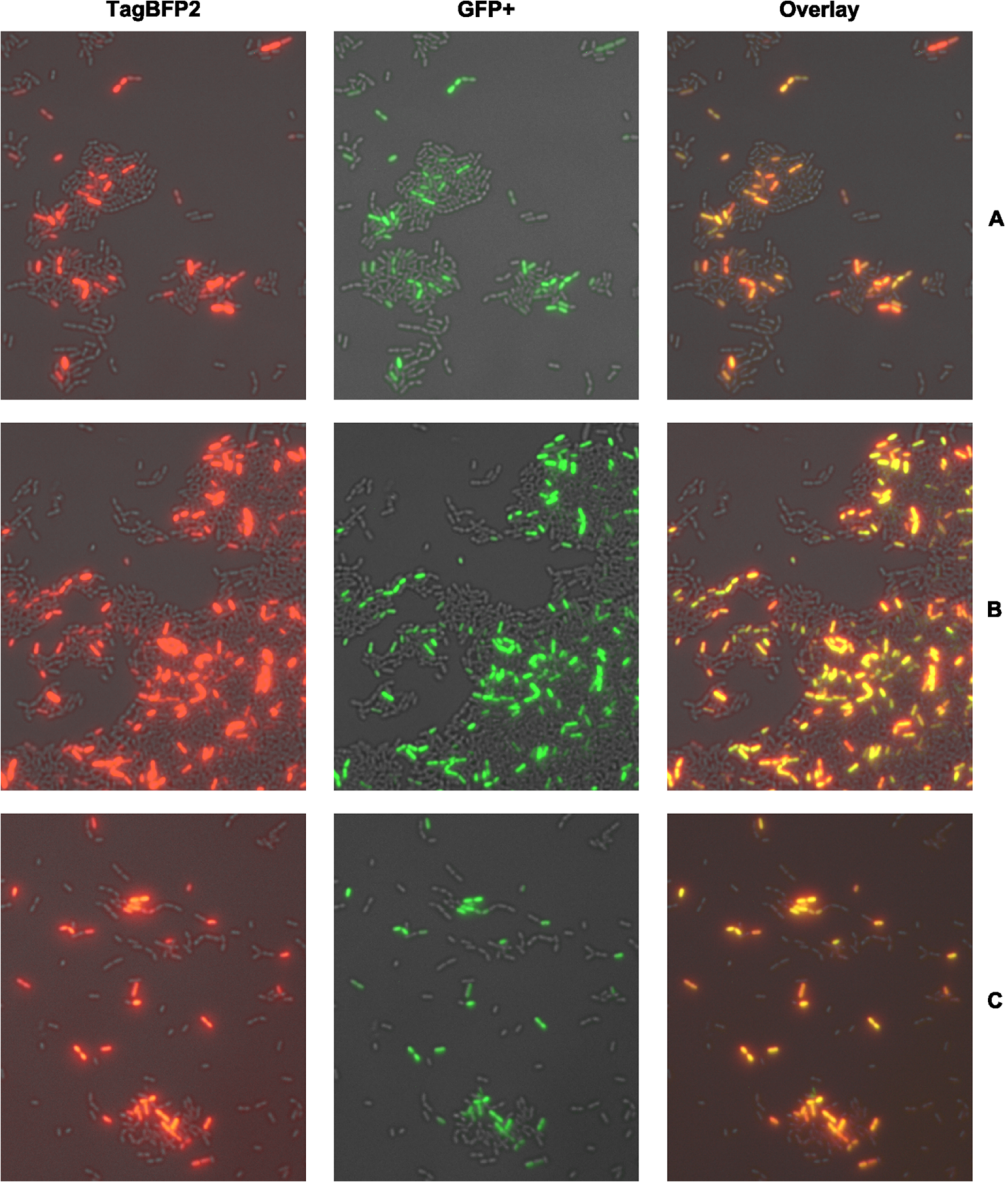
Single cell co-expression analysis of *comE* with the late competence genes *lytFsm* (A), *comS* (B) and *sigX* (C) using dual fluorescent reporter strains. Strains were grown in complex THBY medium and gene expression induced with 2 μM CSP. 150 minutes post induction cells were collected and imaged using fluorescence microscopy. *ComE* expressing cells show green fluorescence (middle column), while the blue fluorescence (left column) indicates expression of co-expression partners. An overlay of the green and blue channel is shown in the right column. For better visualization the blue fluorescence is shown in red false color.

The construction and experimental verification of all single and dual fluorescent reporter strains used in this study is described in detail in S1 Text and S1–S6 and S9 Figs. Using dual fluorescent reporter strains we analyzed signal propagation along the entire CSP-induced competence cascade in *S*. *mutans*, from the upstream *comDE* system to the central regulatory *comRS* module and the alternative sigma-factor *sigX* to the late competence gene *lytFsm*, a component of the transformasome. First the effect of the CSP inducer concentration was evaluated. S7 Fig (flow cytometry) and S8 Fig (microscopy) show that bimodal *lytFsm* expression was observed above a concentration of 20 nM CSP; the percentage of cells expressing *lytFsm* was not significantly changed between 200 nM and 100 μM CSP. Thus 2 μM CSP was chosen as inducer concentration in all following experiments. No significant influence on cell growth and viability was observed under these conditions while higher concentrations of CSP induce cell death and growth arrest [34]. The analysis was conducted 2.5 h post induction because the expression of the late competence genes reaches its maximum after 2 h [14] and the full maturation of the fluorescent proteins requires additional 30 minutes [35,36]. Fig 2 shows that the *comE* gene is co-expressed with *comS*, *sigX* and the late competence gene *lytFsm*. Moreover and not surprisingly *comS* is coxpressed with *sigX* (S5B Fig), since both genes share the same ComR binding sequence in their 5`UTR [7].,. All four genes are expressed in a bimodal way. The same coexpression patterns were also observed when we switched fluorophores for the two genes under study in the reporter strains (S9 Fig). To conclude, we observed an identical bimodal gene expression pattern for all genes in the signaling cascade. Two mechanistic explanations would be in accordance with the observed coexpression pattern and the bimodality of *comE* expression. Both models assume a basal expression of *comE*.

1. ComE itself might represent the origin of bimodality and propagates the signal linearly to the downstream genes.
2. One of the downstream regulatory systems (e.g. ComRS or SigX) induces expression of the upstream localized *comE* gene in a subpopulation, leading to bimodality.

### Overexpression of ComDE and ComRS

**Fig 3:**
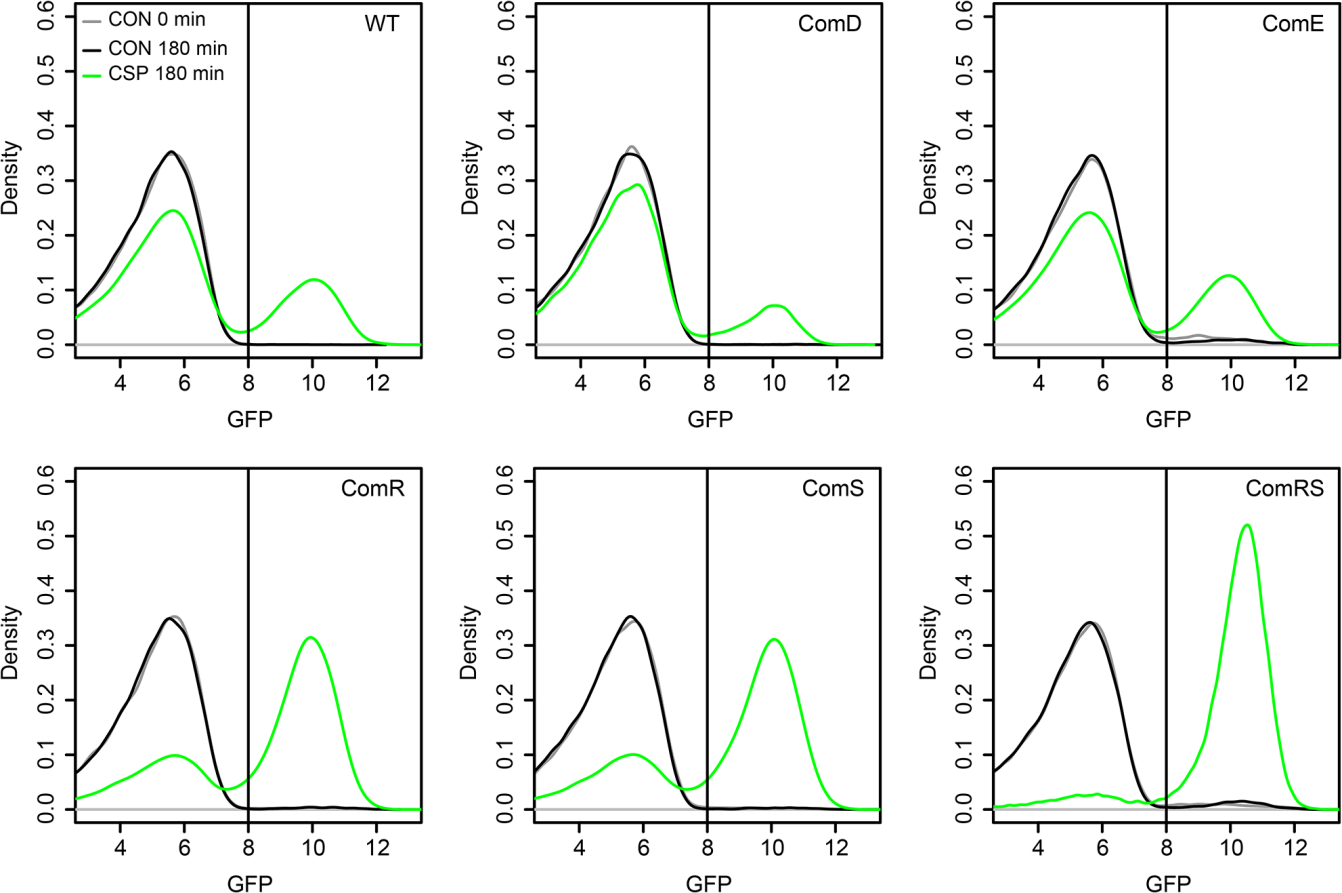
Overexpression analysis of *comE*, *comR*, *comS* and *comRS* in the LytFsm reporter strain background. Expression of the different genes was under the control of the strong constitutive lactococcal P23 promoter on the replicative plasmid pIB166. Strains were grown in THBY under CSP induced (2μM) and uninduced conditions. 180 min post induction samples were collected and analyzed using a flow cytometer. A sample collected directly before induction (0 min) was used as negative control. The distribution of the gfp fluorescence intensity of 50.000 analysed cells as determined by flow cytometry is shown in the density plots. Density plots derived from uninduced cells at t = 180 min are shown in black while the density plots of CSP induced cells at that timepoint are shown in green. Density plots of the control at t = 0 min are shown in grey.

To determine whether ComD/ComE or ComR/ComS represent the origin of bimodality during the CSP-induced competence development of *S*. *mutans*, we constructed overexpression strains strongly and constitutively expressing those proteins independent from their native promoters and thus equally across the entire population. Overexpression of the cellular component(s) representing the origin of bimodality should cause unimodal *lytFsm* expression (indicative of unimodal competence) across the population. We utilized plasmid pIB166 [37] and cloned the genes downstream of the strong constitutive Lactococcal P23 promoter. Plasmids were transformed into the LytFsm pAE03 reporter strain. *LytFsm* is a late competence gene [32] and it is the most strongly expressed late competence gene of the tested reporters (S4 Fig). We used the *comRS* overexpression strain in defined medium to test whether unimodal *lytFsm* expression is observed, as expected according to Masburn-Warren et al.[7]. *LytFsm* expression was unimodally induced even at low cell densities and without addition of external XIP (S10 Fig), indicating that endogeneous production of XIP is sufficient to induce competence unimodally. To analyse the origin of bimodality, the experiments were conducted in THBY under CSP inducing conditions. Fig 3 shows that for all overexpression strains CSP was needed to induce *lytFsm* expression. In a transcriptional signaling cascade from *comDE* to *sigX* it would have been expected that overexpression of one component should be sufficient to induce competence. The strong constitutive overexpression of *comE* or *comD* in the entire population did not enhance the percentage of *lytFsm* induced cells (Figs 3 and S11). Thus we can exclude that ComE or ComD is the origin of bimodality. Overexpression of only *comR* or only *comS* still resulted in a biphasic population behavior, although the proportion of *LytFsm* expressing cells was significantly enhanced compared to the WT or the *comE* overexpression strain. However, overexpression of both genes together (*comRS*) from the lactococcal P23 promoter resulted in unimodal expression of *LytFsm*, proving that *comRS* represents the origin of bimodality (Figs 3 and S11). ComR needs to be activated by XIP which is synthesized by *comS* [12]. Thus overexpression of *comR* or *comS* alone is not sufficient to induce *sigX* and the late competence genes. Both *comR* and *comS* are needed for the positive feedback loop to occur.

Surprisingly, however, unimodal competence development in the *comRS* overexpression strain absolutely required CSP induction in THBY medium. No competence development was observed in the absence of CSP. A regulator upstream of *comRS* as assumed currently (Fig 1B) cannot be responsible for this observation, because overexpression of *comRS* would be able to overcome it. In complex medium it is thought that import of secreted XIP is impossible due to the block of the Opp peremease by small peptides (Fig 1A). The data suggest that CSP itself or induced cellular changes are able to bypass this block.

### Combined effect of XIP and CSP on *lytFsm* expression

**Fig 4:**
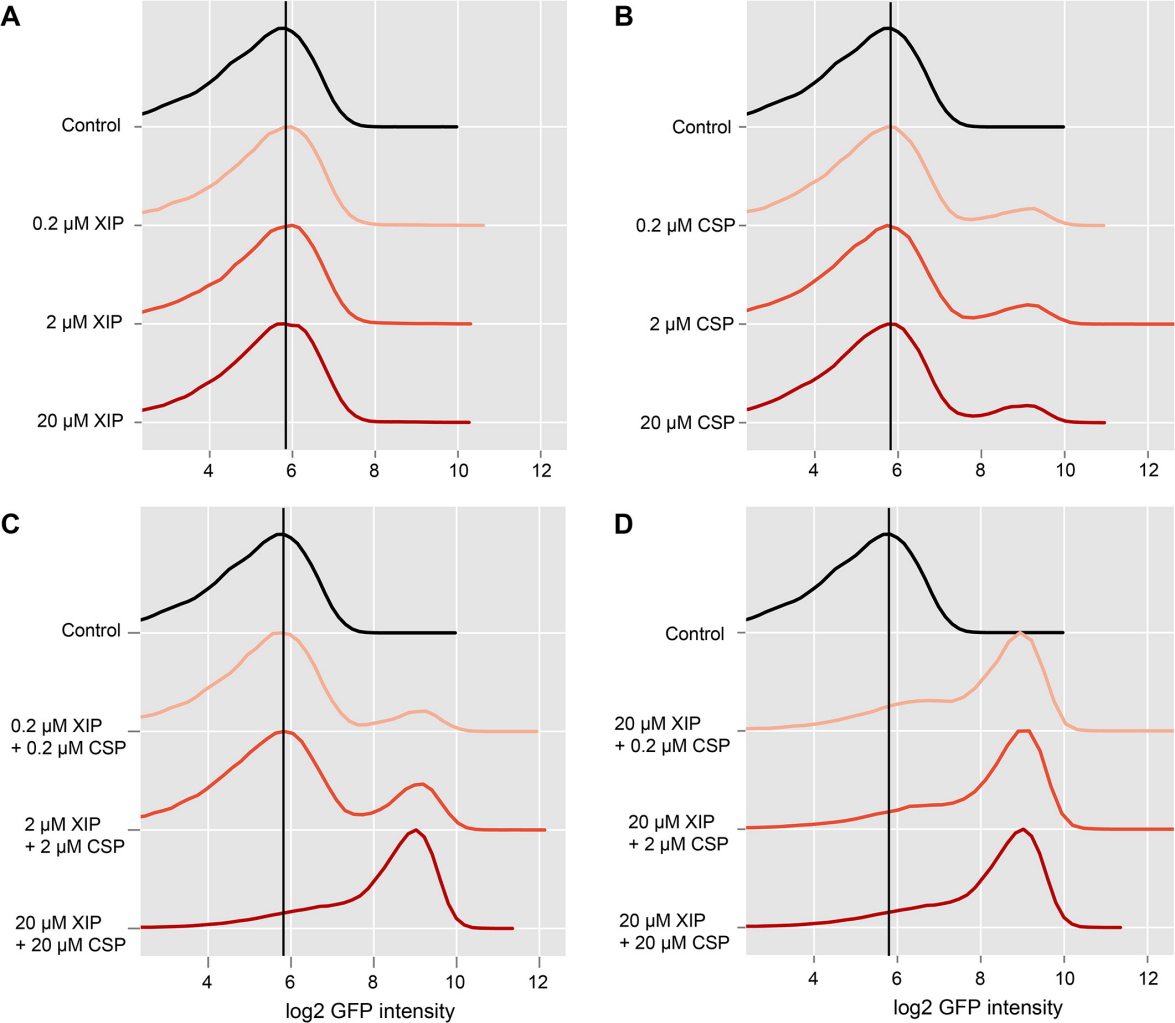
Combined effect of CSP and XIP peptides on lytFsm expression in complex medium. The LytFsm pAE03 reporter strain was grown in complex medium until the culture reached an OD_600_ of 0.2. The culture was divided and induced with either CSP or XIP alone or treated with combinations of XIP and CSP. Concentrations between 200 nM and 20 μM of each inducer were tested. 2 h post induction cells were harvested and analyzed using flow cytometry. 50.000 cells were analyzed for each condition. Density plots of the log2 gfp fluorescence intensity for the differently treated reporter cells are shown. Controls are shown in black. Increasing inducer concentrations are visualized by increased intensity of the red color. The vertical black line represents the peak fluorescence intensity of the uninduced control. **A** Effect of increasing concentrations of XIP on the fluorescence of the LytFsm reporter strain. **B** Effect of increasing concentrations of CSP on the reporter strain. **C** LytSsm fluorescenc of cells treated with combinations of XIP and CSP in equimolar ratios. **D** Effect of different molar ratios of XIP to CSP.

Thus we tested how the percentage of cells expressing the late competence gene *lytFsm* was influenced by XIP supplementation to a CSP induced LytFsm reporter strain grown in complex medium and how the relative amounts of those two signals affect bimodal gene expression. Accordingly different combinations of CSP and XIP with concentrations ranging from 0.2 μM to 20 μM of each inducer were tested. The results of the flow cytometric analysis of the LytFsm pAE03 reporter strain 120 minutes post XIP and/or CSP supplement are presented in Fig 4. As expected, XIP supplementation alone did not induce expression of the late competence gene *lytFsm*, regardless of the used concentration (Fig 4A). Conversely, and as expected, too, addition of CSP alone induced bimodal *lytFsm* expression. The percentage of cells expressing *lytFsm* was always constant, independent of the used CSP concentration in a range from 0.2 μM to 100 μM (Figs 4B and S7 and microscopic images in S8 Fig). However, addition of both XIP and CSP in a ratio of 1:1 resulted in expression of *lytFsm* (Fig 4C) while no expression had been observed with XIP alone up to a concentration of 20 μM. At the highest concentration of XIP and CSP tested here, unimodal expression of competence was observed. Lowering the CSP concentration to 0.2 μM still resulted in unimodal *lytFsm* expression (Fig 4D). The XIP concentration strongly determines the percentage of *lytFsm* expressing cells, while the CSP concentration is of minor importance.

We hypothesize that extracellular XIP is imported into the cell in a concentration dependent way, but only in the presence of CSP. How can this be achieved? The XIP uptake must be independent of Opp, since its deletion does not affect the CSP induced bimodal competence phenotype in complex medium [15]. We suggest that CSP induces import of XIP in an indirect way by inducing expression of bacteriocins, resulting in permeabilization of the cell. As relatively high XIP concentrations are required, it is likely that the import of the small XIP peptide is ineffective in THBY. To further substantiate this hypothesis we deleted the *cipB* gene in the LytFsm reporter strain background and tested whether addition of CSP or XIP or the combination of both petides still induces *lytFsm* expression. Strinkingly no induction of *lytFsm* expression was found in the CipB deletion strain, regardless which peptide or peptide combination was used for induction (S12 Fig). Accordingly, Perry et al.[34] observed that CipB deletion completely abolished CSP induced competence development.

Since there is no direct regulatory link between *comDE* and *comRS* (see above), and since competence induction by CSP in complex medium appears to be an indirect effect, we hypothesize that CSP controls only bacteriocin synthesis, while competence is exclusively controlled via XIP.

### Biological activity of CSP in chemically defined medium

Previously only competence development has been tested in both CDM and THBYmedia, and it was shown that in CDM competence is not induced by CSP, regardless of the used concentration [15]. It was speculated that CSP might be proteolytically degraded by the HtrA protease in CDM, while in complex medium this protease was thought to be saturated by small peptides that are constituents of the medium, thus protecting CSP from degradation [15]. However, it was not yet tested if bacteriocin encoding genes are transcribed in CDM under CSP induced conditions. Using the CipB pMR1 reporter strains in the different deletion backgrounds we therefore tested whether CSP induction in CDM promotes transcriptional activation of bacteriocins. We show (Table 1 and S13 Fig) that CSP promotes a strong induction of *cipB* expression in CDM and thus is biologically active and not degraded. Induction of *cipB* expression occurs via ComDE, as deletion of either *comD* or *comE* completely abolished it. The induction of *cipB* expression was unchanged in the Δ*comRS*, Δ*comS* and Δ*sigX* background. An influence of those regulatory systems can thus be excluded. To summarize, CSP is active in CDM. It induces transcription of bacteriocin encoding genes via ComDE but does not induce competence via the ComRS system.

**Table 1:** Expression of *cipB* in different gene deletion background in CDM under CSP induced conditions. In the genetic background of the fluorescent reporter strain CipB pMR1 deletion mutants for Δ*comC*, Δ*comD*, Δ*comE*, Δ*comS* and Δ*comRS* were constructed, cultivated in CDM medium and induced by CSP (2 μM). Fluorescence microscopic images were obtained 3 hours after induction. Uninduced strains were used as controls. Tickmarks indicate detected fluorescence, tickmarks in brackets indicate weak fluorescence, a minus indicates no detectable fluorescence.

CDM			
Reporter genotype	2 μM CSP	Control	Strain
CipB	**✓**	**-**	CipB pMR1
CipB ∆comC	**✓**	**-**	CipB pMR1ΔcomC
CipB ∆comD	**-**	**-**	CipB pMR1ΔcomD
CipB ∆comE	**-**	**-**	CipB pMR1ΔcomE
CipB ∆comS	**✓**	**-**	CipB pMR1ΔcomS
CipB ∆comRS	**✓**	**-**	CipB pMR1ΔcomRS
CipB ∆SigX	**✓**	**-**	CipB pMR1ΔcomX

### Transcriptome analysis of CSP and XIP signaling in CDM

To demonstrate that XIP signaling induces exclusively competence, while CSP controls only bacteriocin expression, we performed a time resolved analysis of the transcriptional response to these two autoinducers during the first 30 min after supplementation using RNA sequencing.

**Fig 5:**
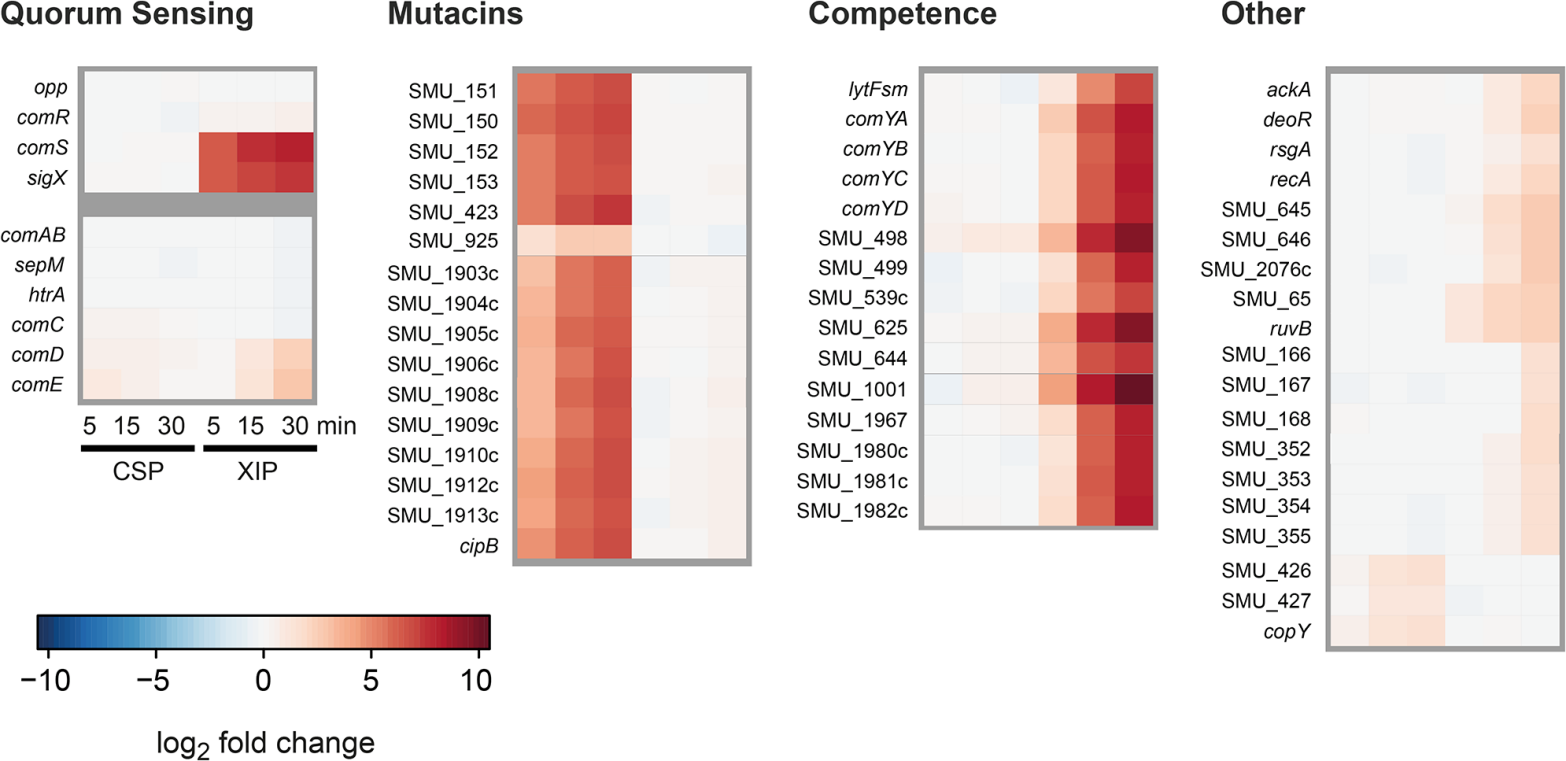
Time resolved transcriptome analysis of XIP and CSP induced *S. mutans* WT cultures in CDM. *S*. *mutans* WT cells were grown in CDM and induced either with 2 μM CSP or 2 μM XIP. Samples were taken 0, 5, 15 and 30 minutes post induction. Samples from uninduced controls were taken at corresponding timepoints. Differentially expressed genes (log2 fold > 1) were clustered into the categories competence, mutacins, quorum sensing and others. Differential expression of the respective genes is displayed in a heatmap. Genes that belong to the two quorum sensing regulons controlling competence in *S*. *mutans* but were no differentially expressed are also shown.

Differentially expressed genes are presented in Fig 5 and are grouped according to quorum sensing related genes, mutacins, competence and others. CSP supplemention in CDM induced the expression of mutacins and their corresponding immunity proteins, while competence related genes were not found to be differentially expressed. The exact opposite behavior was observed for XIP stimulation in CDM, confirming our hypothesis. For the XIP signaling cascade it can be seen that the transcription of the central XIP responsive transcriptional regulator ComR was only very slightly induced, in accordance with the general observation that essential key regulators are unlikely to be strongly transcriptionally regulated. The primary targets of ComR, c*omS* and *sigX* were instantly induced by XIP, representing the strongest responding transcripts. The up-regulation of the two genes already 5 minutes after XIP addition is in accordance with the post-translational activation of the ComR protein by XIP binding. Interestingly, XIP addition resulted in the activation of *comDE* expression for the later time-points (15 and 30 min). The CSP signaling cascade, by contrast, did not induce *sigX* or *comS* expression as expected. *comDE* expression was only very slightly induced upon CSP stimulation. However, mutacins, the primary targets of ComE, were strongly transcribed already 5 min post supplementation, suggesting post-transcriptional activation of the regulator ComE.

Genes encoding transporters and proteases (ComAB, HtrA, Opp and SepM) as well as *comC*, encoding the CSP precursor, were not differentially expressed, regardless if XIP or CSP was used for stimulation.

Both QS systems responded very fast, within the first 5 minutes, to their stimuli. Thus the results of the transcriptome analysis indicate that QS in *S*. *mutans* is initiated by the regulators ComE and ComR on the post-transcriptional level. The transcription of the target genes mutacins, *comRS* and *sigX* can already be observed 5–15 minutes after stimulation. The response of the “late competence” genes is slightly delayed compared to that of the mutacins, since the activation of SigX is additionally required. The expression of *comE* and *cipB* was confirmed by quantitative PCR (S1 Text and S15 Fig).

To summarize our findings up to this point: The data show that *comRS* is the origin of bimodality. There is no direct signaling cascade from CSP to ComRS. CSP induces bacteriocins. Its role in competence stimulation in complex medium is an indirect one, most likely as a result of bacteriocin synthesis causing permeabilization of the cells and reimport of endogenously produced and secreted XIP.

The co-expression analysis described above however raised the question, why *comE* is coexpressed with *comS*, *sigX* and the late competence gene *lytFsm*. We hypothesize that transcription of *comE* is controlled by ComR or SigX.

### XIP controls *comE* expression via SigX

If SigX or ComR would control *comE* transcription, then the XIP signal should induce *comE* expression independent of ComDE. We tested this hypothesis using the *comE* reporter strain in different gene deletion backgrounds under XIP induced conditions in CDM. Table 2 (and microscopic pictures in S16 Fig) shows that this is indeed the case. The transcriptional activation of *comE* is independent of internal production and external sensing of CSP as it also occurs in ∆*comDE* and ∆*comC* deletion strains. Deletion of *comRS* and surprisingly also *sigX* completely abolished transcriptional activation of *comE*. In the Δ*comS* background, transcription of *comE* is slightly reduced, since the internal production of XIP is missing and cannot be completely restored by externally added XIP. In THBY, *comE* transcription was similarly dependend on ComRS and SigX (S1 Text and S5 Table). These data provide strong evidence for a direct regulatory role of SigX for the expression of *comE*. Since the targets of SigX contain a cin box in their 5`UTR [38], we analysed the promoter region of *comE*.

**Table 2:** Expression of *comE* in different gene deletion background in CDM under XIP induced conditions. In the genetic background of the fluorescent reporter strain ComE pMR1 deletion mutants for Δ*comC*, Δ*comD*, Δ*comE*, Δ*comS* and Δ*comRS* were constructed, cultivated in CDM medium and induced by XIP (2 μM). Further explanations as in Table 1.

CDM			
Reporter genotype	2 μM XIP	Control	Strain
comE	**✓**	**-**	ComE pMR1
comE ∆comC	**✓**	**-**	ComE pMR1ΔcomC
comE ∆comD	**✓**	**-**	ComE pMR1ΔcomD
comE ∆comE	**✓**	**(✓)**	ComE pMR1ΔcomE
comE ∆comS	**(✓)**	**-**	ComE pMR1ΔcomS
comE ∆comRS	**-**	**-**	ComEpMR1ΔcomRS
comE ∆SigX	**-**	**-**	ComE pMR1ΔcomX

### Identification of a cin-box in the promoter region of *comE*

**Fig 6:**
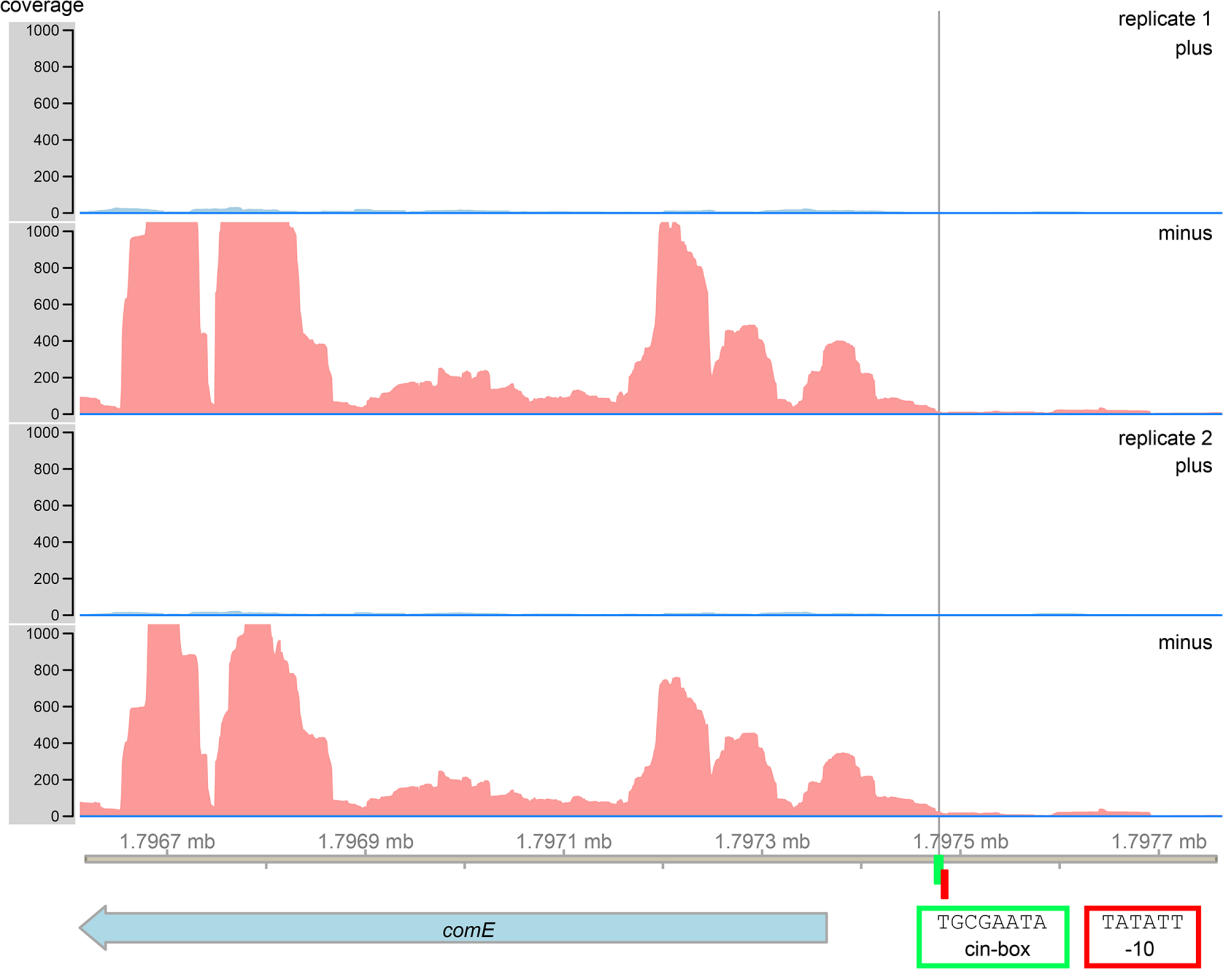
RNAsequencing and bioinformatics analysis of the 5`UTR of the *comE* gene of *S*. *mutans*. A SigX binding motif (cin-box, highlighted in green) was identified 109 bp upstream of the *comE* start codon. Directly adjacent to the cin-box a Pribnow box (highlighted in red) was found (red corners). RNA sequencing data revealed the transcriptional start site of the *comE* gene (black line).

We searched for the cin-box, a known binding motif for SigX [38], in the 5´ UTR of *comE*. Indeed we found a sequence 109 bp upstream from the start codon of *comE* which has only one mismatch with the consensus sequence of the cin-box (T**G**CGAATA, Fig 6). The late competence gene *lytFsm*, which is controlled by SigX [32,39] and is highly expressed (our data) also showed one mismatch at the same position (T**C**CGAATA), thus we conclude that the cin-box homologue identified upstream of *comE* is most likely functional. We then looked for a promoter sequence. Directly adjacent to the cin-box a -10 region (Pribnow box) was found (TATATT). A corresponding -35 region was not identified. The transcriptome analysis allowed to identify the transcriptional start site of the *comE* gene. It was localized directly downstream of the cin-box, in complete agreement with the -10 localization of the Pribnow box. Sequence analysis and RNAseq thus strongly suggest that SigX can bind to the 5`UTR of *comE* and induce transcription.

### Control of bacteriocin transcription and synthesis by XIP

If XIP induces *comE* expression via SigX it should also induce bacteriocin expression. We analysed the transcription of the mutacin V encoding gene *cipB* in the cipB pMR1 reporter strain with different gene deletion backgrounds in CDM medium under XIP induced conditions. If SigX controls *comE* and thus *cipB* transcription, then fluorescence of the reporter should be induced by XIP, but not in a Δ*comRS* or Δ*sigX* background, and independent of CSP signaling.Table 3 (and microscopic images in S13 Fig) shows that the expression of *cipB* required the presence of *comE*,*comRS* and *sigX*, and was weakened in the Δ*comD* background. As the deletion of *comE* completely abolished *cipB* expression we can exclude an influence of other bacteriocin regulatory systems. The results on the CSP induced comE and cipB reporter strains in THBY are shown in S5 Table and S6 Table. Thus we conclude that SigX indeed induces transcription of bacteriocins in *S*. *mutans* via ComE. This is a novel role for SigX, therefore we asked if bacteriocins could be found in the culture supernatant.

**Table 3:** Expression of *cipB* in different gene deletion background in CDM under XIP induced conditions. In the genetic background of the fluorescent reporter strain CipB pMR1 deletion mutants for Δ*comC*, Δ*comD*, Δ*comE*, Δ*comS* and Δ*comRS* were constructed, cultivated in CDM medium and induced by XIP (2 μM). Further explanations as in Table 1.

CDM			
Reporter genotype	2 μM XIP	Control	Strain
CipB	**✓**	**-**	CipB pMR1
CipB ∆comC	**✓**	**-**	CipB pMR1ΔcomC
CipB ∆comD	**(✓)**	**-**	CipB pMR1ΔcomD
CipB ∆comE	**-**	**-**	CipB pMR1ΔcomE
CipB ∆comS	**(✓)**	**-**	CipB pMR1ΔcomS
CipB ∆comRS	**-**	**-**	CipB pMR1ΔcomRS
CipB ∆SigX	**-**	**-**	CipB pMR1ΔcomX

As a positive control, we analysed CSP induced bacteriocin production in THBY. CSP induction caused clear inhibition zones of the overlaid indicator strains *L lactis* and *S*. *sanguinis*, which are sensitive for mutacins V and IV, in the wild-type and the Δ*comC* and Δ*comS* mutant, respectively (S17 Fig). Deletion of *comC* could be complemented by externally added CSP, and deletion of *comS* had no effect as expected. We then tested bacteriocin production on CDM agar plates. No inhibition of the indicator strains could be observed, neither for CSP nor for XIP stimulated cultures. Bactericidal activity was also not detected in the concentrated supernatants of XIP induced and planktonically growing *S*. *mutans* strains in CDM. Since bacteriocins were clearly transcribed under these conditions, various mechanism could account for the lack of bacteriocidal activity: Bacteriocin synthesis could be regulated posttranscriptionally, they might not be secreted, or they could be degraded. Interestingly also CSP was either not produced, degraded or not secreted (S18 Fig and S1 Text).

### Population heterogeneity and timing of bacteriocin expression

Our data show that bacteriocin expression in *S*. *mutans* is controlled by two different mechanisms: (1) A fast unimodal post-transcriptional activation of ComE by CSP independent from the growth medium. It is likely caused by phosphorylation of the response regulator upon CSP detection by the ComDE two-component system. (2) A delayed activation of *comE* transcription by XIP in CDM mediated by SigX, and thus showing the same modality as SigX.

**Fig 7:**
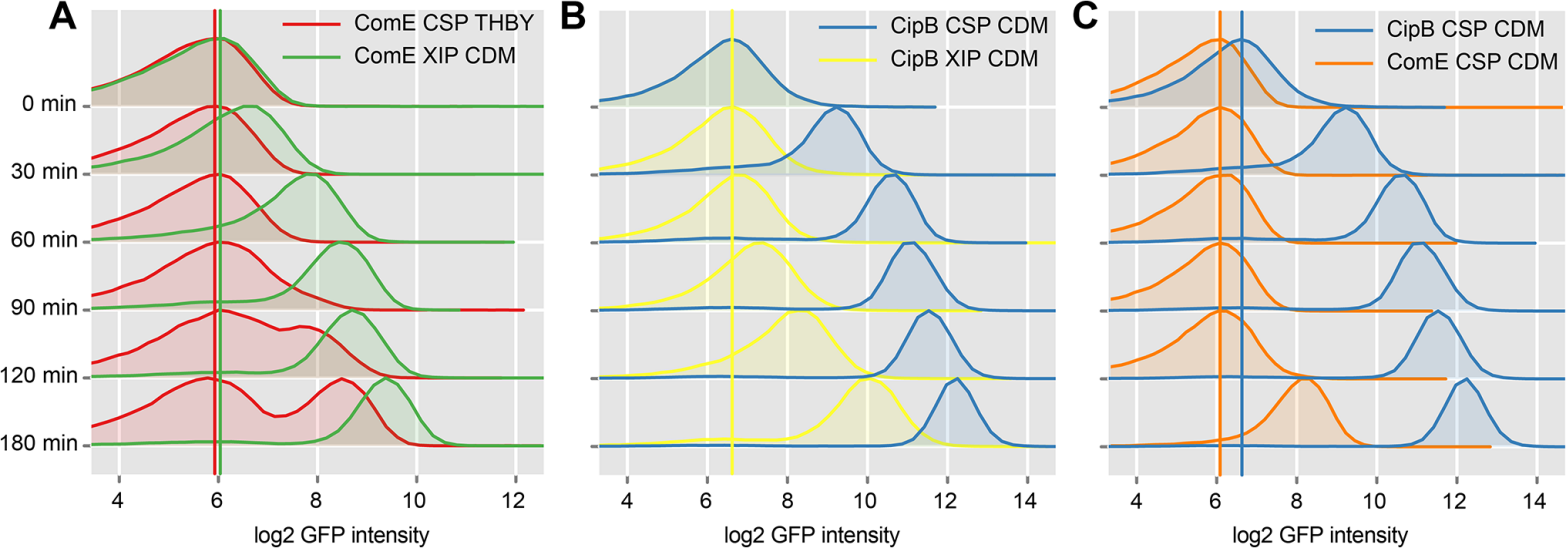
Time resolved analysis of *comE* and *cipB* expression using flow cytometry. CipB pAE03 and ComE pAE03 GFP reporter strains were grown in CDM or THBY and were induced with 2 μM CSP or 2 μM XIP. 0, 30, 60, 90, 120 and 180 minutes post induction samples were taken and analyzed using a flow cytometer. Samples from uninduced controls were taken at corresponding timepoints. Density plots of GFP fluorescence (log2 units) are shown during time. In **A** the density plots of the XIP-induced ComE pAE03 reporter strain grown in CDM (green plots) are compared with the density plots derived from the same strain grown under CSP-induced conditions in THBY (red plots). The colored vertical lines represent the peak log2 GFP fluorescence intensity of the distribution at timepoint 0h. In part **B** density plots derived from the XIP induced CipB pAE03 reporter strain grown in CDM (yellow plots) are plotted against the density plots of the same strain grown in the same medium under CSP induced conditions (blue plots). **C** showed the comparative analysis of the ComE pAE03 reporter strain (orange density plots) and the CipB pAE03 strain (blue density plots) both grown in CDM under CSP (2μM) induced conditions.

To investigate the timing and modality of bacteriocin expression, we analyzed CSP and XIP mediated signaling using time-resolved flow cytometric analysis of the reporter strains for *comE* and its immediate target, *cipB*. The density plots of uninduced *comE* and *cipB* reporter strains (controls) can be found S19 Fig. Fig 7A shows that *comE* transcription was unimodal in CDM using XIP as an inducer (green density plots), but bimodal in THBY under CSP inducing conditions (red density plots). Exactly the same behavior had previously been observed for *sigX* [15]. Apparently the modality of *sigX* expression determines whether *comE* expression is uni- or bimodal.

The induction of *comE* expression by XIP in CDM starts faster (30–60 minutes post XIP addition) than the CSP induced transcriptional activation of *comE* expression in THBY which begins 90–120 minutes post induction. CSP causes medium independent unimodal expression of bacteriocins, as also shown above. We have also shown above, that addition of CSP to THBY allows XIP signaling, but not because of a direct regulatory link as previously hypothesized. Instead an indirect effect is operating. We suggest that bacteriocins make the cells permeable and thus allow reimport of XIP into the producing cell. Thus in our experiment here, bacteriocin synthesis is induced by CSP, likely resulting in permeabilzation of the cell and reimport of XIP that is endogeneously produced. XIP then activates *sigX*, resulting in transcription of *comE* via binding of the SigX-RNA-Polymerase complex to the cin-Box. This explains why *comE* expression is delayed in THBY by about 90 min in comparison to CDM.

Fig 7B clearly shows that the bacteriocin encoding gene *cipB* was strongly and instantaneously induced upon CSP stimulation in CDM in an unimodal way (blue density plots). Already 30 minutes after induction, which corresponds to the maturing time of the GFP+ fluorophore, the expression of *cipB* was significantly enhanced. Addition of XIP in CDM medium (yellow density plots) also induced *cipB* expression unimodally but more weakly and significantly delayed. It starts 90 minutes after addition of XIP and reaches its maximum 180 minutes post induction. Independent from the growth medium, induction of *cipB* expression occurs within 30 min post CSP supplementation (S20 Fig). Thus, the data are consistent with the proposed transcriptional control of *comE* by SigX.

To observe post-transcriptional and transcriptional regulation in more detail, we then compared the time-course of expression of *comE* with that of its direct target *cipB* under CSP induced conditions in CDM directly (Fig 7C). *CipB* expression was observed instantaneously already 30 minutes post CSP supplement (blue density plots), while *comE* expression was unchanged and started to increase only 180 min post CSP addition (orange density plots). Microscopic analysis of the *comE* and *cipB* reporter strains confirmed that *comE* is not induced within the first 120 min while its target *cipB* is already fully induced 30 min post induction by CSP in CDM (S14 Fig and S1 Text). Thus CSP-induced activation of the bacteriocins via ComDE must be mediated via post-transcriptional regulation. The basal level of ComE transcripts is apparently sufficient for this reaction.

**Fig 8:**
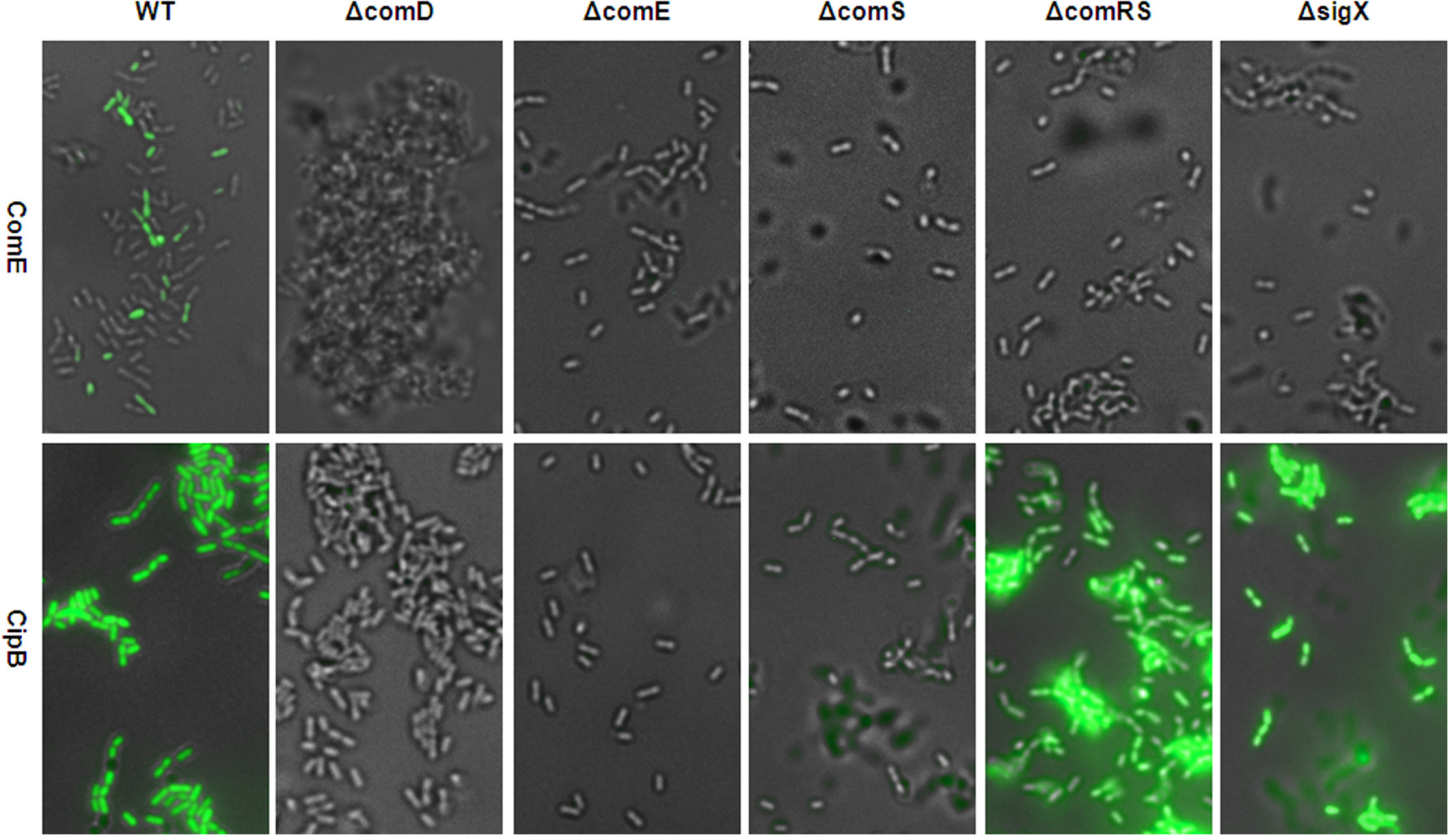
Modality of CSP induced *comE* and *cipB* expression in THBY in different gene deletion backgrounds. ComE pMR1 and CipB pMR1 reporter strains were grown in THBY under CSP (2 μM) induced conditions. 3 h post induction images were collected using fluorescence microscopy. GFP fluorescence images (green) of the reporters were overlaid with the phase contrast (grey) images of the corresponding strain. Images of the *comE* reporter strain are shown in the upper panel, while images of the *cipB* reporter strain are shown in the lower panel.

We then investigated the influence of the key genes in the signalling cascade on timing and modality of *comE* and *cipB* expression by analyzing the fluorescence of the *comE* and *cipB* reporter strains in different gene deletion backgrounds. Fig 8 shows that transcriptional activation of *comE* requires *comRS*, *comS* and *sigX* while the transcriptional activation of the ComE target *cipB* is independent of these genes. Moreover transcription of *comE* is bimodal while the transcription of its target *cipB* is unimodal. These findings confirm that indeed two different mechanisms (post-transcriptional and transcriptional regulation) are operating that activate ComE thus fully supporting our hypothesis.

### Posttranscriptional activation of ComE by phosphorylation

To determine if phosphorylation of ComE controls transcription of the bacteriocin encoding genes we constructed mutants of *comE* where the aspartate residue at position 60 was replaced by a phosphomimetic, namely by glutamate (D60E) or alanine (D60A). Glutamate enhances the negative charge of the side chain and thus mimics a phosphorylated aspartate. Conversely, alanine cannot be phosphorylated and thus ComE should be inactive under all circumstances.

**Fig 9:**
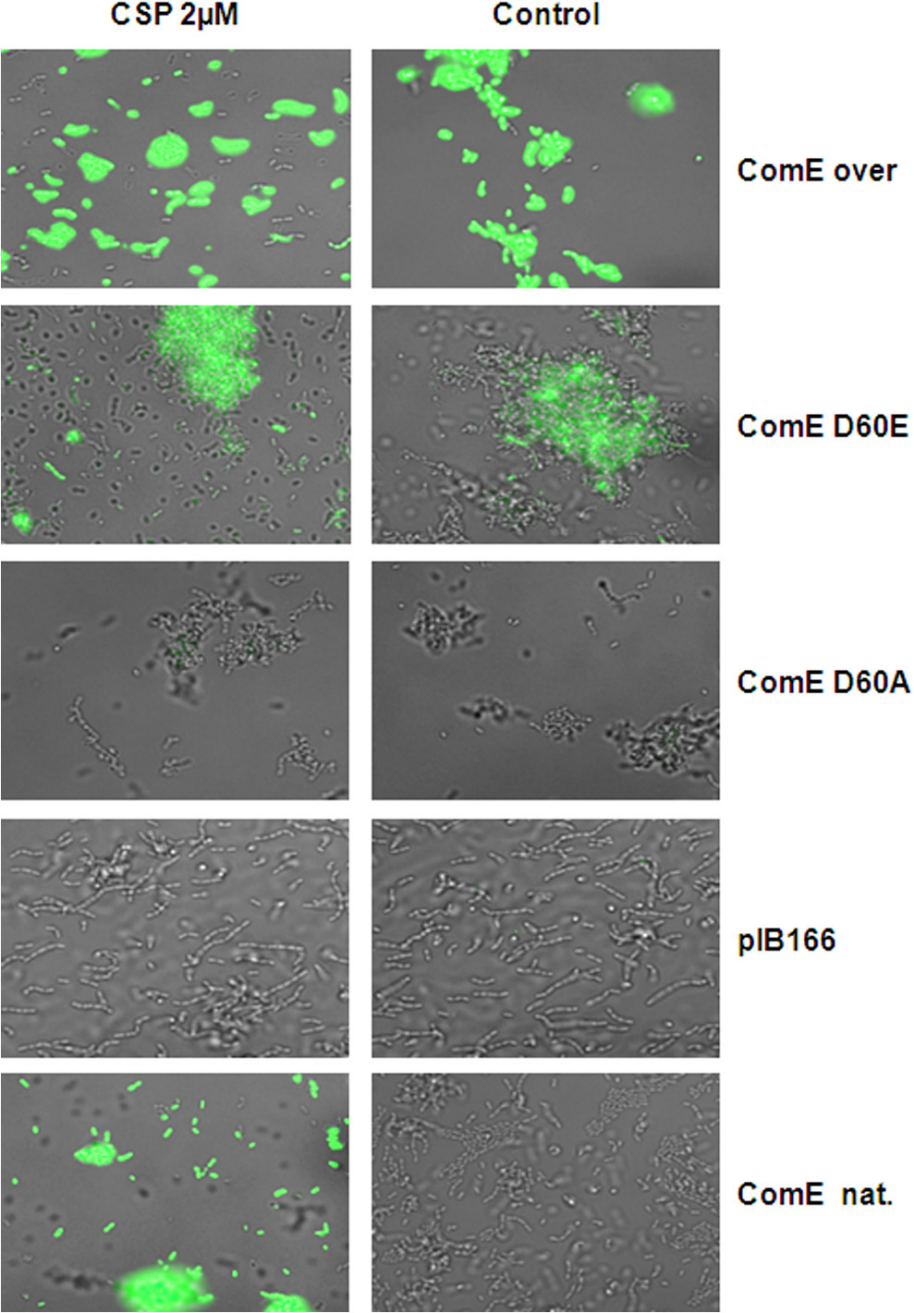
Phophorylmimetic study. A CipB reporter strain in the Δ*comE* deletion background was transformed with plasmids carrying the coding sequence of the natural *comE* (ComE nat.), the coding sequence of *comE* under the control of the strong lactococcal P23 promoter (P23-ComE), or the mutant genes ComE D60E (mimicking stable phorphorylation) and ComE D60A (no phosphorylation possible). A strain transformed with the empty vector (pIB166) was used as negative control. All strains were grown in THBY and fluorescence microscopy was performed 1 h after addition of CSP. Overlays of phase contrast and green fluorescence microscopic images are shown. The right column shows the un-induced controls.

We utilized the CipB pMR1 reporter strain in the Δ*comE* deletion background and transformed it with replicative pIB166 [37] based plasmids carrying either the native or a mutated *comE* gene under the control of the native *comE* promoter. As a control, we introduced the native *comE* gene into the Δ*comE* reporter strain, which restored the CSP responsive phenotype. We also introduced a *comE* overexpression plasmid into the reporter strain and found CSP independent unimodal *cipB* expression. Reporter strains carrying the empty plasmid showed no detectable fluorescence during growth or CSP induction (Fig 9). As expected, strain CipB pMR1 ΔcomE D60A did not fluoresce either under CSP induced or uninduced conditions. Due to the D60A mutation ComE could not be phosphorylated and looses its ability to induce *cipB* expression. In the CipB pMR1 ΔcomE D60E strain, which mimicked stable phosphorylation, *CipB* induction was independent of the presence of CSP, in contrast to the native *comE* gene. The fluorescence of the D60E mutant was lower than that of the CSP-induced wild-type. Hung *et al*. found that ComE phosphorylation affected dimerization of *S*. *mutans* ComE *in vitro* and that the D60E mutation prevents that dimerization [40]. To conclude here we demonstrate that phosphorylation of ComE is the mechanism responsible for the induction of bacteriocin expression under CSP induced conditions.

## Discussion

### Model of competence development in *S*. *mutans*

**Fig 10:**
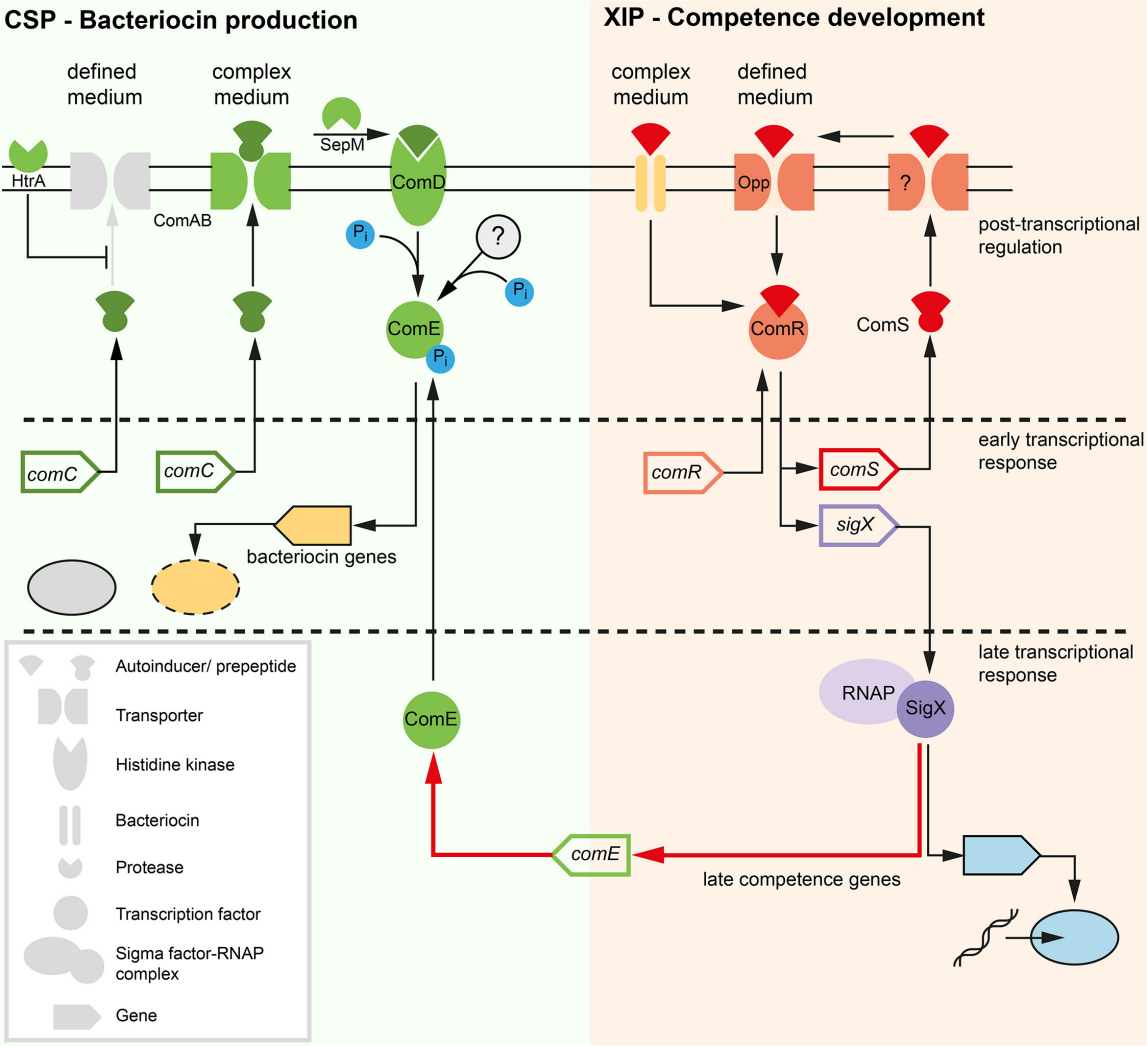
Model of competence development in *S*. *mutans*. Two quorum sensing pathways are operating in *S*. *mutans*. Bacteriocin expression is regulated via CSP signaling and the ComDE two-component system (green box). CSP should be renamed MIP (mutacin inducing peptide). Competence development is regulated via XIP signaling and the ComR regulator (red box). Both systems are connected via the alternative sigma-factor SigX which controls *comE* transcription (red arrow). Competence regulation proceeds in three successive steps: (1) Instantaneous post-transcriptional activation of the regulator ComR. (2) Early transcriptional response: Transcription of *comS*, resulting in a positive feedback loop for signal synthesis, and of *sigX*. (3) Late transcriptional response: Transcription of the SigX regulon, including the competence genes and the *comE* response regulator. Upon competence development the medium determines via which mechanism XIP is imported into the cell. In defined medium XIP is specifically imported via the Opp permease, leading to unimodal competence development. XIP import in complex medium requires bacteriocin activity and might be mediated via permeabilization of the cells. In complex medium, competence development is bimodal. Active bacteriocins and CSP areexclusively observed in complex medium. Post transcriptional mechanisms must be operating in the defined medium, e.g. down-regulation of bacteriocin translation, lack of secretion, or degradation, since the cells sense externally added CSP and transcription of bacteriocin encoding genes is induced.

Our analysis results in a new understanding of the QS regulatory network in *S*. *mutans* which is depicted in Fig 10. The two signals CSP and XIP control different traits: CSP signaling (green box) mediates bacteriocin expression while XIP signaling induces competence development (red box). The model contains three different temporal layers of signaling: (I) Fast post transcriptional activation of the response regulator ComE and the transcription factor ComR induced by their respective signals within the first 5 min after detection. ComE is activated by phosphorylation, and ComR is activated by binding of XIP. (II) Early transcriptional response of the activated regulators resulting in bacteriocin synthesis (CSP signaling) and transcription of the *comRS* genes and the alternative sigma-factor *sigX* (XIP signaling). (III) Late transcriptional response mediated by the alternative sigma-factor SigX. Transcription of the gene encoding the regulator of bacteriocin synthesis, *comE*, is controlled by SigX. In such a way both QS systems are connected. SigX controls both competence development and—via *comE*–bacteriocin synthesis. In the competence cascade *comE* is localized downstream of *comRS* and *sigX* and not upstream as previously thought. ComE could thus be viewed as a “late competence gene”, although it is only indirectly involved in competence development. This model explains why co-expression of *comS*, *sigX*, *comE* and the late competence genes is observed and why the modality of *sigX* expression determines the modality of *comE*. Our model discriminates between post-transcriptional and transcriptional regulation, which has not been taken into account by previous modeling approaches.

Instead of two QS systems operating in parallel connected through a hypothetical regulator as assumed before (Fig 1), we have two QS systems operating independently, triggered by their respective signals CSP and XIP. The competence cascade, however, is linked to bacteriocin synthesis through the alternative sigma-factor SigX which controls *comE*. Thus, SigX induces competence, and with a time delay, also bacteriocin synthesis. Typically QS networks are organized hierarchically (e.g. in *Pseudomonas aeruginosa*) [41]. By contrast, in *Vibrio harveyi* three parallel input channels responding to three different autoinducer signals are integrated by a central response regulator [42]. The type of network architecture found here comprises two different input channels which are connected by the alternative sigma-factor SigX. SigX is stimulated directly by XIP and indirectly by CSP. This type of network architecture has not been found before.

A hierarchical cascade mediates competence. XIP is the signal inducing competence, independent from the growth medium. However the medium determines via which way XIP is imported into the cell. We hypothesize that bacteriocin expression is required to induce permeability of the cell for external XIP. CSP signaling is independent from XIP signaling and is mediated via phosphorylation of ComDE. CSP-induced unimodal activation of bacteriocin synthesis is regulated post-transcriptionally and occurs before the transcription of *comE* which is mediated via XIP signaling. CSP induces competence only indirectly. It is therefore not a competence inducing peptide and should be renamed MIP (mutacin inducing peptide). CSP signaling is also in principle independent from the cultivation medium, but in defined medium bacteriocin excretion is inhibited.The new model simplifies our understanding of QS in *S*. *mutans* and resolves the open questions initially formulated. We will subsequently discuss them point by point.

### Bimodality of competence development

Lemme *et al*. [14] postulated *comE* being the origin of bimodality upon CSP induced competence development. Here we clearly show that this not the case since strong overexpression of *comE* independent of its native promoter did not enhance the percentage of competent cells. For *comS* a positive feedback loop was identified, which is a prerequisite for bimodality to occur [7] and thus a model assuming bistable expression of *comS* was developed [15]. However, here we show that neither *comR* nor *comS* alone can induce unimodal competence development when they are strongly overexpressed. Both genes or rather their gene products are required. Bistable expression of *comS* is not necessarily required. How then is the bimodality established in the system? Besides a positive feedback loop, noise is the second critical determinant for bimodality [29,43]. We suggest that noise in *comR* and *comS* expression accounts for bimodality since the overexpression analysis showed that both are limiting factors for unimodal competence development. Bimodality would then be a feature of the quorum sensing network, and not necessarily the result of bistable expression of one component [44].

XIP is a secreted factor, and thus it is unclear how phenotypic heterogeneity can be stably established if all cells are able to sense XIP, not only the overproducers. Previously it was found that the mature XIP signal of *S*. *thermophilus* is not released into the medium, but remains cell associated [13]. Thus cells with higher *comS* expression levels can re-import more XIP resulting in the observed positive feedback loop.

### Link between CSP and XIP signaling

It was previously suggested that an unknown regulator transfers the CSP induced signal from ComDE to ComRS. Here we show that this regulator does not exist. If a regulator would be required for transmitting the CSP induced signal to ComRS, then it would be possible to overcome its effect by overexpression of *comRS*, which should result in unimodal competence development. However, here we show that overexpression of *comRS* does not induce competence at all in complex media.

We suggest that CSP induces competence only indirectly by allowing XIP to be imported into the cell in complex media. The following findings support this hypothesis: 1. Competence is not induced in CDM by CSP, although externally added CSP is fully active and not degraded as previously thought. 2. A small quantity of CSP is required to induce unimodal competence development in THBY in a strain overexpressing *comRS*. 3. An increase in the concentration of CSP does not increase the percentage of competent cells in this experiment, suggesting that CSP triggers a process required for competence to occur and is not inducing it directly. 4. The time-lag of 60–90 min between CSP addition and *sigX* transcription in THBY clearly supports an indirect activation mechanism. 5. Deletion of CipB completely abolished CSP and CSP/XIP induced expression of the late competence gene *lytFsm*. Our findings are in accordance with a recent study showing that deletion of the bacteriocin encoding gene *cipB* almost completely abolishes competence development through CSP stimulation [34]. Thus it was suggested that the CipB bacteriocin itself might act as a regulator of competence [39]. This scenario is highly unlikely, because a regulatory role for a bacteriocin has never been demonstrated. Moreover, bacteriocins are secreted into the environment and thus can hardly exert intracellular regulatory roles. Based on our findings we suggest that bacteriocins are required to induce permeability of the producer cell for external XIP.

It was shown that deletion of Opp has no influence on CSP induced competence development in complex medium in *S*. *mutans* [15]. Thus XIP import upon bacteriocin expression in complex medium is not mediated via Opp and its precise mechanism remains to be elucidated. Pore formation is the mode of action of many bacteriocins [45]. Via these pores the small XIP molecule might enter the cell. Alternatively some of the bacteriocin exporters might allow XIP import. Cell wall and membrane alterations which confer immunity to the bacteriocin producer cells against their own bacteriocin [45] may also account for the altered permeability of the cell for XIP. Presently it is not known how and by which system the XIP precursor is secreted and processed in *S*. *mutans* [7,46,47].

### Why is *comE* expression biomodal, while bacteriocins are synthesized by the whole population?

Bacteriocin synthesis is induced by post-transcriptional unimodal activation of the ComE response regulator. The CSP signal causes phosphorylation of the ComE protein, resulting in instantaneous unimodal bacteriocin transcription and synthesis. This process is not affected by transport processes across the membrane and therefore it is independent of the cultivation medium. By contrast, the transcription of the *comE* gene is controlled by SigX, which is regulated via the XIP signaling cascade. Therefore bimodal *comE* expression occurs much later (90–120 post induction).

### How is the medium effect on signaling mediated?

It was thought that external CSP is degraded by the HtrA protease in CDM, while this protease is inhibited by media components in THBY and thus allows CSP to remain intact [15]. Here we show that external CSP is not degraded in CDM. For the HtrA serine protease of *S*. *pneumonia*e it was demonstrated that it indeed cleaves the pneumococcal CSP. The enzyme was not inhibited by bovine serum albumin, but by denatured protein, prompting the authors to suggest that CSP signaling is a means to detect intracellular stress [22]. The CSP of *S*. *mutans* is more homologous to the BlpC peptide which induces bacteriocin synthesis than to the CSP autoinducer of *S*. *pneumoniae*. Extracellular BlpC is not degraded by HtrA [48] in accordance with the lack of degradation of *S*. *mutans* CSP in CDM. These data, too, demonstrate that CSP of *S*. *mutans* is functionally related to BlpC of *S*. *pneumoniae*.

### CSP is a mutacin stimulating peptide

Activated ComR represents the only regulator mediating competence in *S*. *mutans*. In a recent review it was proposed that the ComDE two-component system (TCS) of *S*. *mutans* should be renamed to Blp since it primarily regulates bacteriocin expression [1]. However, this suggestion was based on the situation in *S*. *pneumoniae*, where the BlpRH TCS is exclusively involved in regulating bacteriocin production [48]. In *S*. *mutans*, *comE* is controlled by the master regulator of competence SigX. Thus we would not propose to rename the system Blp. However, since CSP does not stimulate competence directly, and in *S*. *mutans comE* is not an early competence gene as the name suggests, we propose to rename CSP to MIP (mutacin inducing peptide) and ComDE to MutDE.

### How is ComE phosphorylated in the absence of a CSP signal?

Upon XIP induction SigX enhances the ComE level in the cell. To be active, ComE needs to be phosphorylated, yet the histidine kinase ComD is not receiving a signal under these conditions and its deletion still allows *cipB* expression [34]. Therefore non-cognate kinases must be able to phosphorylate ComE. Recently cross-talk between TCS systems was observed [49]. For the serine/threonine protein kinase PknB of *S*. *mutans* a regulatory role in bacteriocin expression was found [50], suggesting that this enzyme might also be able to phosphorylate ComE.

### Why are bacteriocins not secreted in CDM medium?

Although the transcription of bacteriocin encoding genes was strongly induced by externally added CSP or XIP in CDM, no bactericidal activity was found in the culture supernatants, suggesting that bacteriocin synthesis is regulated post-transcriptionally. This is supported by a study of two phenotypic variants (transparent/opaque) of the *S*. *pneumoniae* R6 strain [48]. They showed an identical transcriptional response to the bacteriocin inducer BlpC. However only for cells of the transparent phenotype bacteriocin activity could be detected, while none was found in the opaque phenotype. The authors demonstrated that the HtrA protease abolished bacteriocin synthesis post transcriptionally in these cells [48]. This might be the reason why we detected no CSP and no bactericidal activity in the CDM supernatants. HtrA activity likely prevents CSP and bacteriocin processing and/or secretion in CDM. Multiple environmental factors additionally influence bacteriocin expression in *S*. *mutans*, including cell density and nutrient availability [51].

### A novel role of SigX and its biological implications

The observation that *comE* transcription is under the control of SigX represents a novel regulatory role for SigX, which thus not only controls the expression of the transformasome, but also the synthesis of bacteriocins. In *S*. *pneumoniae* and *B*. *subtilis* the master regulators of competence, SigX and ComK respectively, exclusively regulate transformasome related genes [1,43]. Competence and bacteriocin production is uncoupled in these organisms. Coupling it like in *S*. *mutans* makes ecological sense, because it provides the genetic variability which makes competence an adaptive trait, as suggested previously [52]. In its natural niche *S*. *mutans* is part of the multispecies oral biofilm which consists of over 600 different species [53]. Thus *S*. *mutans* is faced with a strong competitive environment which is at the same time a rich source of genetic variability. In a dual species biofilm of *S*. *mutans* and *Candida albicans* the CSP and XIP triggered QS cascades were induced simultaneously [54]. The ComR regulator has a low stringency with respect to the exact sequence of the XIP peptide required for activation [12], and consequently, Streptococci respond to heterologous peptides [12,55]. Thus *S*. *mutans* might sense the presence of other Streptococci via XIP signaling and directly respond to this stimulus by producing bacteriocins and killing the competitor, while exploiting its genetic information at the same time.

To conclude, here we deciphered the complex QS signaling network of *S*. *mutans* on the single cell level. Competence is exclusively mediated via XIP signaling in a hierarchical network structure. We show that competence development is coupled to bacteriocin synthesis through the alternative sigma-factor SigX, which makes this QS network highly efficient for acquiring new genetic information in the competitive environment of dental biofilms.

## Methods

### Strains and media

All *S*. *mutans* strains were routinely propagated in in Todd Hewitt broth medium supplemented with 0.5% (wt/vol.) yeast extract (THBY; Becton Dickinson, Heidelberg, Germany) in an incubator (5% CO_2_, 37°C) without agitation. When indicated, antibiotics were added to the medium (chloramphenicol 10 μg/ml, tetracycline 12.5 μg/ml and erythromycin 10μg/ml). For experiments conducted in chemically defined medium (CDM) [56] a 10 ml overnight culture of the appropriate strain grown in THBY was centrifuged for 10 min at 5000 rpm and 4°C. The supernatant was removed and the cell pellet was gently resuspended in fresh CDM medium and again centrifuged as described above. After removal of the supernatant the pellet was finally gently resuspended in 5 ml of CDM. Strains were diluted to an OD of 0.1 and grown at 37°C and 5% CO_2_.

### Construction and verification of single and dual fluorescent reporter strains

The plasmids used for the construction of reporter strains (S1 Fig), an analysis of maturation times of various fluorescent proteins in *S*. *mutans* (S2 Fig), a scheme showing the integration of the plasmids into the chromosome (S3 Fig), comparison of expression levels of different late competence genes (S4 Fig), comparison of two LytFsm constructs with different fluorophores (S5B Fig), spectral separation of TagBFP2 and GFP+ (S6 Fig), dye swap of *comE* with the late competence gene *lytFsm* (S9 Fig), and construction of reporters in various gene deletion backgrounds are described in full detail in S1 Text, which also provides a discussion of the reporter constructs. A list of the constructed strains (S1 Table) plasmids (S2 Table) and primers (S3 Table) can be found in the supplements.

### Flow cytometric analysis

Overnight cultures of the strains grown in THBY were diluted to an OD of 0.1. For the strains intended to grow in CDM the above described washing procedure was applied to remove all traces of the complex media and finally strains were diluted to an OD of 0.1 and grown at 37°C and 5% CO_2_. When the bacteria had reached an OD of 0.15 the culture was divided into three equal fractions. One fraction was treated with 2 μM synthetic CSP, the second with 2 μM synthetic XIP, and the third fraction was used as an uninduced control. Samples (0.5 ml) were taken after 30, 60, 90, 120 and 180 minutes post induction. Samples derived from strains growing in either CDM or THBY were centrifuged (5 min and 7000 rpm) and washed once with PBS. Subsequently the strains were resuspended in 1 ml of ice-cold PBS and sonicated using a MS72 sonotrode with the Sonoplus HD2200 device (Bandelin, Germany) for at least 20 sec at 10% power. Settings were a 0.5 sec impulse which was followed by a 0.5 sec break. Live/Dead staining before and after sonication was performed to exclude that sonication significantly interfered with membrane integrity. For flow cytometry the LSR Fortessa Cell analyser (BD, Germany) was used. 0.22 μM filtered PBS was applied as sheath fluid. Cytometer settings were chosen as previously reported [14]. 50000 cells were analysed and the resulting data processed with a self-written R-Script.

### Construction of phosphomimetic mutants of ComE

The mutation of the *comE* gene was accomplished using a PCR-driven overlap extension approach [57]. The triplet encoding the aspartate residue at position 60 (GAT) was changed to GCT (Ala) and GAG (Glu), respectively, using the primers listed in S3 Table. In a first PCR the *comE* gene and its native promoter region were amplified in two separate parts thereby introducing the desired mutation via the 5`termini of the inner primers (primer pairs PcomE_F1/D60E_R1 and PcomE/D60A_R1 for the first part and primer pairs D60E_F2/ComE_R2 and D60A_F2/ComE_R2 for the second part of the *comE* gene. The two PCR amplified parts of *comE* contained homologous flanks of approximately 22 bp to each other and were used as template for a second PCR with only the outer primers (PcomE_F1/ComE_R2), amplifying the entire *comE* coding sequence including the native promoter. The PCR products were purified and cloned into the vector pIB166 [37] in opposite direction to the constitutive P23 promoter to allow transcription from the native promoter. The plasmid sequence was verified by sequencing and the plasmid transformed into the *S*. *mutans cipB* reporter strain in the Δ*comE* deletion background. As positive control the native *comE* sequence was cloned into pIB166 and the transformed empty vector was used as negative control.

### Overexpression strains in the LytFsm reporter background

To construct overexpression strains for ComD, ComE, ComR, ComS and ComR+ComS, independent from their native promoter, plasmid pIB166 containing the strong constitutive P23 promoter from *Lactococcus* sp. was used [37]. The coding sequences including the native ribosomal binding sites were PCR-amplified (see S3 Table) and cloned blunt end via the SmaI restriction site into the vector. The correct plasmid sequence was verified by sequencing and 100 ng of the plasmid transformed into the LytFsm reporter strain.

While this cloning approach was successful for *comE*, *comS*, *comRS* and *comR*, we were not able to obtain a correct plasmid sequence for *comD*. Point mutations and frameshift mutations were observed in the ribosomal binding site or the coding region of the gene and strongly suggest that this protein is toxic for *E*.*coli*. As it was already successfully cloned in *E*. *coli* under the control of the native promoter we assume that the P23 promoter has a significantly higher basal transcription than the native *comD* promoter in *E*. *coli*. Attempts to fuse the GtfB or GtfC promoter to the coding sequence of *comD* were also not successful, although these constructs could be easily obtained for *comE* and *comR*, respectively. We therefore used a PCR-driven overlap extension approach ([57]) to amplify a fusion construct consisting of five parts; the upstream region of gene SMU_1342, the P23 promoter, the *comD* gene, the chloramphenicol resistance cassette (CAT) and downstream flanking regions of SMU_1342. Homology flanks of the different parts were introduced via the 5`terminus of the PCR primers. This construct allows the integration of the *comD* gene under the control of the P23 promoter via double homologous recombination at the SMU_1342 locus. In a first PCR all parts of the fusion construct were amplified separately and purified using the PCR Purification Kit (Qiagen, Germany). Subsequently equal amounts (100 ng) of all different PCR products were used as template for a second PCR with primers UP1342_F/D1342_R spanning the entire construct (outer primers). The PCR reaction was directly used as template to transform the LytFsm reporter strain. Chloramphenicol resistant clones were selected on THBY agar plates, picked and cultivated in liquid THBY medium containing 10 μg/ml chloramphenicol. Genomic DNA was isolated from 2 ml of the exponentially growing culture and correct constructs were verified by PCR using the primer pairs UP1342_F/D1342_R and P23_F/ CAT_R).

### Analysis of gene expression

A detailed description of methods for RNA extraction, RNA sequencing and qRT-PCR can be found in the S1 Text. Raw and processed RNAseq data have been deposited in the gene expression omnibus database (http://www.ncbi.nlm.nih.gov/geo/) under accession number GSE65982.

### Bacteriocin overlay assay

O/N cultures of *S*. *mutans* WT, Δ*comS* and Δ*comC* deletion strains with a starting OD600 of 0.1 were grown in CDM or THBY until the cultures reached an OD600 = 0.25. Then the cultures were split and induced with either 2 μM XIP or 2 μM CSP. Uninduced cultures were used as controls. After 2 hours of further growth (37°C, 5% CO2) 2.5 μl of the culture were spotted on CDM or THBY agar plates. The plates were incubated for further 4 h until the induced cultures were overlaid with a 2.5 μl drop of a 2 μM CSP (or 2 μM XIP) solution in CDM (THBY). The drops were allowed to soak in the plate. Finally the plate was incubated for 24 h at 37°C and 5% CO2. Overnight cultures of the indicator strains *S*. *sanguis* and *L*. *lactis* were diluted 1:200 in fresh CDM Top Agar (0.7% Agar) placed at 37°C and 5 ml of the mixture was poured over the agar plates containing the spotted *S*. *mutans* producer strains. The overlaid plate was incubated for further 16 h in a CO_2_ incubator. Zones of inhibition were documented using a conventional camera.

## Supporting Information

S1 Fig Integrative reporter plasmid system for single cell analysis in *S*. *mutans*. Based on plasmids pMR1 and pMR2 dual fluorescent reporter strains were established.
(TIF)

S2 Fig Slow maturation kinetics of reporter strains expressing mCherry. In the upper panel microscopic overlay images of a *comS*_*p*_-*mCherry* reporter strain are shown 2, 4, 8 and 16 hours after induction of gene expression with CSP. In the middle panel the same analysis is shown for a *cipB*_*p*_ *-mCherry* reporter strain. Overlay images of a CipB pAE03 (*cipB*_*P*_ *gfp+*) reporter strain taken at corresponding timepoints are shown in the lower panel.
(TIF)

S3 Fig Genomic context of the LytFsm pMR1 LytFsm pMR2 double reporter strain. The reporter strain is constructed by a sequential double integration of the plasmids LytFsm pMR1 and LytFsm pMR2 into the same genomic locus. After chromosomal integration of the LytFsm pMR1 plasmid via single homologous recombination 3 different homologous sites are present in the genome allowing integration of the LytFsm pMR2 plasmid. Integration at the different sites (1–3) results in a slightly different genomic context for the resulting constructs. In all cases the second integration results in reporter strains carrying TagBFP2 and gfp+, each under the control of the LytFsm promoter.
(TIF)

S4 Fig The *lytFsm-gfp+* reporter strain showed the highest signal to noise fluorescence ratio of all tested single fluorescent reporter strains specific for late competence genes. Microscopic overlay images of GFP+ reporter strains for late competence genes *smu_498*, *smu_625*, *smu_644*, *lytFsm*, *smu_1001* and *smu_1987* are shown. Images were taken 3 hours after induction of gene expression with CSP.
(TIF)

S5 Fig Single cell co-expression analysis of *comS* with *sigX* (A) and comparison of two LytFsm constructs with different fluorophores (B). In (**A)** overlay images of a CSP-induced dual fluorescence reporter strain are shown which expresses TagBFP2 under the control of the *comS*-promoter and GFP+ under the control of the *sigX*-promoter. In the left column the TagBFP2 channel, in the middle column the GFP+ channel and in the right column the overlay images of the green and the blue channels are shown. In (**B)** overlay images of CSP induced (2μM) LytFsm pMR1 LytFsm pMR2 dual reporter strain are shown. The strain carries TagBFP2 and GFP+ both under control of the identical promoter (LytFsm). Images were taken 3 h after induction of gene expression by CSP.
(TIF)

S6 Fig Spectral separation of TagBFP2 and GFP+. Two *cipB* reporter strains, one expressing TagBFP2 and the other GFP+ were analyzed using both the green and the blue light cube of an Evos^R^ fluorescence microscope. Reporter strains were grown in THBY and induced with 2μM CSP. 3 h post CSP induction images were collected. Phase contrast and fluorescence overlay images are shown. No spectral crosstalk into the other channel was observed for both reporter strains.
(TIF)

S7 Fig Influence of the CSP inducer concentration on the expression of a LytFsm reporter strain. A LytFsm pAE03 reporter strain grown in THBY medium was induced with different concentration of CSP. 3h after induction samples were taken and analyzed using flow cytometry. 50000 individual cells were analyzed to determine the GFP intensity distribution of the population.
(TIF)

S8 Fig Influence of the CSP inducer concentration on the expression of a LytFsm reporter strain. A LytFsm pAE03 reporter strain grown in THBY medium was induced with different concentration of CSP. 3h after induction samples were taken and the fluorescence was visualized under the fluorescence microscope. Overlay images of the GFP fluorescence and phase contrast are shown.
(TIF)

S9 Fig Dye swap. Single cell co-expression analysis of *comE* with the late competence gene *lytFsm*. In (**A)** overlay images of a dual fluorescence reporter strain are shown which expresses tagBFP2 under the control of the *comE*-promoter and GFP+ under the control of the *lytFsm*-promoter. In the left column the TagBFP2 channel, in the middle column the GFP+ channel and in the right column the overlay images of the green and the blue channels are shown. In (**B)** a dye swap of the dual reporter was performed, thus tagBFP2 expression is under the control of the *lytFsm* promoter while gfp+ expression is controlled by the promoter of *comE*. Images were taken 3 h after induction of gene expression by CSP.
(PNG)

S10 Fig Overexpression of *comRS* in a lytFsm reporter strain background in defined medium. A LytFsm comRS (comRS overexpr.) reporter strain carrying the *comRS* genes under control of the strong constitutive P23 promoter was grown in chemically defined medium. As controls reporter strains expressing wild-type levels of comRS, LytFsm pAE03 (control) and LytFsm pIB166 (plasmid control) were cultivated under the same conditions. Cells were harvested after the cultures reached an OD_600_ = 0.2 and analyzed using fluorescence microscopy. The overlay images (gfp fluorescence/phase contrast) are shown.
(TIF)

S11 Fig Overexpression analysis of *comE*, *comR*, *comS* and *comRS* in the LytFsm reporter strain background. Expression of the different genes was under the control of the strong constitutive lactococcal P23 promoter on the replicative plasmid pIB166. LytFsm pAE03 derived overexpression strains were grown in THBY and induced with 2μM CSP. 3 h post CSP supplementation samples were taken and images were collected using fluorescence microscopy. In the left column overlay images (gfp/phase contrast) of the CSP induced strains are shown while in the right column the overlays of the un-induced strains are presented.
(TIF)

S12 Fig Combined effect of CSP and XIP on *lytFsm* expression in complex medium in a *cipB* deletion background. The LytFsm pAE03 and LytFsm pAE03 ΔCipB reporter strains were grown in complex medium until the culture reached an OD_600_ of 0.2. The culture was divided and induced with either 20 μM CSP or 20 μM XIP alone or a combination of 20 μM XIP and 20 μM CSP. 3 h post induction cells were harvested and analyzed using fluorescence microscopy. Overlay images (phase contrast and green fluorescence) of the collected images are shown.
(TIF)

S13 Fig Single cell reporter strain analysis of CSP-and XIP induced *cipB* expression in CDM medium in different gene deletion backgrounds. Overlay microscopic images were recorded 3 h after induction. Un-induced controls of the reporter strains are shown in the bottom row (CON).
(TIF)

S14 Fig Time course of the CSP induced expression of a CipB and ComE reporter strain in CDM. Fluorescent CipB pMR1 and ComE pMR1 reporter strains were grown in CDM under CSP induced (2μM) conditions. Fluorescent and phase-contrast images were collected 30, 60, 90 and 120 minutes post CSP addition. Overlay images are shown.
(TIF)

S15 Fig Time-resolved analysis of *comE* and *cipB* expression using quantitative RT-PCR. Expression of *cipB* and *comE* in CDM medium 5 (grey bars) and 15 minutes (red bars) post induction with either 2 μM CSP or 2 μM XIP. Fold changes were calculated relative to the time-point immediately before induction (t = 0 min). The error bars indicate the standard deviation from three independent biological experiments.
(TIF)

S16 Fig Single cell reporter strain analysis of CSP-and XIP-induced *comE* expression in CDM medium in different gene deletion backgrounds. Overlay microscopic images were recorded 3 h after induction. Un-induced controls of the reporter strains are shown in the bottom row (CON).
(TIF)

S17 Fig Bacteriocin overlay assay of *S*. *mutans* strains grown on THBY (A) and CDM (B) agar plates. CSP (2 μM) or XIP (2 μM) induced *S*. *mutans* WT, Δ*comC* and Δ*comS* deletion strains were grown on THBY or CDM agar. Uninduced strains were used to detect self-induced expression of bacteriocins upon growth. After 24 h incubation of the spotted producer strains plates were overlaid with the *L*. *lactis* indicator strain diluted in either THBY top agar (A) or CDM top agar (B). In (A) an image of the overlaid THBY agar plate is shown. In the left column (+CSP) strains induced with 2 μM CSP are spotted while in the right column (-CSP) the corresponding uninduced strains are shown. (B) shows an image of the CDM agar plate, the left column contains the CSP or XIP (+CSP/+XIP) induced and spotted strains while the corresponding uninduced strains (-CSP/-XIP) were spotted in the right column.
(TIF)

S18 Fig Accumulation of endogenously produced autoinducers upon growth in CDM. CipB pMR1 and comE pMR1 reporter strains in different gene deletion backgrounds were grown in CDM until the cultures reached the stationary growth phase (8h growth). Cells were harvested and fluorescence images collected using fluorescence microscopy. Overlay images (gfp fluorescence/phase contrast) of the different cipB pMR1 (upper panel) and comE pMR1 (lower panel) reporter strains are presented.
(TIF)

S19 Fig Time resolved flow cytometric analysis of *comE* and *cipB* expression in CDM and THBY. Uninduced CipB pAE03 (left column) and ComE pAE03 (right column) fluorescent reporter strains were grown in CDM (upper panel) and THBY (lower panel). At timepoints corresponding to 0, 30, 60, 90, 120 and 180 minutes post induction (see Fig 5) samples were taken and analysed using flow cytometry. The distribution of the gfp fluorescence for 50.000 analyzed cells is shown.
(TIF)

S20 Fig Time resolved flow cytometric analysis of *cipB* expression in CDM and THBY. The CipB pAE03 reporter strain was either grown in THBY and induced with 2 μM CSP (violet plots) or grown in CDM and induced with 2 μM XIP (yellow plots). 0, 30, 60, 90, 120 and 180 minutes post induction samples were taken and analysed using flow cytometry. The gfp fluorescence of 50.000 cells was analyzed for each condition and the corresponding density plots (log2 gfp fluorescence intensity) are shown in the course of time.
(TIF)

S1 Table Strains used in this study. (DOCX)

S2 Table Plasmids used in this study. (DOCX)

S3 Table Primers used in this study. (DOCX)

S4 Table Expression of *comE* in different gene deletion background in CDM under CSP induced conditions. The fluorescent reporter strains carrying the CipB pMR1 plasmid in the chromosome were constructed in backgrounds of Δ*comC*, Δ*comD*, Δ*comE*, Δ*comS* and Δ*comRS* deletion strains, cultivated in CDM medium and induced by CSP (2 μM). Fluorescence microscopic images were obtained 3 hours after induction. Uninduced strains were used as controls. Tickmarks indicate detected fluorescence, tickmarks in brackets indicate weak fluorescence, a minus indicates no detectable fluorescence.
(DOCX)

S5 Table Fluorescence microscopic analysis of CSP induced *comE* fluorescent reporter strains in THBY. ComE pMR1 reporter strains with different gene deletion backgrounds were grown in THBY medium under CSP induced (2 μM) conditions. Additional deteails as in Table S4.
(DOCX)

S6 Table Fluorescence microscopic analysis of CSP induced *cipB* fluorescent reporter strains in THBY. CipB pMR1 reporter strains with different gene deletion backgrounds were grown in THBY medium under CSP induced (2 μM) conditions. Details as in Table S4.
(DOCX)

S1 Text Supplementary materials and methods, results and discussion. (DOCX)
