# Supplementary figures and images for: The Alternative Sigma Factor SigX Controls Bacteriocin Synthesis and Competence, the Two Quorum Sensing Regulated Traits in Streptococcus mutans
Source: PLoS Genet. 2015 Jul 9;11(7):e1005353. doi: 10.1371/journal.pgen.1005353 (PMC4497675; doi:10.1371/journal.pgen.1005353)

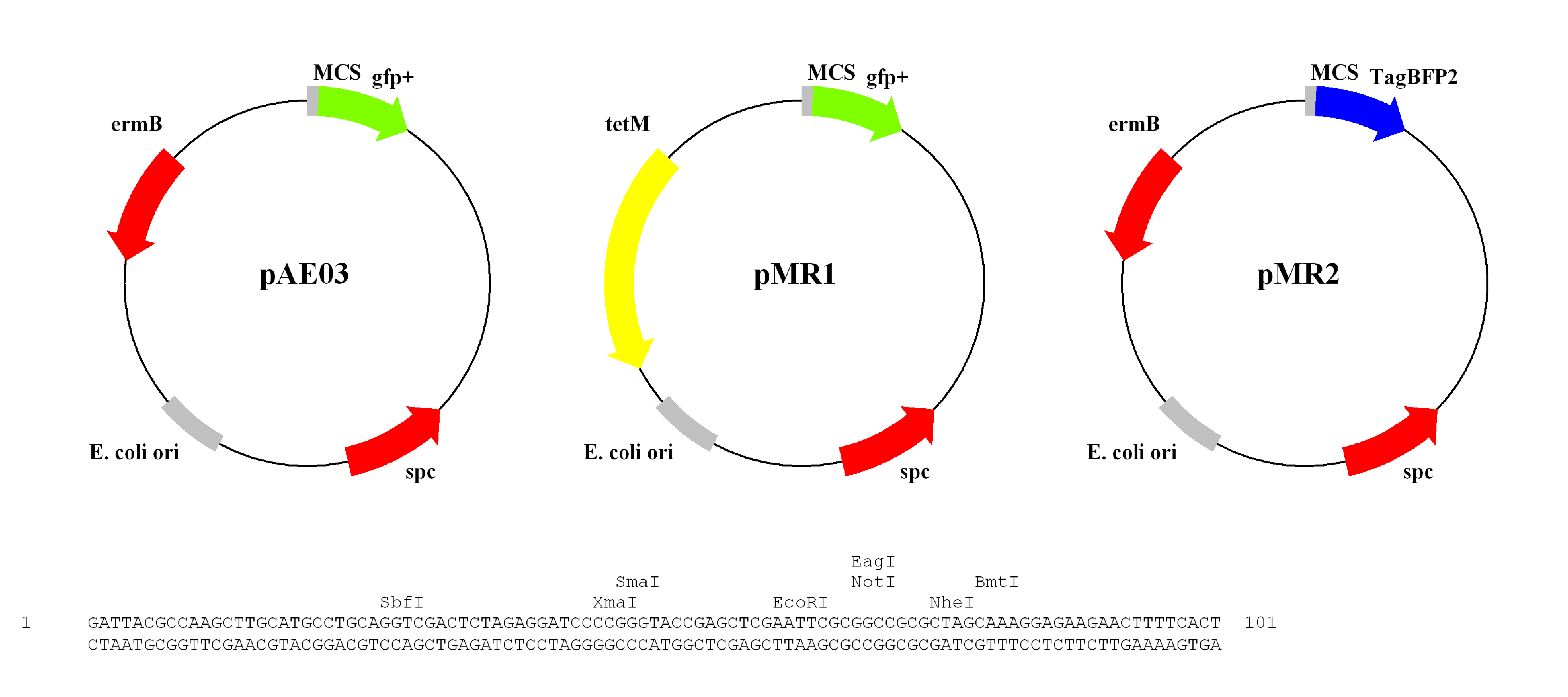

Supplement: S1 Fig — Based on plasmids pMR1 and pMR2 dual fluorescent reporter strains were established. (TIF) [file pgen.1005353.s001.tif]

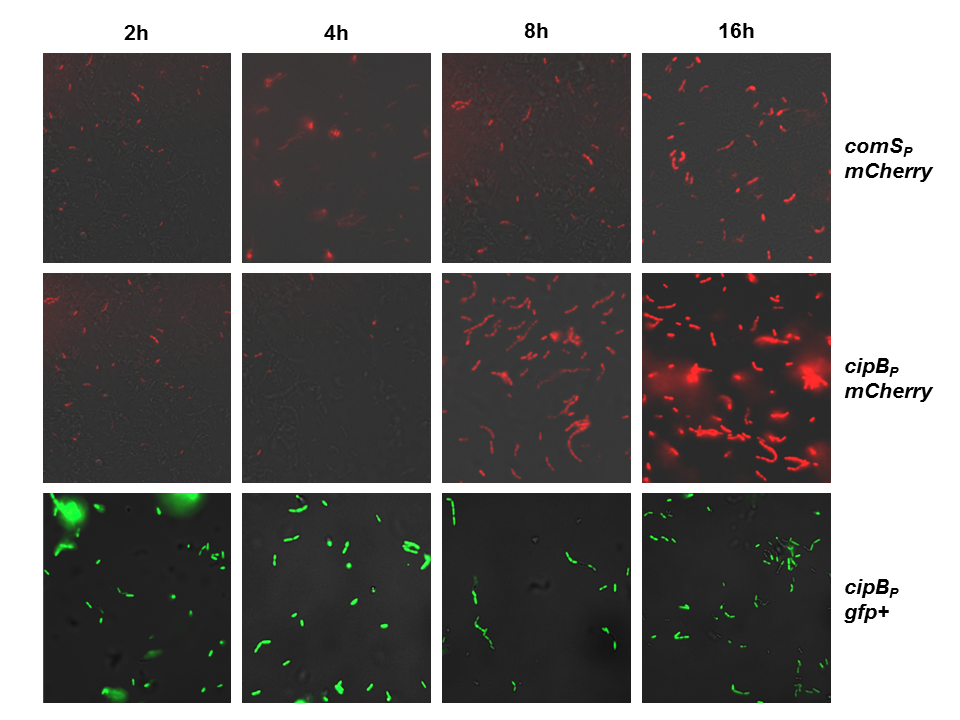

Supplement: S2 Fig — In the upper panel microscopic overlay images of a comS p-mCherry reporter strain are shown 2, 4, 8 and 16 hours after induction of gene expression with CSP. In the middle panel the same analysis is shown for a cipB p -mCherry reporter strain. Overlay images of a CipB pAE03 (cipB P gfp+) reporter strain taken at corresponding timepoints are shown in the lower panel. (TIF) [file pgen.1005353.s002.tif]

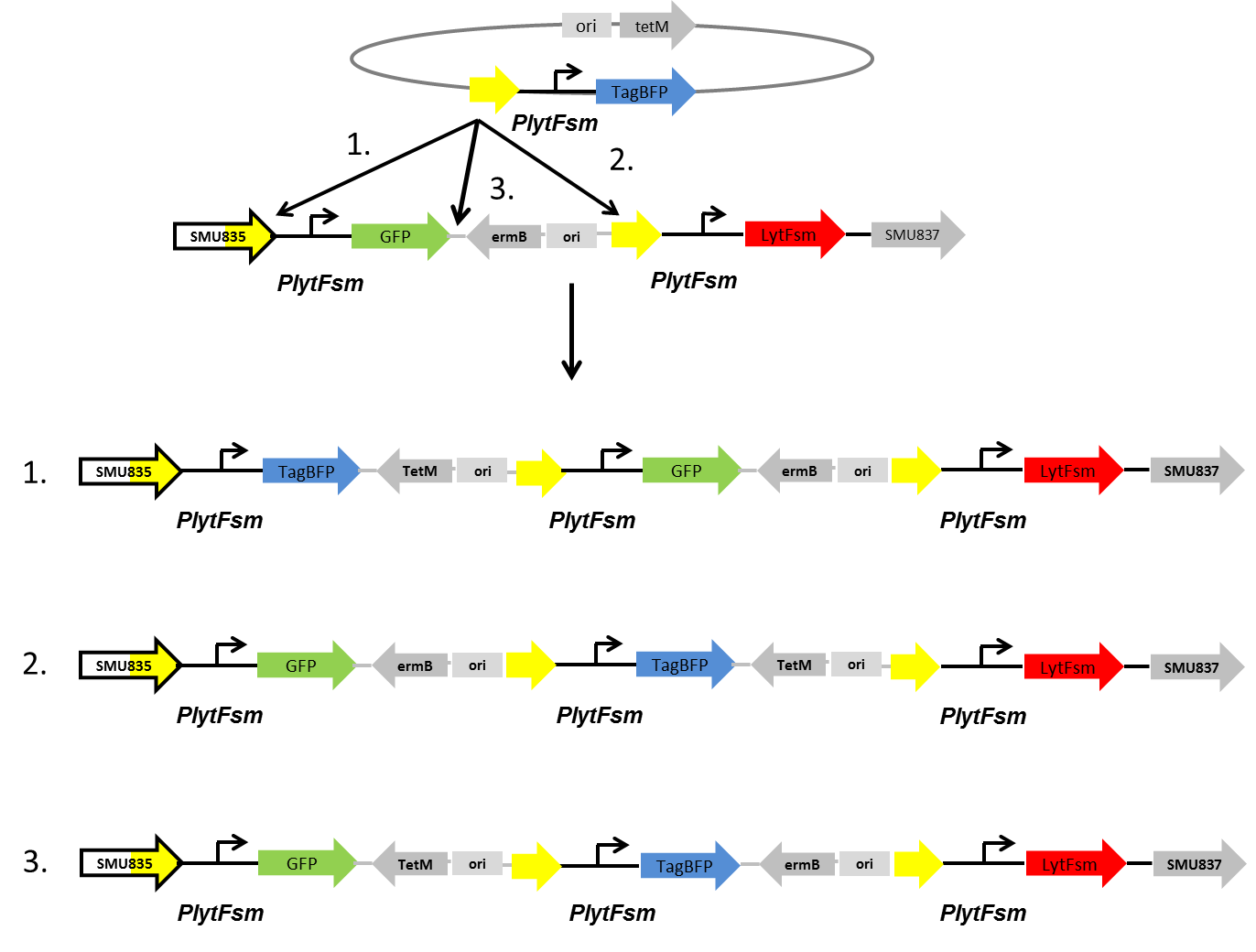

Supplement: S3 Fig — The reporter strain is constructed by a sequential double integration of the plasmids LytFsm pMR1 and LytFsm pMR2 into the same genomic locus. After chromosomal integration of the LytFsm pMR1 plasmid via single homologous recombination 3 different homologous sites are present in the genome allowing integration of the LytFsm pMR2 plasmid. Integration at the different sites (1–3) results in a slightly different genomic context for the resulting constructs. In all cases the second integration results in reporter strains carrying TagBFP2 and gfp+, each under the control of the LytFsm promoter. (TIF) [file pgen.1005353.s003.tif]

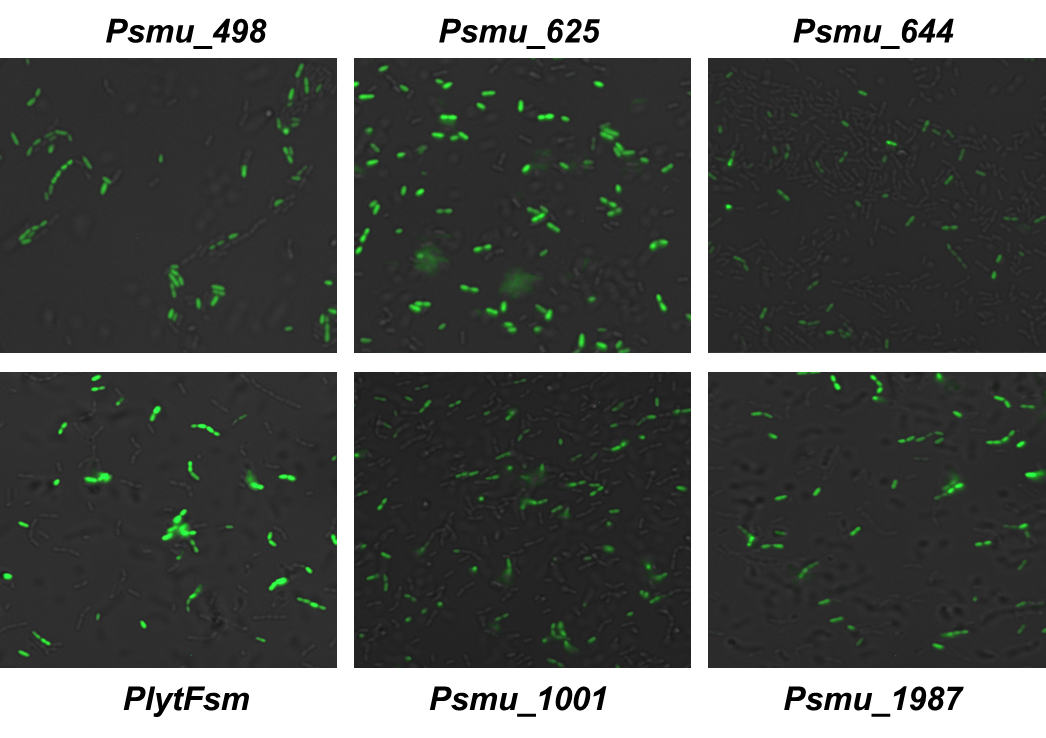

Supplement: S4 Fig — Microscopic overlay images of GFP+ reporter strains for late competence genes smu_498, smu_625, smu_644, lytFsm, smu_1001 and smu_1987 are shown. Images were taken 3 hours after induction of gene expression with CSP. (TIF) [file pgen.1005353.s004.tif]

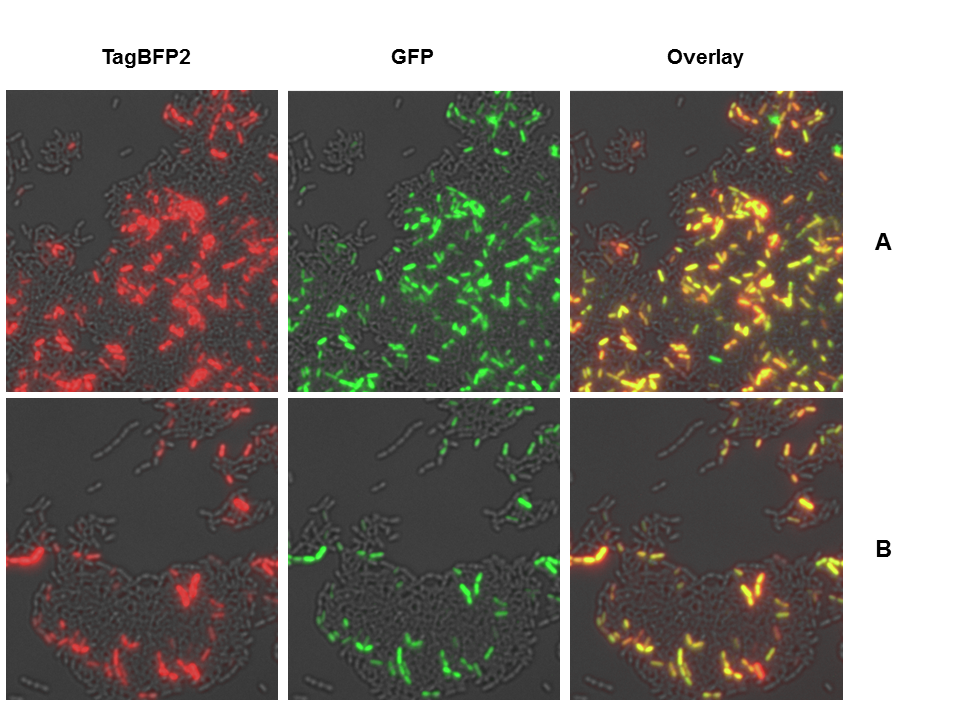

Supplement: S5 Fig — In (A) overlay images of a CSP-induced dual fluorescence reporter strain are shown which expresses TagBFP2 under the control of the comS-promoter and GFP+ under the control of the sigX-promoter. In the left column the TagBFP2 channel, in the middle column the GFP+ channel and in the right column the overlay images of the green and the blue channels are shown. In (B) overlay images of CSP induced (2μM) LytFsm pMR1 LytFsm pMR2 dual reporter strain are shown. The strain carries TagBFP2 and GFP+ both under control of the identical promoter (LytFsm). Images were taken 3 h after induction of gene expression by CSP. (TIF) [file pgen.1005353.s005.tif]

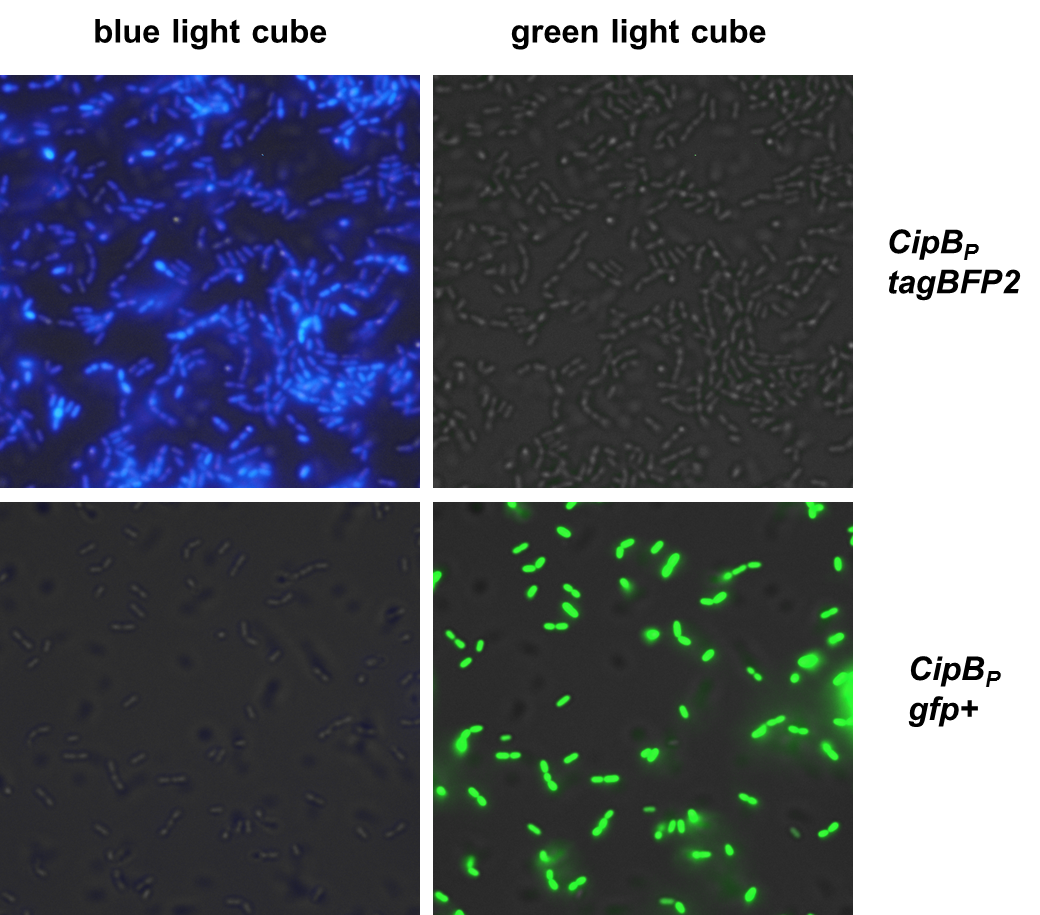

Supplement: S6 Fig — Two cipB reporter strains, one expressing TagBFP2 and the other GFP+ were analyzed using both the green and the blue light cube of an EvosR fluorescence microscope. Reporter strains were grown in THBY and induced with 2μM CSP. 3 h post CSP induction images were collected. Phase contrast and fluorescence overlay images are shown. No spectral crosstalk into the other channel was observed for both reporter strains. (TIF) [file pgen.1005353.s006.tif]

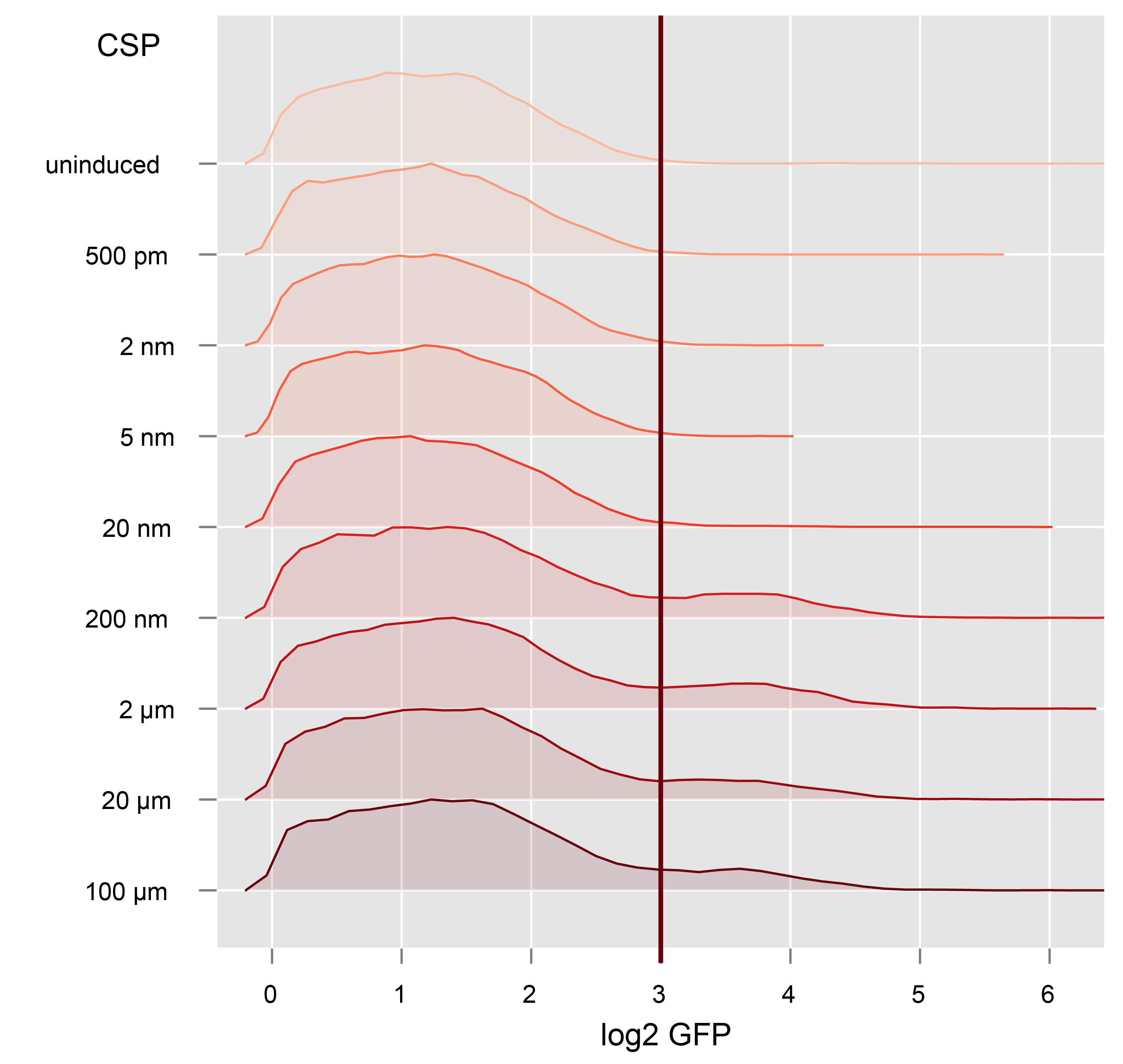

Supplement: S7 Fig — A LytFsm pAE03 reporter strain grown in THBY medium was induced with different concentration of CSP. 3h after induction samples were taken and analyzed using flow cytometry. 50000 individual cells were analyzed to determine the GFP intensity distribution of the population. (TIF) [file pgen.1005353.s007.tif]

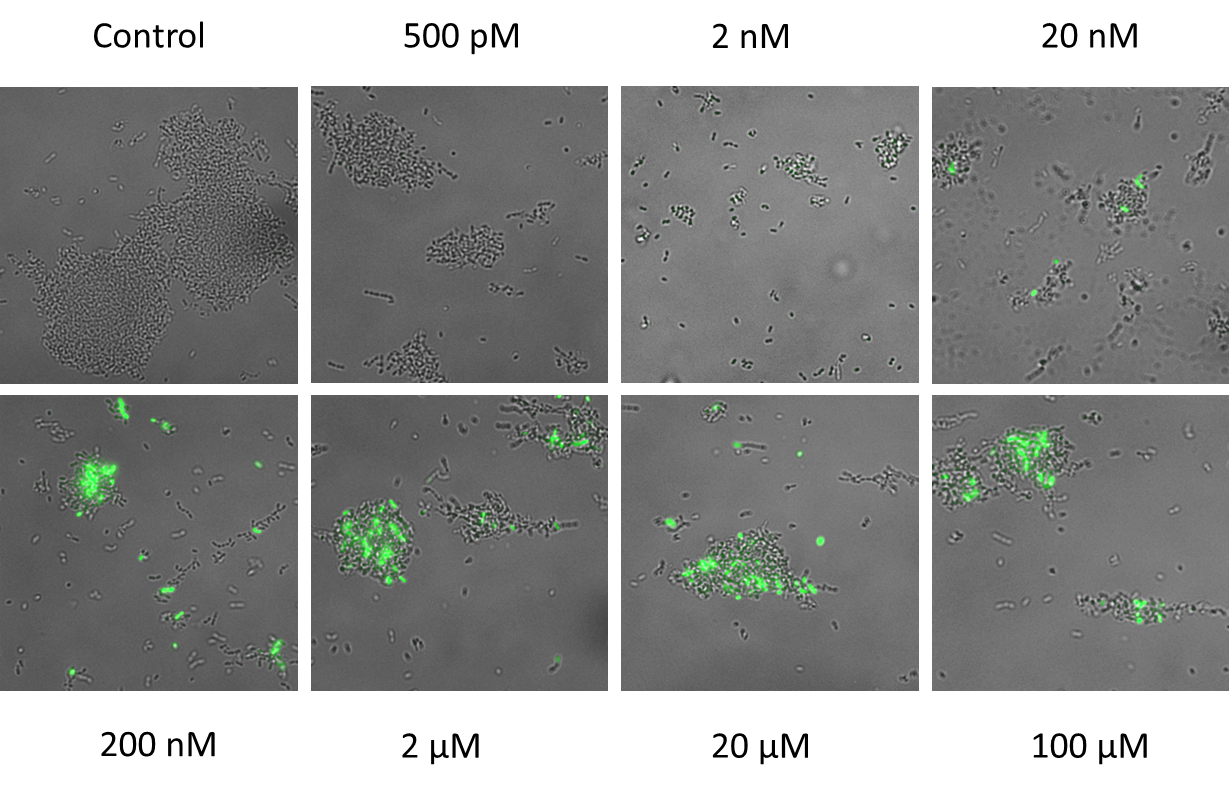

Supplement: S8 Fig — A LytFsm pAE03 reporter strain grown in THBY medium was induced with different concentration of CSP. 3h after induction samples were taken and the fluorescence was visualized under the fluorescence microscope. Overlay images of the GFP fluorescence and phase contrast are shown. (TIF) [file pgen.1005353.s008.tif]

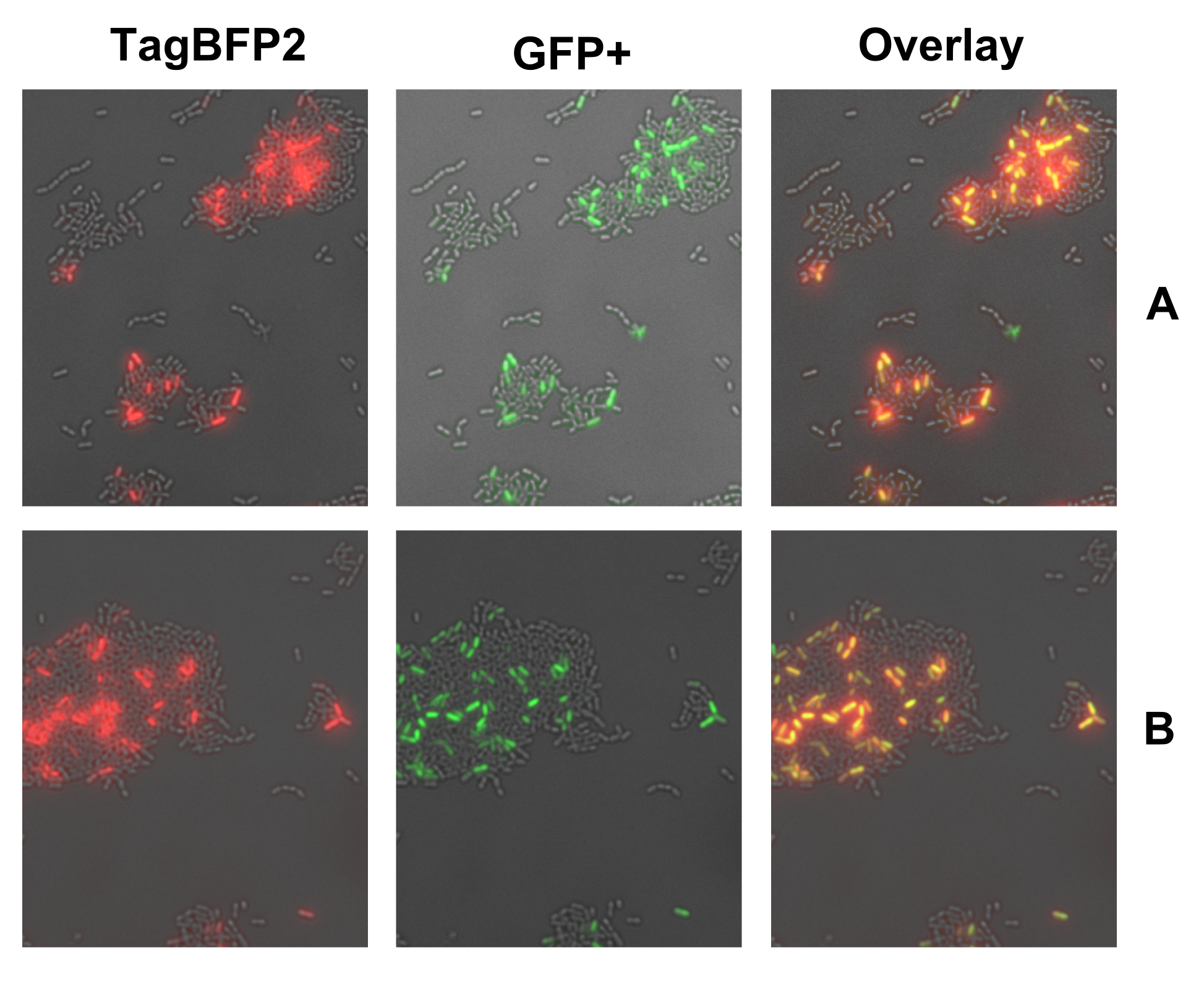

Supplement: S9 Fig — Single cell co-expression analysis of comE with the late competence gene lytFsm. In (A) overlay images of a dual fluorescence reporter strain are shown which expresses tagBFP2 under the control of the comE-promoter and GFP+ under the control of the lytFsm-promoter. In the left column the TagBFP2 channel, in the middle column the GFP+ channel and in the right column the overlay images of the green and the blue channels are shown. In (B) a dye swap of the dual reporter was performed, thus tagBFP2 expression is under the control of the lytFsm promoter while gfp+ expression is controlled by the promoter of comE. Images were taken 3 h after induction of gene expression by CSP. (PNG) [file pgen.1005353.s009.png]

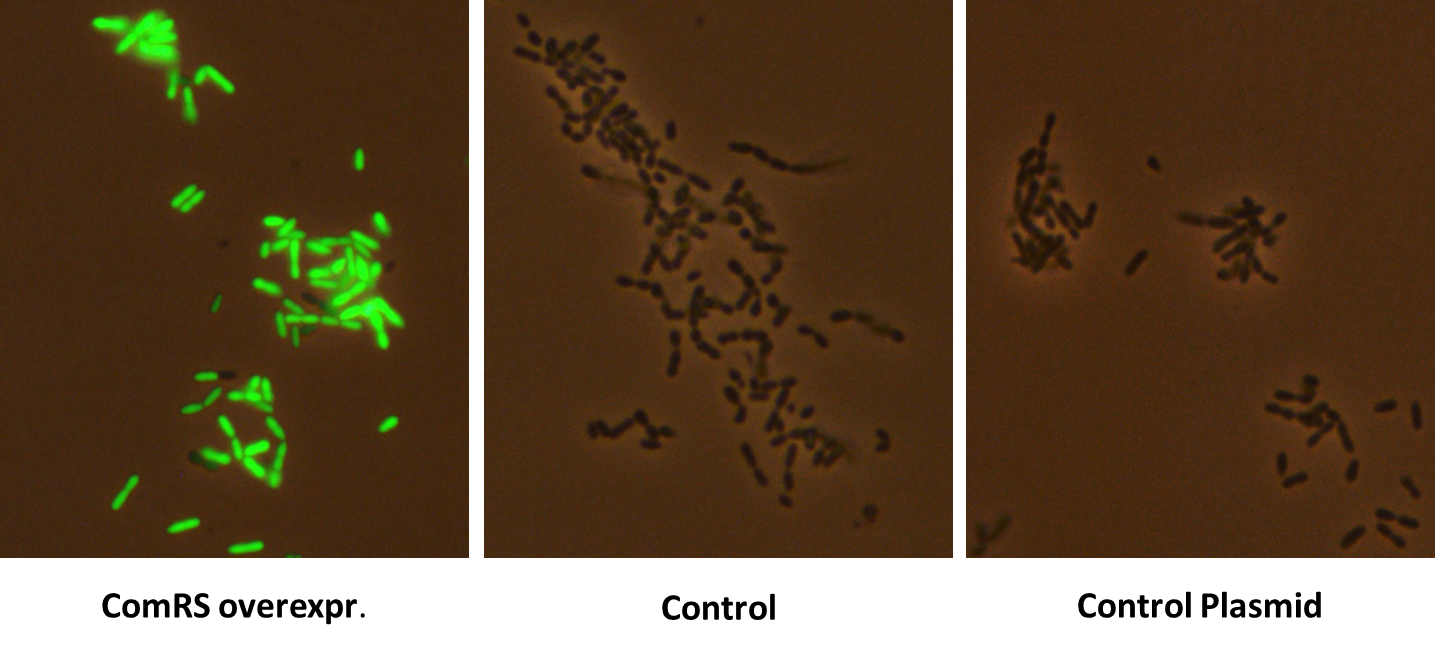

Supplement: S10 Fig — A LytFsm comRS (comRS overexpr.) reporter strain carrying the comRS genes under control of the strong constitutive P23 promoter was grown in chemically defined medium. As controls reporter strains expressing wild-type levels of comRS, LytFsm pAE03 (control) and LytFsm pIB166 (plasmid control) were cultivated under the same conditions. Cells were harvested after the cultures reached an OD600 = 0.2 and analyzed using fluorescence microscopy. The overlay images (gfp fluorescence/phase contrast) are shown. (TIF) [file pgen.1005353.s010.tif]

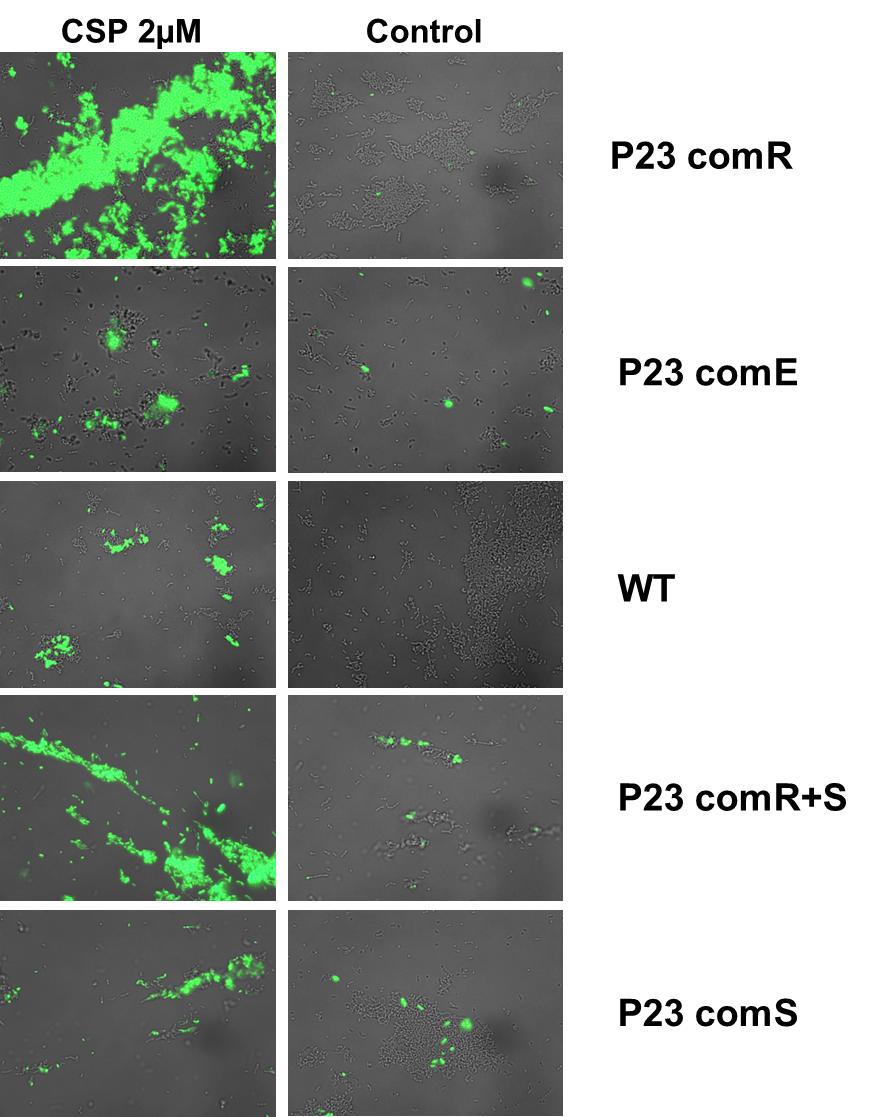

Supplement: S11 Fig — Expression of the different genes was under the control of the strong constitutive lactococcal P23 promoter on the replicative plasmid pIB166. LytFsm pAE03 derived overexpression strains were grown in THBY and induced with 2μM CSP. 3 h post CSP supplementation samples were taken and images were collected using fluorescence microscopy. In the left column overlay images (gfp/phase contrast) of the CSP induced strains are shown while in the right column the overlays of the un-induced strains are presented. (TIF) [file pgen.1005353.s011.tif]

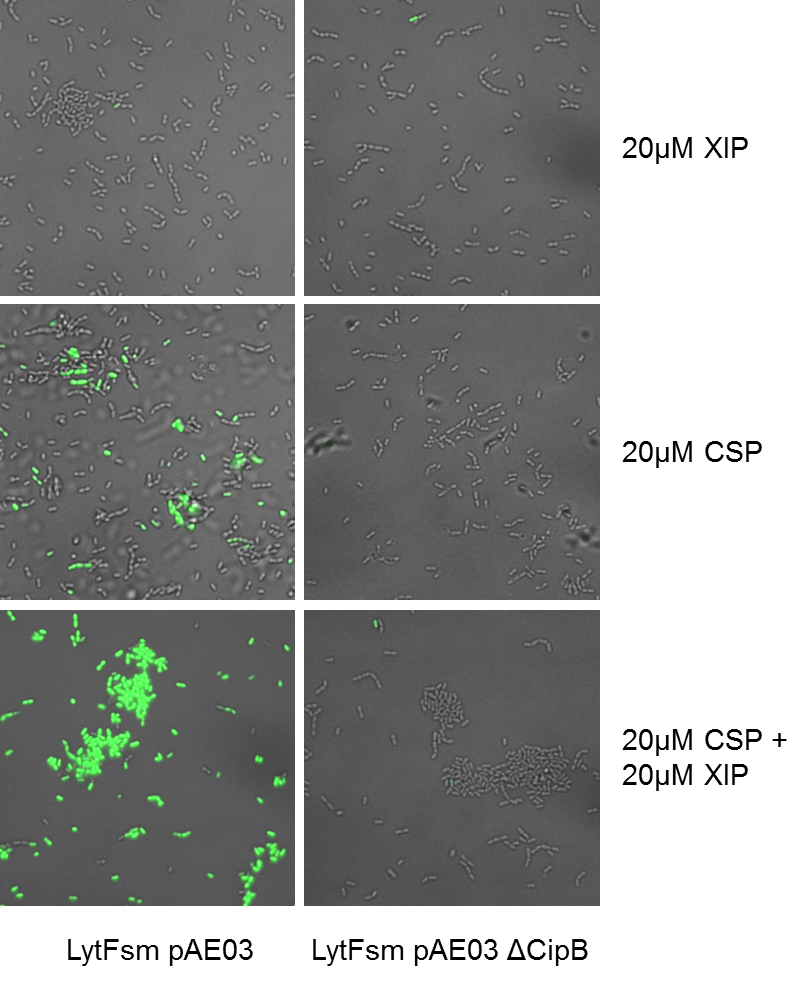

Supplement: S12 Fig — The LytFsm pAE03 and LytFsm pAE03 ΔCipB reporter strains were grown in complex medium until the culture reached an OD600 of 0.2. The culture was divided and induced with either 20 μM CSP or 20 μM XIP alone or a combination of 20 μM XIP and 20 μM CSP. 3 h post induction cells were harvested and analyzed using fluorescence microscopy. Overlay images (phase contrast and green fluorescence) of the collected images are shown. (TIF) [file pgen.1005353.s012.tif]

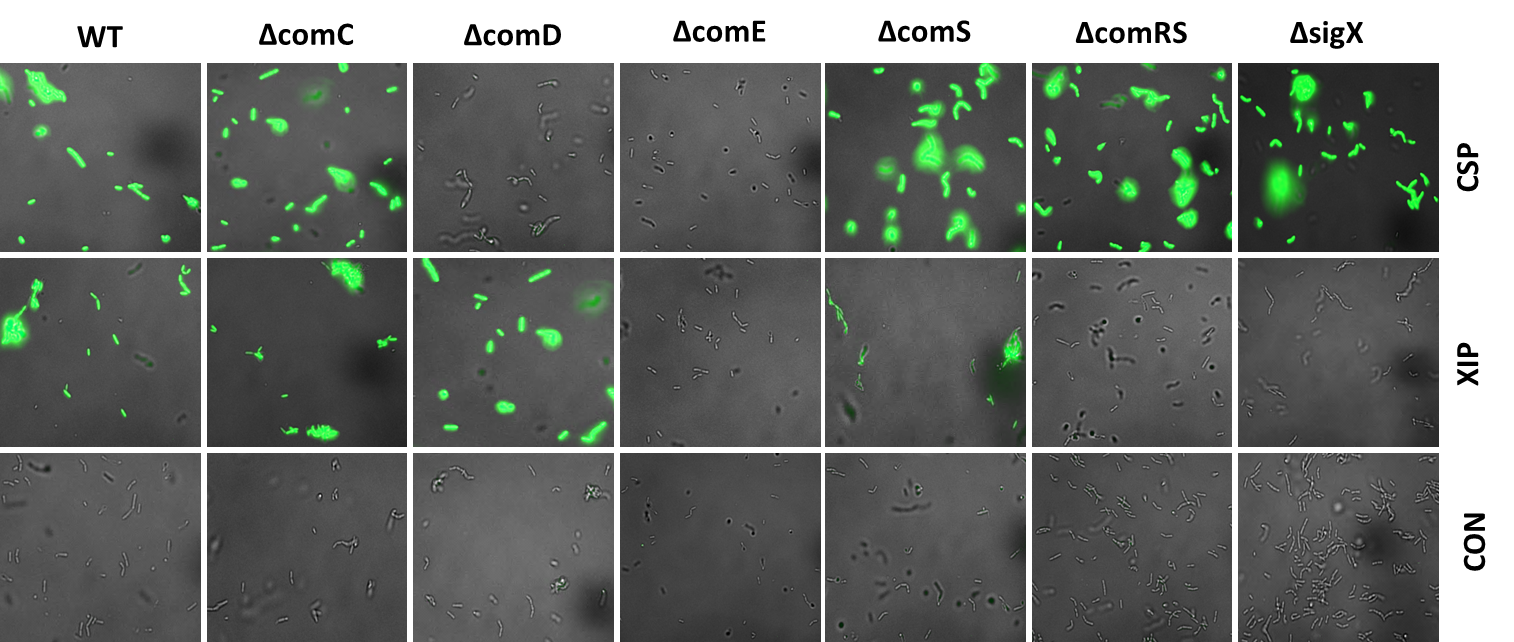

Supplement: S13 Fig — Overlay microscopic images were recorded 3 h after induction. Un-induced controls of the reporter strains are shown in the bottom row (CON). (TIF) [file pgen.1005353.s013.tif]

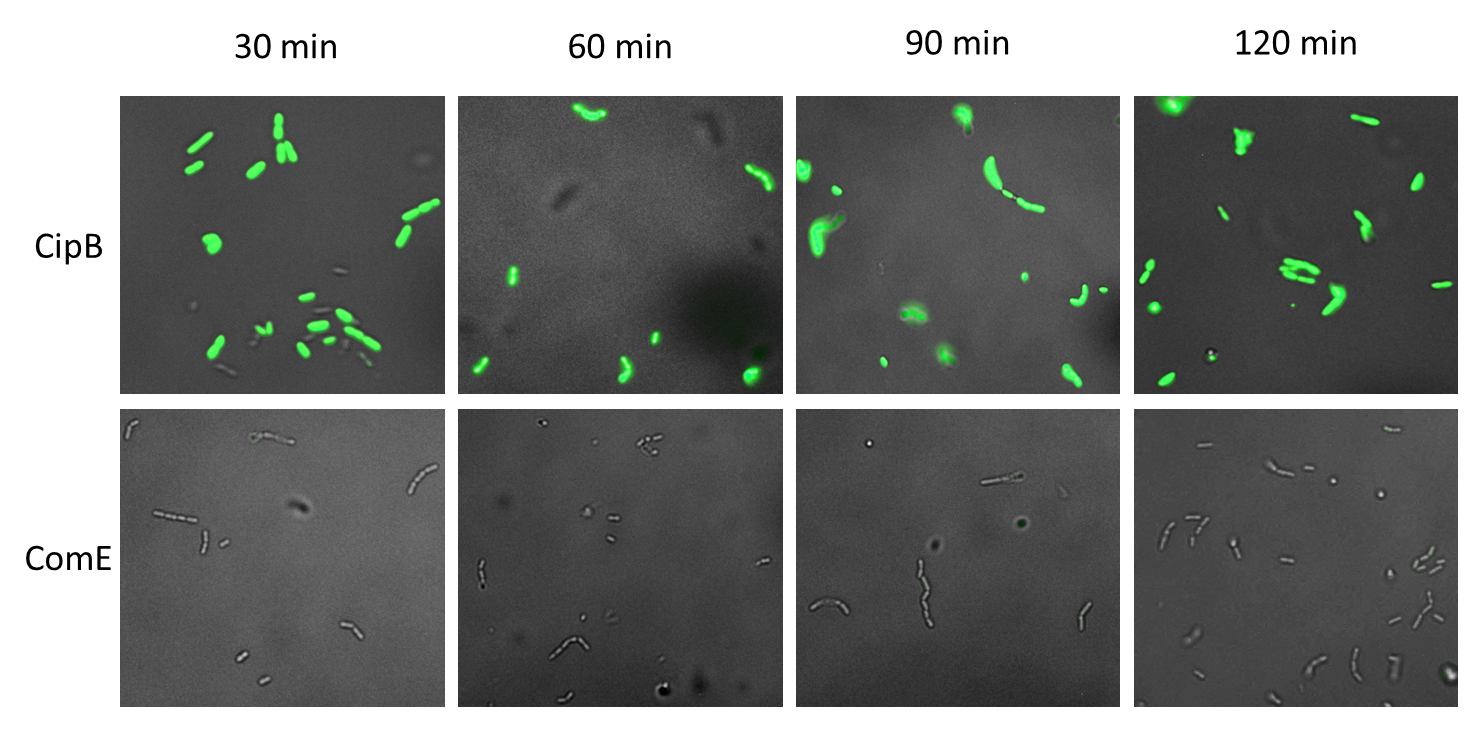

Supplement: S14 Fig — Fluorescent CipB pMR1 and ComE pMR1 reporter strains were grown in CDM under CSP induced (2μM) conditions. Fluorescent and phase-contrast images were collected 30, 60, 90 and 120 minutes post CSP addition. Overlay images are shown. (TIF) [file pgen.1005353.s014.tif]

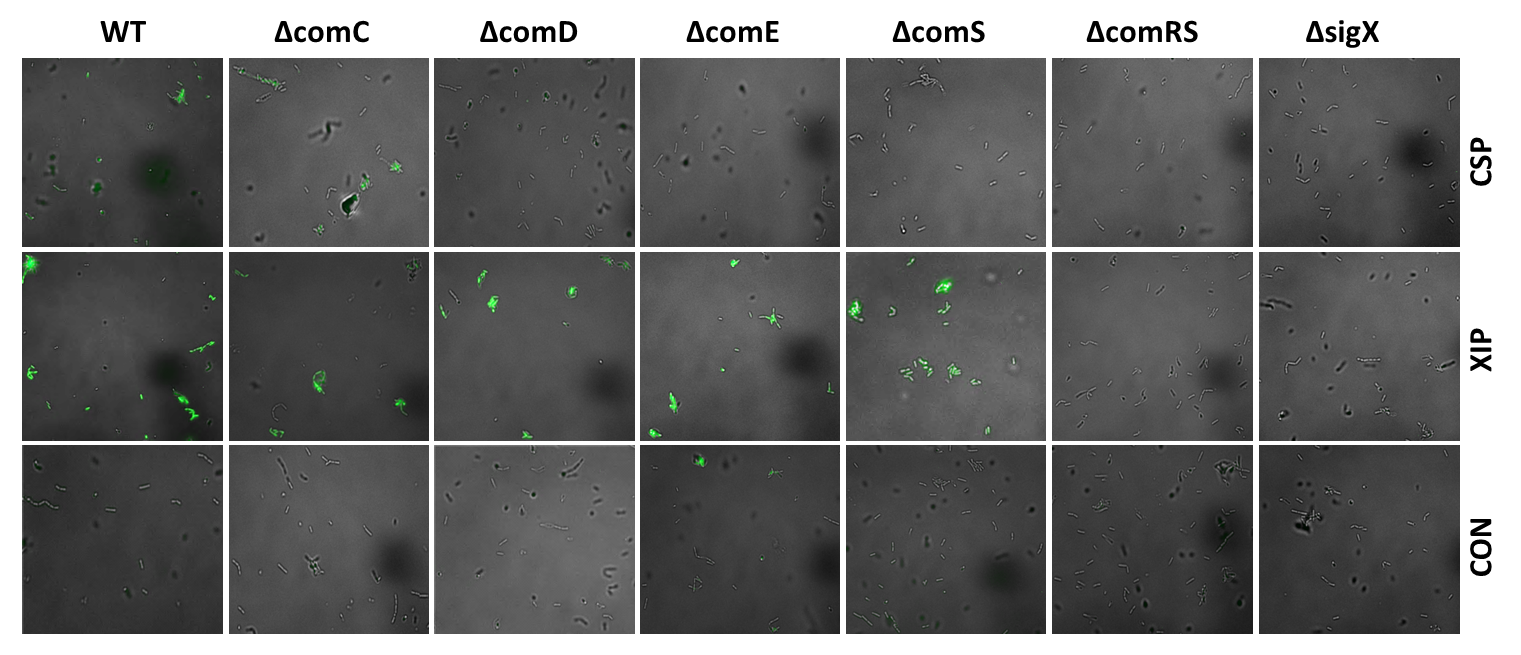

Supplement: S16 Fig — Overlay microscopic images were recorded 3 h after induction. Un-induced controls of the reporter strains are shown in the bottom row (CON). (TIF) [file pgen.1005353.s016.tif]

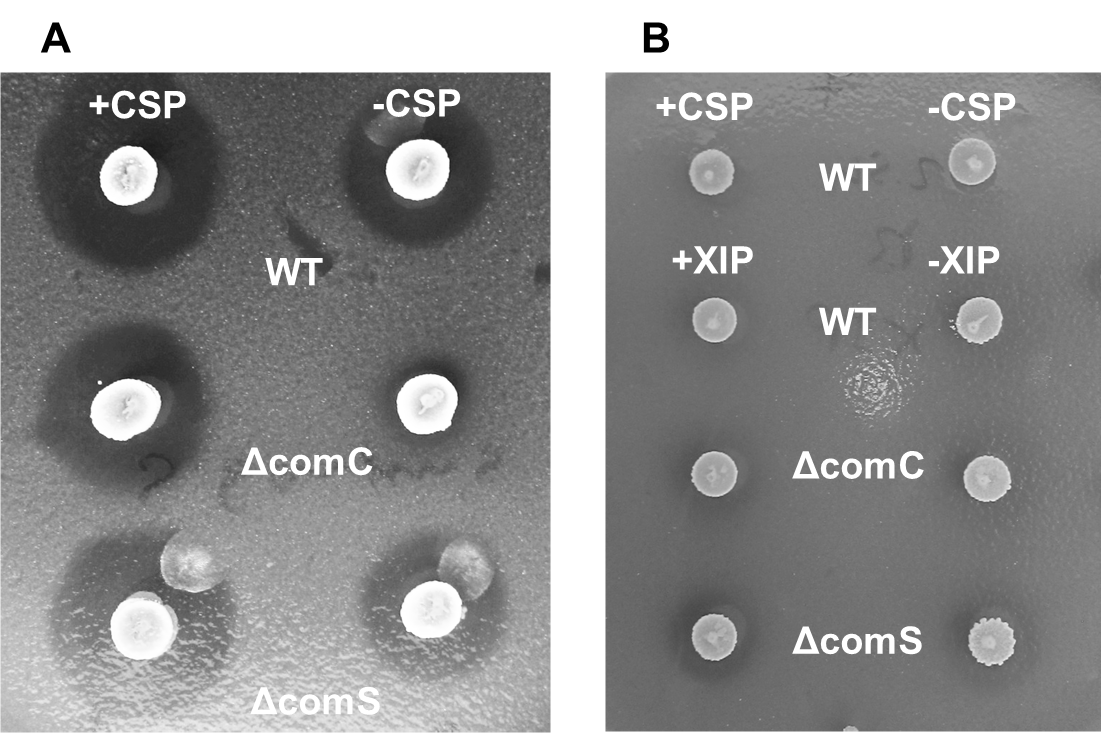

Supplement: S17 Fig — CSP (2 μM) or XIP (2 μM) induced S. mutans WT, ΔcomC and ΔcomS deletion strains were grown on THBY or CDM agar. Uninduced strains were used to detect self-induced expression of bacteriocins upon growth. After 24 h incubation of the spotted producer strains plates were overlaid with the L. lactis indicator strain diluted in either THBY top agar (A) or CDM top agar (B). In (A) an image of the overlaid THBY agar plate is shown. In the left column (+CSP) strains induced with 2 μM CSP are spotted while in the right column (-CSP) the corresponding uninduced strains are shown. (B) shows an image of the CDM agar plate, the left column contains the CSP or XIP (+CSP/+XIP) induced and spotted strains while the corresponding uninduced strains (-CSP/-XIP) were spotted in the right column. (TIF) [file pgen.1005353.s017.tif]

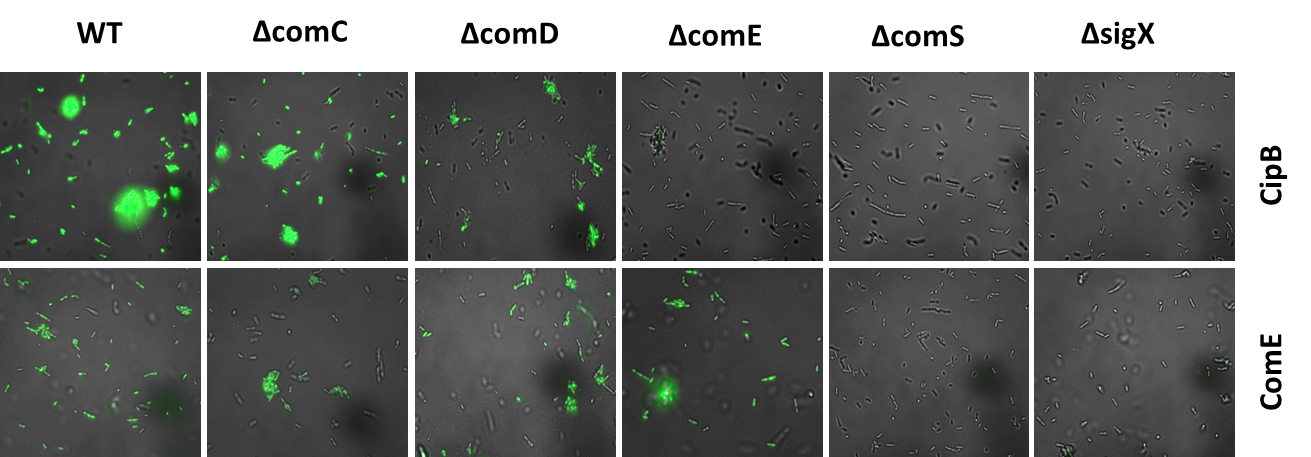

Supplement: S18 Fig — CipB pMR1 and comE pMR1 reporter strains in different gene deletion backgrounds were grown in CDM until the cultures reached the stationary growth phase (8h growth). Cells were harvested and fluorescence images collected using fluorescence microscopy. Overlay images (gfp fluorescence/phase contrast) of the different cipB pMR1 (upper panel) and comE pMR1 (lower panel) reporter strains are presented. (TIF) [file pgen.1005353.s018.tif]

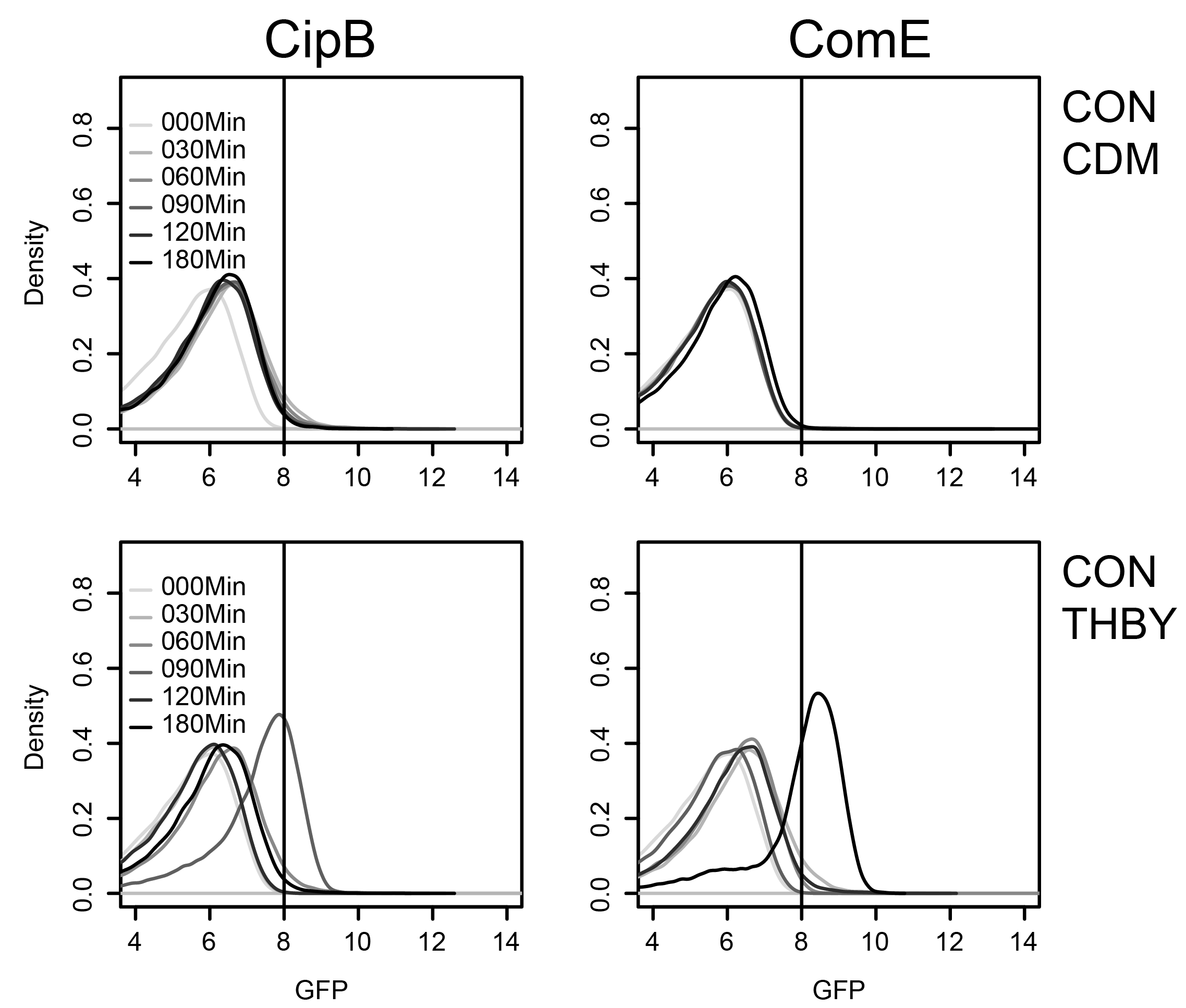

Supplement: S19 Fig — Uninduced CipB pAE03 (left column) and ComE pAE03 (right column) fluorescent reporter strains were grown in CDM (upper panel) and THBY (lower panel). At timepoints corresponding to 0, 30, 60, 90, 120 and 180 minutes post induction (see Fig 5) samples were taken and analysed using flow cytometry. The distribution of the gfp fluorescence for 50.000 analyzed cells is shown. (TIF) [file pgen.1005353.s019.tif]

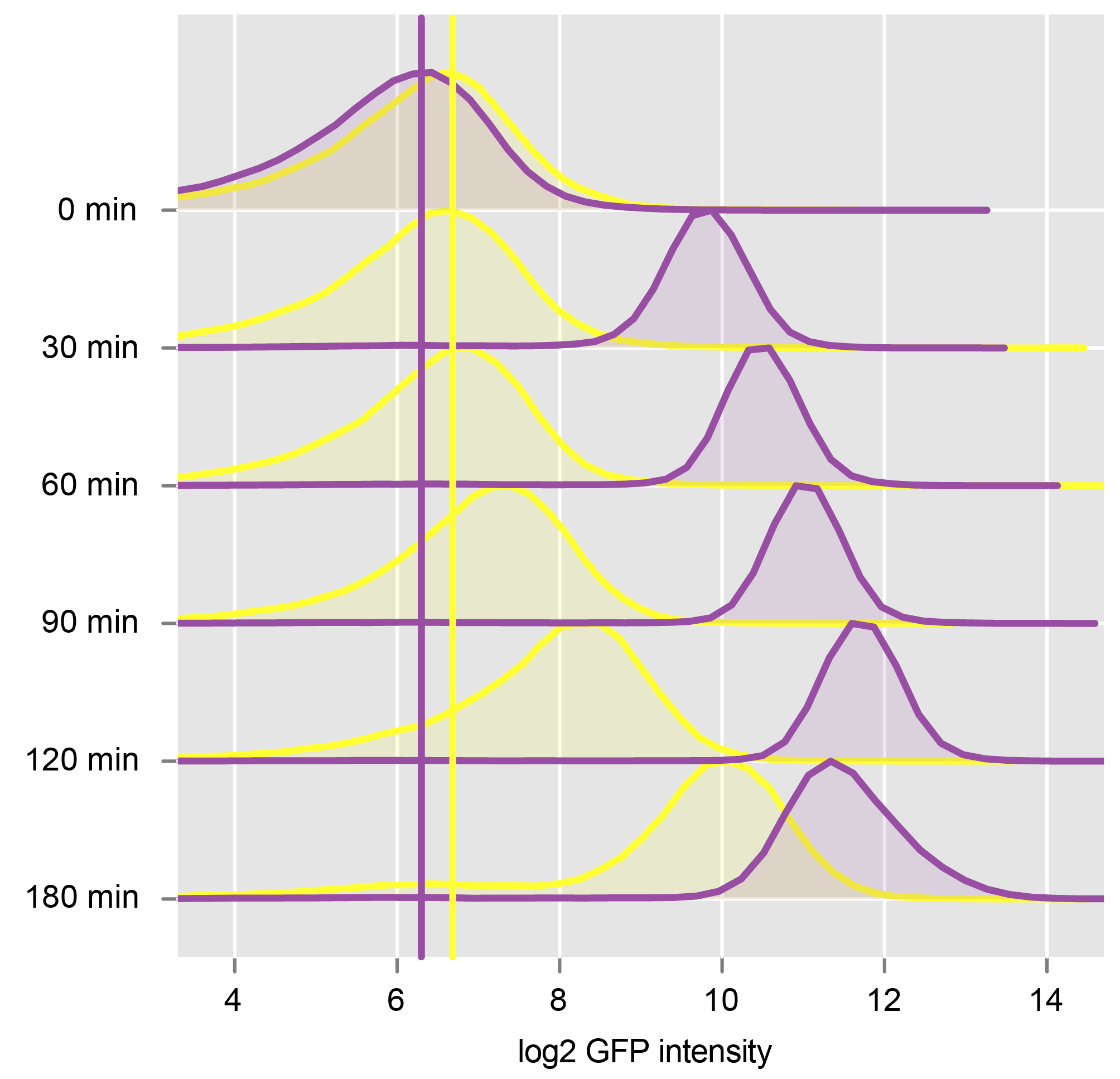

Supplement: S20 Fig — The CipB pAE03 reporter strain was either grown in THBY and induced with 2 μM CSP (violet plots) or grown in CDM and induced with 2 μM XIP (yellow plots). 0, 30, 60, 90, 120 and 180 minutes post induction samples were taken and analysed using flow cytometry. The gfp fluorescence of 50.000 cells was analyzed for each condition and the corresponding density plots (log2 gfp fluorescence intensity) are shown in the course of time. (TIF) [file pgen.1005353.s020.tif]
