# Supplementary material for: The Alternative Sigma Factor SigX Controls Bacteriocin Synthesis and Competence, the Two Quorum Sensing Regulated Traits in Streptococcus mutans
Source: PLoS Genet. 2015 Jul 9;11(7):e1005353. doi: 10.1371/journal.pgen.1005353 (PMC4497675; doi:10.1371/journal.pgen.1005353)
Supplement: S1 Table — (DOCX) [file pgen.1005353.s021.docx]

**Table S1. Strains used in this study**

| **Strain** | **Relevant genotype** | **Reference** |
| --- | --- | --- |
| *Streptococcus mutans* UA159 | Wild type, Erm^s^, Tet^s^, Cat^s^ | ATCC 700610 |
| **Single fluorescent reporter strains** | | |
| ComE pAE03 | UA159::ϕ(*comE_P_-gfp+*), Erm^r^ | This study |
| ComE pMR1 | UA159::ϕ(*comE_P_-gfp+*), Tet^r^ | This study |
| ComE pMR2 | UA159::ϕ(*comE_P_-tagbfp2*), Erm^r^ | This study |
| CipB pAE03 | UA159::ϕ(*cipB_P_-gfp+*), Erm^r^ | This study |
| CipB pMR1 | UA159::ϕ(*cipB_P_-gfp+*), Tet^r^ | This study |
| CipB pMR2 | UA159::ϕ(*cipB_P_-tagbfp2*), Erm^r^ | This study |
| ComS pAE03 | UA159::ϕ(*comS_P_-gfp+*), Erm^r^ | This study |
| ComS pMR1 | UA159::ϕ(*comS_P_-gfp+*), Tet^r^ | This study |
| ComS pMR2 | UA159::ϕ(*comS_P_-tagbfp2*), Erm^r^ | This study |
| ComX pAE03 | UA159::ϕ(*comX_P_-gfp+*), Erm^r^ | This study |
| ComX pMR1 | UA159::ϕ(*comX_P_-gfp+*), Tet^r^ | This study |
| ComX pMR2 | UA159::ϕ(*comX_P_-tagbfp2*), Erm^r^ | This study |
| LytFsm pAE03 | UA159::ϕ(*lytFsm_P_-gfp+*), Erm^r^ | This study |
| LytFsm pMR1 | UA159::ϕ(*lytFsm_P_-gfp+*), Tet^r^ | This study |
| LytFsm pMR2 | UA159::ϕ(*lytFsm_P_-tagbfp2*), Erm^r^ | This study |
| SMU_1001 pAE03 | UA159::ϕ(*smu_1001_P_-gfp+*), Erm^r^ | This study |
| SMU_498 pAE03 | UA159::ϕ(*smu_498_P_-gfp+*), Erm^r^ | This study |
| SMU_625 pAE03 | UA159::ϕ(*smu_625_P_-gfp+*), Erm^r^ | This study |
| SMU_644 pAE03 | UA159::ϕ(*smu_644_P_-gfp+*), Erm^r^ | This study |
| SM0_ 1987 pAE03 | UA159::ϕ(*smu_1987_P_-gfp+*), Erm^r^ | This study |
| SMU_1913 pAE03 | UA159::ϕ(*smu_1913_P_-gfp+*), Erm^r^ | This study |
| CipI pAE03 | UA159::ϕ(*cipI_P_-gfp+*), Erm^r^ | This study |
| CipB pMR3 | UA159::ϕ(*cipB_P_-mCherry*), Erm^r^ | This study |
| CipB pMR4 | UA159::ϕ(*cipB_P_-mCherry codon optimised*), Erm^r^ | This study |
| CipB pMR5 | UA159::ϕ(*cipB_P_-mCherry with 15 aa folding tag*), Erm^r^ | This study |
| CipB pMR6 | UA159::ϕ(*cipB_P_-turboRfp*), Erm^r^ | This study |
| CipB pMR7 | UA159::ϕ(*cipB_P_-tdTomato*), Erm^r^ | This study |
| **Dual fluorescent reporter strains** | | |
| LytFsm pMR1 ComE pMR2 | UA159::ϕ(*lytFsm_P_-gfp+,comE_P_-tagbfp2*), Tet^r^, Erm^r^ | This study |
| LytFsm pMR2 ComE pMR1 | UA159::ϕ(*lytFsm_P_-tagbfp2,comE_P_-gfp+*), Tet^r^ + Erm^r^ | This study |
| ComE pMR1 ComS pMR2 | UA159::ϕ(*comE_P_-gfp+,comS_P_-tagbfp2*), Tet^r^ + Erm^r^ | This study |
| ComE pMR2 ComS pMR1 | UA159::ϕ(*comE_P_-tagbfp2,comS_P_-gfp+*), Tet^r^ + Erm^r^ | This study |
| ComE pMR1 ComX pMR2 | UA159::ϕ(*comE_P_-gfp+,comX_P_-tagbfp2*), Tet^r^ + Erm^r^ | This study |
| ComE pMR2 ComX pMR1 | UA159::ϕ(*comE_P_-tagbfp2,comX_P_-gfp+*), Tet^r^ + Erm^r^ | This study |
| LytFsm pMR1 LytFsm pMR2 | UA159::ϕ(*lytFsm_P_-gfp+,LytFsm_P_-tagbfp2*), Tet^r^, Erm^r^ | This study |
| ComX pMR1 ComS pMR2 | UA159::ϕ(*comX_P_-gfp+,comS_P_-tagbfp2*), Tet^r^ + Erm^r^ | This study |
| ComX pMR2 ComS pMR1 | UA159::ϕ(*comX_P_-tagbfp2,comS_P_-gfp+*), Tet^r^ + Erm^r^ | This study |
| **Single fluorescent reporter strains in different gene deletion backgrounds** | | |
| ComE pMR1ΔcomC | UA159::ϕ(*comE_P_-gfp+*), *ΔcomC::erm*, Tet^r^ + Erm^r^ | This study |
| ComE pMR1ΔcomD | UA159::ϕ(*comE_P_-gfp+*), Δ*comD::erm*, Tet^r^ + Erm^r^ | This study |
| ComE pMR1ΔcomE | UA159::ϕ(*comE_P_-gfp+*), *ΔcomE::erm*, Tet^r^ + Erm^r^ | This study |
| ComE pMR1ΔcomS | UA159::ϕ(*comE_P_-gfp+*), Δ*comS::erm*, Tet^r^ + Erm^r^ | This study |
| ComE pMR1ΔcomRS | UA159::ϕ(*comE_P_-gfp+*), Δ*comRS::erm*, Tet^r^ + Erm^r^ | This study |
| ComE pMR1ΔcomX | UA159::ϕ(*comE_P_-gfp+*), Δ*comX::erm*, Tet^r^ + Erm^r^ | This study |
| CipB pMR1ΔcomC | UA159::ϕ(*cipB_P_-gfp+*), Δ*comC::erm*, Tet^r^ + Erm^r^ | This study |
| CipB pMR1ΔcomD | UA159::ϕ(*cipB_P_-gfp+*), *ΔcomD::erm*, Tet^r^ + Erm^r^ | This study |
| CipB pMR1ΔComE | UA159::ϕ(*cipB_P_-gfp+*), Δ*comE::erm*, Tet^r^ + Erm^r^ | This study |
| CipB pMR1ΔcomS | UA159::ϕ(*cipB_P_-gfp+*), Δ*comS::erm*, Tet^r^ + Erm^r^ | This study |
| CipB pMR1ΔcomRS | UA159::ϕ(*cipB_P_-gfp+*), Δ*comRS::erm*, Tet^r^ + Erm^r^ | This study |
| CipB pMR1ΔcomX | UA159::ϕ(*cipB_P_-gfp+*), *ΔcomX::erm*, Tet^r^ + Erm^r^ | This study |
| **Overexpression strains in LytFsm_GFP+ Background** | | |
| LytFsm pIB166 | UA159::ϕ(*lytFsm_P_-gfp+*), carrying pIB166, Erm^r^ + Cat^r^ | This study |
| LytFsm comE | UA159::ϕ(*lytFsm_P_-gfp+*), carrying pMR8, Erm^r^ + Cat^r^ | This study |
| LytFsm comR | UA159::ϕ(*lytFsm_P_-gfp+*), carrying pMR12, Erm^r^ + Cat^r^ | This study |
| LytFsm comS | UA159::ϕ(*lytFsm_P_-gfp+*), carrying pMR13, Erm^r^ + Cat^r^ | This study |
| LytFsm comRS | UA159::ϕ(*lytFsm_P_-gfp+*), carrying pMR14, Erm^r^ + Cat^r^ | This study |
| LytFsm comD | UA159::ϕ(*lytFsm_P_-gfp+*, Ω*smu_1342::P_23_-comD-cat*, Cat^r^ | This study |
| **Strains used for phosphomimetic analysis of ComE** | | |
| CipB pMR1ΔComE Enat | UA159::ϕ(*cipB_P_-gfp+*), Δ*comE::erm*, carrying pMR9 Tet^r^ + Erm^r^ + Cat^r^ | This study |
| CipB pMR1ΔComE E | UA159::ϕ(*cipB_P_-gfp+*), *ΔcomE::erm*, carrying pMR8 Tet^r^ + Erm^r^ + Cat^r^ | This study |
| CipB pMR1ΔComE D60E | UA159::ϕ(*cipB_P_-gfp+*), Δ*comE::erm*, carrying pMR10 Tet^r^ + Erm^r^ + Cat^r^ | This study |
| CipB pMR1ΔComE D60A | UA159::ϕ(*cipB_P_-gfp+*), *ΔcomE::erm*, carrying pMR11 Tet^r^ + Erm^r^ + Cat^r^ | This study |
