# Supplementary material for: The Alternative Sigma Factor SigX Controls Bacteriocin Synthesis and Competence, the Two Quorum Sensing Regulated Traits in Streptococcus mutans
Source: PLoS Genet. 2015 Jul 9;11(7):e1005353. doi: 10.1371/journal.pgen.1005353 (PMC4497675; doi:10.1371/journal.pgen.1005353)
Supplement: S2 Table — (DOCX) [file pgen.1005353.s022.docx]

**Table S2: Plasmids used in this study**

| **plasmid** | **relevant genotype** | **reference** |
| --- | --- | --- |
| pAE03 | promoterless *gfp+-ermB*, Erm^r^ | Eberhardt et al**.** |
| pJWV25 | *CzcD_P_-gfp+-tetM*, Tet^R^ | Eberhardt et al. |
| pMR1 | pAE03 removal of *ermB* ϕ(*tetM*), Tet^r^ | This study |
| pMR2 | pAE03 removal of *gfp+*, ϕ(*tagbfp2*), Erm^r^ | This study |
| pMR3 | pAE03 removal of *gfp+*, ϕ(*mCherry*), Erm^r^ | This study |
| pMR4 | pAE03 removal of *gfp+*, ϕ(*mCherry_opt*), Erm^r^ | This study |
| pMR5 | pAE03 removal of *gfp+*, ϕ(*mCherry_FT15*), Erm^r^ | This study |
| pMR6 | pAE03 removal of *gfp+*, ϕ(*turboRFP*), Erm^r^ | This study |
| pMR7 | pAE03 removal of *gfp+*, ϕ(*tdTomato*), Erm^r^ | This study |
| **Overexpression plasmids** | | |
| pIB166 | P_23_ of *Lactococcus lactis, cat*, Cat^r^ | Biswas et al. |
| pMR8 | pIB166 + ϕ(*comE*), Cat^r^ | This study |
| pMR9 | pIB166 + ϕ(*comE_p_-comE*), Cat^r^ | This study |
| pMR10 | pIB166 + ϕ(*comE_p_-comE* *D60E*), Cat^r^ | This study |
| pMR11 | pIB166 + ϕ(*comE_p_-comE D60A*), Cat^r^ | This study |
| pMR12 | pIB166 + ϕ(*comS*), Cat^r^ | This study |
| pMR13 | pIB166 + ϕ(*comR*), Cat^r^ | This study |
| pMR14 | pIB166 + ϕ(*comR+ comS*), Cat^r^ | This study |
| **Reporter plasmids** | | |
| PcomE pAE03 | pAE03 + ϕ(*comE_p_*) , Erm^r^ | This study |
| PcomS pAE03 | pAE03 + ϕ (*comS_p_*), Erm^r^ | This study |
| PcomX pAE03 | pAE03 + ϕ (*comX_p_*), Erm^r^ | This study |
| PCipB pAE03 | pAE03 + ϕ(*cipB_p_*), Erm^r^ | This study |
| PLytFsm pAE03 | pAE03 + ϕ(*lytFsm_p_*), Erm^r^ | This study |
| PSmu 498 pAE03 | pAE03 + ϕ (*smu_498_p_*), Erm^r^ | This study |
| PSmu 625 pAE03 | pAE03 + ϕ (*smu_625_p_*), Erm^r^ | This study |
| PSmu 644 pAE03 | pAE03 + ϕ (*smu_644_p_*), Erm^r^ | This study |
| PSmu 1001 pAE03 | pAE03 + ϕ (*smu_1001_p_*), Erm^r^ | This study |
| PSmu 1987 pAE03 | pAE03 + ϕ (*smu_1987_p_*), Erm^r^ | This study |
| PMutacin IV pAE03 | pAE03 + ϕ (*mutacinIV*_p_), Erm^r^ | This study |
| PMutacin VI pAE03 | pAE03 + ϕ (*mutacinVI_p_*), Erm^r^ | This study |
| PCipI pAE03 | pAE03 + ϕ (*cipI_p_*), Erm^r^ | This study |
| PSMU_1913 pAE03 | pAE03 + ϕ(*smu_1913_p_*), Erm^r^ | This study |
| PcomE pMR1 | pMR1 + ϕ (*comE_p_*), Tet^r^ | This study |
| PcomS pMR1 | pMR1 + ϕ (*comS_p_*), Tet^r^ | This study |
| PcomX pMR1 | pMR1 + ϕ (*comX_p_*), Tet^r^ | This study |
| PCipB pMR1 | pMR1 + ϕ(*cipB_p_*) , Tet^r^ | This study |
| PLytFsm pMR1 | pMR1 + ϕ(*lytFsm_p_*), Tet^r^ | This study |
| PcomE pMR2 | pMR2 + ϕ (*comE_p_*), Erm^r^ | This study |
| PcomS pMR2 | pMR2 + ϕ(*comS_p_*), Erm^r^ | This study |
| PcomX pMR2 | pMR2 + ϕ (*comX_p_*), Erm^r^ | This study |
| PCipB pMR2 | pMR2 + ϕ (*cipB_p_*) , Erm^r^ | This study |
| PLytFsm pMR2 | pMR2 + ϕ(*lytFsm_p_*) , Erm^r^ | This study |
