# Supplementary material for: The Alternative Sigma Factor SigX Controls Bacteriocin Synthesis and Competence, the Two Quorum Sensing Regulated Traits in Streptococcus mutans
Source: PLoS Genet. 2015 Jul 9;11(7):e1005353. doi: 10.1371/journal.pgen.1005353 (PMC4497675; doi:10.1371/journal.pgen.1005353)
Supplement: S3 Table — (DOCX) [file pgen.1005353.s023.docx]

**Table S3: Primers used in this study**

| **Primer** | **Sequence** | **Utilisation** |
| --- | --- | --- |
| P1AE03 | AAGGAGAAGAACTTTTCACTGGAGTTGTC | Amplification pAE03 |
| P2AE03 | GCGGCCGCGAATTCGAGCTCGGTAC | Amplification pAE03 |
| P3AE03 | CCTAAATTTCCCACAATCACTCACTTCT | Amplification pAE03 Exchange *ermB* vs. *tetM* |
| P4AE03 | CTGACAGCTTCCAAGGAGCTAAAGAGGT | Amplification pAE03 Exchange *ermB* vs. *tetM* |
| P5AE03 | GCTAGCGCGGCCGCGAATTCGAGCTCGGTA | Amplification pAE03 Exchange *gfp+* vs. *tagbfp2* |
| P6AE03 | TAAGGCCGGCCAATAAAGATCCTAGGACG | Amplification pAE03 Exchange *gfp+* vs. *tagbfp2* |
| TETM_F | TTGTGGGAAATTTAGGGCTAGCCAAGTAATCGTGAATGTCGCTGTG | Amplification *tetM* resitance cassette |
| TETM_R | CCTTGGAAGCTGTCAGTAGTATCCACTCGTATTCGTAGTCTTGTTTTATTTCC | Amplification *tetM* resitance cassette |
| TagBFP2_F | TCGCGGCCGCGCTAGCATGGTGTCTAAAGGTGAGGAATTAATCAAAGAG | Amplification *tagbfp2* |
| TagBFP2_R | TCTTTATTGGCCGGCCCTAATTCAGTTTATGGCCTAATTTAGACGGTAA | Amplification *tagbfp2* |
| P1MR2 | GCGGCCGCGAATTCGAGCTCGGTACC | Amplification vector pMR2 |
| P2MR2 | GTGTCTAAAGGTGAGGAATTAATCAAAGAGAA | Amplification vector pMR2 |
| mCherry_F | TCGCGGCCGCGCTAGCGTGAGCAAGGGCGAGGAGGATAACATGGC | Amplification *mCherry* |
| mCherry_R | TTATTGGCCGGCCTTACTACTTGTACAGCTCGTCCATGCCG | Amplification *mCherry* |
| mCherry_opt_F | TCGCGGCCGCGCTAGCATGGTTTCAAAGGGAGAAGAGGACAATATGG | Amplification codon optimized *mCherry* |
| mCherry_opt_R | TCTTTATTGGCCGGCCTTATTTGTACAATTCATCCATCCCACCTG | Amplification codon optimized *mCherry* |
| TurboRFP_F | TCGCGGCCGCGCTAGCATGTCAGAATTGATTAAGGAGAATATGCATATG | Ampflification *turboRFP* |
| Turbo_RFP_R | TCTTTATTGGCCGGCCTTAACGATGGCCTAATTTAGACGGTAAATCGCA | Amplification *turboRFP* |
| tdTomato_F | TCGCGGCCGCGCTAGCATGGTTAGTAAAGGTGAAGAGTTATTTACAGGT | Amplification tdTomato |
| tdTomato_R | TCTTTATTGGCCGGCCTTATTTGTATAATTCGTCCATACCTGGTGTAA | Amplification tdTomato |
| P2_MR4 | GTTTCAAAGGGAGAAGAGGACAATATGGCCAT | Amplification vector pMR4 |
| P2_MR3 | GTGAGCAAGGGCGAGGAGGATAACATGGC | Amplification vector pMR3+pMR5 |
| P2_MR6 | TCAGAATTGATTAAGGAGAATATGCATATGA | Amplification vector pMR6 |
| P2_MR7 | GTTAGTAAAGGTGAAGAGTTATTTACAGGTGTTG | Amplification vector pMR7 |
| PComSAE03For | TCGAATTCGCGGCCGCGCTAGCGAAGCTAAAAAGAGCTATGATGCTG | Ampflification Promoter ComS-pAE03/pMR1/2 |
| PcomSAE03Rev | AAAGTTCTTCTCCTTTGCTAGCTGAAAACATCCTGTTATTCTCCTTTCT | Ampflification Promoter ComS-pAE03/pMR1 |
| PcomSMR2Rev | CCTCACCTTTAGACACTGAAAACATCCTGTTATTCTCCTTTCTTTTTG | Ampflification Promoter ComS-pMR2 |
| PSigXAE03For | TCGAATTCGCGGCCGCGCTAGCGGTTCGAGTCCTACTGCCGGAGTTTATAT | Ampflification Promoter SigX pAE03/pMR1 |
| PSigXAE03Rev | AAAGTTCTTCTCCTTTTTCTTCCATCTATTACGATGACCTCCTT | Ampflification Promoter SigX-pAE03/pMR1 |
| PSigXMR2Rev | CCTCACCTTTAGACACCATTTCTTCCATCTATTACGATGACCTCCTTTTA | Ampflification Promoter SigX-pMR2 |
|  |  |  |
| **Primer** | **Sequence** | **Utilisation** |
| PcomEAE03For | TCGAATTCGCGGCCGCGCTAGCTTTTATAGCTGCTTTATCTTGGACAGTG | Ampflification Promoter ComE-PAE0/pMR1/2 |
| PcomEAE03Rev | AAAGTTCTTCTCCTTTAGAAATCATTATTTCTCCTTTAATCTTCT | Ampflification Promoter ComE-pAE03/pMR1 |
| PcomEMR2Rev | CCTCACCTTTAGACACCATAGAAATCATTATTTCTCCTTTAATCTTCTAT | Ampflification Promoter ComE-pMR2 |
| PCipBAE03For | TCGAATTCGCGGCCGCGCTAGCTCTGTTAAACAGCCGGAAAAATGTTGA | Ampflification Promoter CipB pAE03/pMR1/2 |
| PCipBAE03Rev | AAAGTTCTTCTCCTTTTGTATTCATATGATAAATACCCCTTCC | Ampflification Promoter CipB-pAE03/pMR1 |
| PCipBMR2Rev | CCTCACCTTTAGACACCATTGTATTCATATGATAAATACCCCTTCCCCA | Ampflification Promoter CipB-pMR2 |
| PLytFsmAE03For | TCGCGGCCGCGCTAGCAGTCTGTTTTCAAGATGGATTGACCCAAATACG | Ampflification Promoter LytFsm-pAE03/pMR1/2 |
| PLytFsmAE03Rev | AAAGTTCTTCTCCTTTTTTTCTCATTCTCAATCGAAATCTCCTTTATTC | Ampflification Promoter SMU_836(LytFsm)-pAE03 |
| PLytFsmMR2For | CCTCACCTTTAGACACCATTTTTCTCATTCTCAATCGAAATCTCCTTTAT | Ampflification Promoter LytFsm-pMR2 |
| P644AE03For | AAAGTTCTTCTCCTTTAACTAACATCAAAAATTCCTTTCTAAATAG | Ampflification Promoter SMU_644-pAE03/pMR3 |
| P644AE03Rev | TCGAATTCGCGGCCGCGCTAGCATCTTATATGAACTTCATATGTTTGCGT | Ampflification Promoter SMU_644-pAE03 |
| P644MR2Rev | CCTCACCTTTAGACACCATAACTAACATCAAAAATTCCTTTCTAAATAG | Ampflification Promoter SMU_644-pMR2 |
| P150AE03For | TCGAATTCGCGGCCGCGCTAGCTTTGATAAGCATGCGAACTTAAAATACAAATATGG | Ampflification Promoter SMU_150(Mutacin IV)-pAE03/pMR2 |
| P150AE03Rev | AAAGTTCTTCTCCTTTTGTATCCATATGATAAACACCCCTTTTTCA | Ampflification Promoter SMU_150(Mutacin IV)-pAE03 |
| P150MR2Rev | CCTCACCTTTAGACACCATTGTATCCATATGATAAACACCCCTTTTTCA | Ampflification Promoter SMU_150(Mutacin IV)-pMR2 |
|  |  |  |
|  |  |  |
| **Primer** | **Sequence** | **Utilisation** |
| P925AE03_F | TCGAATTCGCGGCCGCGCTAGCACTGTTGTCATCACTGTATCAGTTGATTTG | Ampflification Promoter SMU_925(CipI)-pAE03/pMR3 |
| P925AE03_R | AAAGTTCTTCTCCTTTATACAGCATTAGGCTTTCCTTATTAATAAC | Ampflification Promoter SMU_925(CipI)-pAE03 |
| P1001AE03_F | TCGCGGCCGCGCTAGCATCAGAAGATGGAAATAAATAGTATGACAAGC | Ampflification Promoter SMU_1001-pAE03 |
| P1001AE03_R | AAAGTTCTTCTCCTTTATTATCCATTTTTATACCTCACTATCTAAAC | Ampflification Promoter SMU_1001-pAE03 |
| P498AE03_F | TCGAATTCGCGGCCGCGCTAGCCTTCAACGATCCTAAAGCTTTGAGCG | Ampflification Promoter SMU_498-pAE03 |
| P498AE_R | AAAGTTCTTCTCCTTTATTCTCCATCACCTTTCTTATTCGTAAAC | Ampflification Promoter SMU_498-pAE03 |
| P625AE03_F | TCGCGGCCGCGCTAGCGACCAAGCTGGTGGAGCTTGATTTATCGTTTGC | Ampflification Promoter SMU_625-pAE03 |
| P625AE_R | AAAGTTCTTCTCCTTTGTCAACCATTTTAACCTCCTTACTTCTTTATTCG | Ampflification Promoter SMU_625-pAE03 |
| P1913AE_F | TCGAATTCGCGGCCGCGCTAGCAATTGTGCAGCAGGTATTGCTCTAGG | Ampflification Promoter SMU_1913-pAE03 |
| P1913AE_R | AAAGTTCTTCTCCTTTTTTAAACATTTCTTTTCTCCTTTATTCT | Ampflification Promoter SMU_1913-pAE03 |
| P1987AE03_F | TCGCGGCCGCGCTAGCGTGTTATCAAAAAGAGTGGGCAAAATTACG | Ampflification Promoter SMU_1987-pAE03 |
| P1987AE03_R | AAAGTTCTTCTCCTTTTTGAACCATAAAACCTCCCATCTTATCTATTCG | Ampflification Promoter SMU_1987-pAE03 |
| PCipB_MR4_R | CTTCTCCCTTTGAAACTGTATTCATATGATAAATACCCCTTCCCCAT | Amplification promoter CipB for construction of CipB pMR4 |
| PCipB_MR3_R | CCTCGCCCTTGCTCACTGTATTCATATGATAAATACCCCTTCCCCAT | Amplification promoter CipB for construction of CipB pMR3 |
| PCipB_MR5_R | CTCGCCCTTGCTCACTTCATTATCCATTACGTTAAATTGTTC | Amplification promoter CipB for construction of CipB pMR5 |
| PCipB_MR6_R | CCTTAATCAATTCTGATGTATTCATATGATAAATACCCCTTCCCCATT | Amplification promoter CipB for construction of CipB pMR6 |
| PCipB_MR7_R | CTTCACCTTTACTAACTGTATTCATATGATAAATACCCCTTCCCCATT | Amplification promoter CipB for construction of CipB pMR7 |
| P1ΔcomC | CAGCTATCAGCTGCCCTGTTAAAAGTC | Deletion comC |
| P4ΔcomC | GCAGGATACCCGTTATAATATCGGCCATC | Deletion comC |
| P1ΔcomD | TGCTAATGCTGTTAATCAGTCG | Deletion comD |
| P4ΔcomD | ATCTGAACAAGCAGGGGAGA | Deletion comD |
| P1ΔcomE | ATTTGCAGCAGGTGCCTTAG | Deletion comE |
| P4ΔcomE | CCTTGACAGCATCGTTTTGA | Deletion comE |
| P1ΔcomRS | CCGTCAGTTGGAGAAGGACT | Deletion comRS |
| P2ΔcomRS | \| GGCGCGCCTCTTTGGTCAGCCCCTTTTCT \| \| --- \| | Deletion comRS |
| P3ΔcomRS | GGCCGGCCTGTTTGCTAACACGGCACAG | Deletion comRS |
| P4ΔcomRS | ATTGGCAAAGATGACGGTGT | Deletion comRS |
| P1ΔcomS | TTATGCTTCCTTTTTGAAACTCGCT | Deletion comS |
| P4ΔcomS | CGCTATGTGAATTTGAAAAA | Deletion comS |
| P1ΔcomX | GGTGTAAAATCCGCCTGAAA | Deletion comX |
| P4ΔcomX | AAAAACCGCAAATCATGACG | Deletion comX |
| pIB166ComE_F | TAGAAGATTAAAGGAGAAATAATGATTTCTATT | Overexpression comE |
| pIB166ComE_R | TAAGGCTTCATTCATTTTGCTCTCCTTTGATC | Overexpression comE |
| pIB166ComS_F | GATATCAAAAAGAAAGGAGAATAACAGGATGTTTTCA | Overexpression ComS |
| pIB166ComS_R | CTTCGTTGATTCTATCTTGAGTCAAATGTGATATGTC | Overexpression comS |
| pIB166ComR_F | TAGACAGAGATTATAGGAAAAGGTTTTATGTTAAAAG | Overexpression comR |
| pIB166ComR_R | CATTATAAATGATCCTTCAAAAAAAGAACAGGACA | Overexpression comR |
| pIB166ComRS_F | TAGACAGAGATTATAGGAAAAGGTTTTATGTTAAAAG | Overexpression comR+S |
| pIB166ComRS_R | GATATGTCTATTGAATTTGCTTCTCTTTTTATTACTG | Overexpression comR+S |
| UP1342_F | TGATGTTACTTCAATGGATATTTTTGTTAGAG | Overexpression ComD; Amplification Upstream fragment *smu_1342* |
| UP1342_R | CAGTCTTAGGTCTGATTTTTTTAGTAATATTAAAAATCAAATTGTGCTATTTCTTTTG | Overexpression ComD; Amplification Upstream fragment *smu_1342* |
| P23_F | AATTTGATTTTTAATATTACTAAAAAAATCAGACCTAAGACTGATGACAAAAAGAGAA | Overexpression ComD; Amplification P23 promoter |
| P23_R | TATTACGATGACCTCCTTTTATTCATATTTTTCATTATATTTGGCCTCCCTT | Overexpression ComD; Amplification P23 promoter |
| ComD_F | \| CCAAATATAATGAAAAATATGAGCTGATCAAAGGAGAGCAAAATGAATGAAGC \| \| --- \| \|  \| | Overexpression ComD; Amplification *comD* |
| ComD_R | \| TTGTTGAACTAATGGGTGCTTTAAGATAAAACTATCTCTTAGAGAATAGGCCTC \| \| --- \| | Overexpression ComD; Amplification *comD* |
| Cat_F | ATGGAACATGTGATTGTACCATAAAGCACCCATTAGTTCAACAAACGAAAATTGGA | Overexpression ComD; Amplification *cat* |
| Cat_R | CTGATTCAGTTTCAAATAAATCAAAACCTTCTTCAACTAACGGGGCAGGTTAGTGACA | Overexpression ComD; Amplification *cat* |
| D1342_F | CCCGTTAGTTGAAGAAGGTTTTGATTTATTTGAAACTGAATCAGCTAAAAGATATTGGA | Overexpression ComD; Amplification downstream fragment *smu_1342* |
| D1342_R | GGCGCGCCACGGAAATAATTTTTAGTGACT | Overexpression ComD; Amplification downstream fragment *smu_1342* |
| PcomE_F1 | GACAGTGATTGCAGCAGGAGCTGGCT | Phosphorylmimetic Study |
| D60E_R1 | CTTTTCCTCTTTTTTGATTTCAATCTCCAAAAAGAAAATCTGGTGATTGCCCTTTTCAGG | Phosphorylmimetic Study |
| D60E_F2 | GCAATCACCAGATTTTCTTTTTGGAGATTGAAATCAAAAAAGAGGAAAAGAAAGGACTG | Phosphorylmimetic Study |
| comE_R2 | TCATTTTGCTCTCCTTTGATCAGCAATCACAGC | Phosphorylmimetic Study |
| D60A_R1 | CTTTTCCTCTTTTTTGATTTCAATAGCCAAAAAGAAAATCTGGTGATTGCCCTTTTCAGG | Phosphorylmimetic Study |
| D60A_F2 | GCAATCACCAGATTTTCTTTTTGGCTATTGAAATCAAAAAAGAGGAAAAGAAAGGACTG | Phosphorylmimetic Study |
| PCipB_MR4_R | CTTCTCCCTTTGAAACTGTATTCATATGATAAATACCCCTTCCCCAT | Amplification promoter CipB for construction of CipB pMR4 |
| PCipB_MR3_R | CCTCGCCCTTGCTCACTGTATTCATATGATAAATACCCCTTCCCCAT | Amplification promoter CipB for construction of CipB pMR3 |
| PCipB_MR5_R | CTCGCCCTTGCTCACTTCATTATCCATTACGTTAAATTGTTC | Amplification promoter CipB for construction of CipB pMR5 |
| PCipB_MR6_R | CCTTAATCAATTCTGATGTATTCATATGATAAATACCCCTTCCCCATT | Amplification promoter CipB for construction of CipB pMR6 |
| PCipB_MR7_R | CTTCACCTTTACTAACTGTATTCATATGATAAATACCCCTTCCCCATT | Amplification promoter CipB for construction of CipB pMR7 |
