# Supplementary material for: The Alternative Sigma Factor SigX Controls Bacteriocin Synthesis and Competence, the Two Quorum Sensing Regulated Traits in Streptococcus mutans
Source: PLoS Genet. 2015 Jul 9;11(7):e1005353. doi: 10.1371/journal.pgen.1005353 (PMC4497675; doi:10.1371/journal.pgen.1005353)
Supplement: S4 Table — The fluorescent reporter strains carrying the CipB pMR1 plasmid in the chromosome were constructed in backgrounds of ΔcomC, ΔcomD, ΔcomE, ΔcomS and ΔcomRS deletion strains, cultivated in CDM medium and induced by CSP (2 μM). Fluorescence microscopic images were obtained 3 hours after induction. Uninduced strains were used as controls. Tickmarks indicate detected fluorescence, tickmarks in brackets indicate weak fluorescence, a minus indicates no detectable fluorescence. (DOCX) [file pgen.1005353.s024.docx]

**Table S4. Expression of *comE* in different gene deletion background in CDM under CSP induced conditions.** The fluorescent reporter strains carrying the CipB pMR1 plasmid in the chromosome were constructed in backgrounds of Δ*comC*, Δ*comD*, Δ*comE*, Δ*comS* and Δ*comRS* deletion strains, cultivated in CDM medium and induced by CSP (2 µM). Fluorescence microscopic images were obtained 3 hours after induction. Uninduced strains were used as controls. Tickmarks indicate detected fluorescence, tickmarks in brackets indicate weak fluorescence, a minus indicates no detectable fluorescence.

| **CDM** | | | |
| --- | --- | --- | --- |
| **Reporter genotype** | **2 µM CSP** | **Control** | **strain** |
| comE | **(🗸)** | **-** | ComE pMR1 |
| comE ∆comC | **(🗸)** | **-** | ComE pMR1ΔcomC |
| comE ∆comD | **-** | **-** | ComE pMR1ΔcomD |
| comE ∆comE | **-** | **(🗸)** | ComE pMR1ΔcomE |
| comE ∆comS | **-** | **-** | ComE pMR1ΔcomS |
| comE ∆comRS | **-** | **-** | ComEpMR1ΔcomRS |
| comE ∆SigX | **-** | **-** | ComE pMR1ΔcomX |
