# Supplementary material for: The Alternative Sigma Factor SigX Controls Bacteriocin Synthesis and Competence, the Two Quorum Sensing Regulated Traits in Streptococcus mutans
Source: PLoS Genet. 2015 Jul 9;11(7):e1005353. doi: 10.1371/journal.pgen.1005353 (PMC4497675; doi:10.1371/journal.pgen.1005353)
Supplement: S5 Table — ComE pMR1 reporter strains with different gene deletion backgrounds were grown in THBY medium under CSP induced (2 μM) conditions. Additional deteails as in Table S4. (DOCX) [file pgen.1005353.s025.docx]

**Table S5. Fluorescence microscopic analysis of CSP induced *comE* fluorescent reporter strains in THBY.** ComE pMR1 reporter strains with different gene deletion backgrounds were grown in THBY medium under CSP induced (2 µM) conditions. Additional deteails as in Table S4.

| **THBY** | | | |
| --- | --- | --- | --- |
| **Reporter genotype** | **2 µM CSP** | **Control** | **strain** |
| comE | **🗸** | **-** | ComE pMR1 |
| comE ∆comC | **🗸** | **-** | ComE pMR1ΔcomC |
| comE ∆comD | **-** | **-** | ComE pMR1ΔcomD |
| comE ∆comE | **-** | **-** | ComE pMR1ΔcomE |
| comE ∆comS | **-** | **-** | ComE pMR1ΔcomS |
| comE ∆comRS | **-** | **-** | ComEpMR1ΔcomRS |
| comE ∆SigX | **-** | **-** | ComE pMR1ΔcomX |
