# Supplementary material for: The Alternative Sigma Factor SigX Controls Bacteriocin Synthesis and Competence, the Two Quorum Sensing Regulated Traits in Streptococcus mutans
Source: PLoS Genet. 2015 Jul 9;11(7):e1005353. doi: 10.1371/journal.pgen.1005353 (PMC4497675; doi:10.1371/journal.pgen.1005353)
Supplement: S6 Table — CipB pMR1 reporter strains with different gene deletion backgrounds were grown in THBY medium under CSP induced (2 μM) conditions. Details as in Table S4. (DOCX) [file pgen.1005353.s026.docx]

**Table S6. Fluorescence microscopic analysis of CSP induced *cipB* fluorescent reporter strains in THBY.** CipB pMR1 reporter strains with different gene deletion backgrounds were grown in THBY medium under CSP induced (2 µM) conditions. Details as in Table S4.

| **THBY** | | | |
| --- | --- | --- | --- |
| **Reporter genotype** | **2 µM CSP** | **Control** | **strain** |
| CipB | **🗸** | **-** | CipB pMR1 |
| CipB ∆comC | **🗸** | **-** | CipB pMR1ΔcomC |
| CipB ∆comD | **-** | **-** | CipB pMR1ΔcomD |
| CipB ∆comE | **-** | **-** | CipB pMR1ΔCipB |
| CipB ∆comS | **🗸** | **-** | CipB pMR1ΔcomS |
| CipB ∆comRS | **🗸** | **-** | CipB pMR1ΔcomRS |
| CipB ∆SigX | **🗸** | **-** | CipB pMR1ΔcomX |
