# Supplementary material for: The Alternative Sigma Factor SigX Controls Bacteriocin Synthesis and Competence, the Two Quorum Sensing Regulated Traits in Streptococcus mutans
Source: PLoS Genet. 2015 Jul 9;11(7):e1005353. doi: 10.1371/journal.pgen.1005353 (PMC4497675; doi:10.1371/journal.pgen.1005353)
Supplement: S1 Text — (DOCX) [file pgen.1005353.s027.docx]

**Text S1**

**Materials and Methods**

**Fluorescence microscopy**

*S. mutans* cultures grown in THBY were centrifuged (7000 rpm; 5 min) and washed two times with 0.85% of NaCl. The bacterial pellet was resuspended in 50-100 µl of NaCl. 3 µl of the re-suspended cells were transferred to a microscope slide and covered with a cover slide. Bacterial cells grown in CDM were directly transferred to a microscopic slide. Fluorescence microscopy was conducted using an Evos FL (Life Technologies, Germany) inverted microscope equipped with the Evos light cubes for TagBFP, GFP, RFP and Texas Red (Life Technologies, Germany)) and with a colorview camera (high-sensitivity monochrome, 1360 x 1024, 6,25 µm/pixel; (SONY® ICX 285 ACL CCD)). For microscopy of *S. mutans* a 100x magnification oil immersion objective was used. Overlay images were generated using the Cell Sense Standard Software (Olympus, Germany).

**RNA isolation**

Pelleted bacteria were washed with 500 µl nuclease free water and centrifuged for 2 min at 13.000 rpm. The supernatant was removed and the pellet resuspended in

LM-lysis buffer containing 2.5 mg lysozyme and 100 U mutanolysin (pH=7.4). The resuspension was shaken in a heating block at 25°C and 1400 rpm for 45 min. 700 µl of Qiazol Lysis solution (Qiagen, Germany) was added and the mixture transferred to a falcon tube containing sterile 0.1 µm glass beads (Carl Roth, Germany). The falcon tubes were vortexed for 3 min and centrifuged at 6000 rpm at RT to pellet the glass beads. The supernatant was transferred to a new tube and 200 µl of chloroform was added. Samples were vortexed for 15 sec, incubated for 2 min at RT and centrifuged for 20 min at 13.000 rpm and 4°C. The upper aqueous phase (approximately 550 µl) was transferred to a new tube and 870 µl of ethanol was added and mixed thoroughly. RNA extraction was carried out using the MiRNeasy Kit (Qiagen, Germany) according to the manufacturer’s instructions. To remove genomic DNA the optional on-column DNAseI digestion using the DNAse I Kit (Qiagen, Germany) was performed for 45 min. After the washing steps the RNA was eluted in 50 µl of nuclease free water supplied with the kit. To test the integrity of the isolated total RNA and the enriched mRNA, samples were analyzed using the Agilent 2100 Bioanalyzer and the RNA 6000 Pico Kit (Agilent, Germany).

**Transcriptome analysis of XIP and CSP induction in CDM**

Overnight cultures of the *S. mutans* UA 159 wildtype grown in THBY were diluted to an OD of 0.1 in CDM as described above and cultivated at 37°C and 5% CO_2_ until the culture had reached an OD of 0.175. Subsequently the culture was divided into three equal parts, one part was treated with 2 µM synthetic XIP, one with 2 µM synthetic CSP and the third part was used as an uninduced control. Samples were taken at 5, 15, 30, 60 and 120 min post addition of the synthetic peptides. The samples were immediately transferred into an equal volume of RNA Protect and incubated for 5 min at RT. Subsequently the bacteria were pelleted (13000 rpm, 2 min, RT), the supernatant was removed and the pellet was frozen at -70°C.

**mRNA enrichment and sequencing**

mRNA enrichment was performed using the RiboZero Kit (Epicentre, Illumina, Germany) and 2 µg of total RNA as described in the user`s instructions. Removal of 16S and 23S rRNA was controlled using the Bioanalyzer 2100 (Agilent, Germany). Direct strand-specific RNA sequencing was performed using the Illumina HiSeq 2000 platform (Illumina, Germany) according to the ScriptSeq (Agilent, Germany) protocol. Ten samples were multiplexed in one lane, yielding approx. 20 million 50 bp single end reads per sample. After quality control and clipping of the primers and barcodes data analysis was conducted using the Rockhopper software [58].

**Quantitative RT-PCR.**

Primers for quantitative PCR were designed using the Primer 3 software (http://frodo.wi.mit.edu) to generate amplicons in the range of 100 to 150 bp. All primers were purchased from MWG Eurofins Operon (Ebersberg, Germany). 1 µg of total RNA was reverse transcribed using the Quanti Tect Reverse Transcription Kit (Qiagen, Hilden, Germany) according to the manufacturer`s instructions. The cDNA was subsequently diluted 1:20 for use in PCR-reactions. For the amplification the QuantiTect Sybr Green Kit (Qiagen, Hilden, Germany) was used. 15 µl reactions with primer concentrations of 0.25 µM were run in the Light Cycler 480 (Roche, Germany). To determine the primer efficiencies serial dilutions of pooled cDNA were measured in triplicate. Each sample was measured in triplicate and each experiment was performed at least three times. For data normalisation a RNA-spike (specific for the red fluorescent protein (DsRed)) and the housekeeping gene SMU.1331 was used. SMU.1331 was found in a previously conducted microarray analysis to be not differentially expressed under the experimental conditions [59]. Data analysis was performed according to [60].

**Construction of single fluorescence reporter strains**

Fluorescent reporter strains were constructed on the basis of non-replicative plasmid pAE03 encoding the promoterless GFP+ gene and an erythromycin resistance cassette [61]. The plasmid sequence was amplified with fusion polymerase using primers P1AE03 and P2AE03 (Table S3). Promoter sequences of analysed genes were amplified using the specific primers listed in table S3 comprising a 15 bp 5’ flank which is homologous to the plasmid sequence. Homologous flanks allow seamless insertion of promoter sequences into pAE03 upstream of the promoterless GFP+ genes in a recombinase mediated approach (CloneEZ, Genscript). Plasmids were transformed in *Escherichia coli* DH5α and the correct plasmid sequence was verified by sequencing. Transformation of the resulting reporter plasmids into *S. mutans* was accomplished according to the procedure described by Li *et al.* [62]. The entire plasmid sequences were integrated into the *S. mutans* chromosome via single homologous recombination at the native promoter loci. Antibiotic resistant clones were selected on THBY agar plates containing the appropriate antibiotic. Colonies were picked and glycerol stocks containing 15% (v/v) glycerol in fresh THBY including appropriate antibiotics were prepared.

**Construction of dual fluorescence reporter strains**

For the construction of dual reporter strains the erythromycin resistance cassette of pAE03 was replaced with the tetracycline resistance cassette of plasmid pJWV25 [61]. For the amplification of the plasmid backbone primers P3AE03 and P4AE03 were used, while primers TetM_F and TetM_R were used to amplify the resistance cassette. The above described recombinase mediated cloning approach was performed to exchange the two resistance cassettes. The resulting plasmid pMR1 (figure S1) was used to construct reporter strains as described for pAE03 above.

For the exchange of the GFP+ gene against the TagBFP2 gene primers P5AE03 and P6AE03 were used to amplify the plasmid sequence. An *S. mutans* codon-optimized TagBFP2 gene was designed utilizing the Genious software (MWG Eurofins Operon, Germany) and the gene was subsequently synthesized by MWG Eurofins Operon. Amplification of the gene sequence for cloning was conducted with the Primers TagBFP2_F and TagBFP2_R and the PCR product was subsequently inserted into the GFP+ excised pAE03 vector with the same recombinase-mediated procedure as described above. The resulting plasmid pMR2 (figure S1) functioned as basis for the construction of dual fluorescent reporter strains. Table S1 lists all constructed dual fluorescent reporter strains and table S3contains all primers used for the construction of these strains. Dual reporter strains were selected on THBY plates containing 10 µg/ml erythromycin and 12.5 µg/ml tetracycline.

**Construction of the LytFsm pMR1 LytFsm pMR2 reporter strains**

Theis reporter differs from all other constructs since two plasmids were integrated into the same locus. The two integration steps are schematically shown in Fig. S3. After integration of the first plasmid into the PLytFsm locus via single homologous recombination 3 different homologous sequences are present in the genome which would allow integration of the second plasmid. First the PLytFsm locus 5` of GFP+, second the PlytFsm sequence 5`of the native LytFsm gene and third parts of the plasmid sequence. However single homologous recombination into any of these loci results in a genomic context where TagBFP2 and GFP+ are individually under the control of a LytFsm promoter. Selection with double antibiotics was perfomed to select for double integrants.

For all other dual reporter strains integration of the two different plasmids occurs into two different genomic loci. Correct integration was always checked via PCR.

**Construction of fluorescent reporter strains in different gene deletion backgrounds**

Reporter strains based on the pMR1 plasmid were transformed with 100 ng of a PCR product amplified from genomic DNA of previously constructed gene deletion mutants using primers flanking the entire knocked-out region (P1/P4). Gene deletion mutants used as donor strains for gDNA were constructed according to the procedure of Lau *et al.* as previously reported [63]. Table S3 listed the primers used for the construction of the gene deletion mutants. Transformed strains were plated on THBY agar containing 10 µg/ml erythromycin and 12.5 µg/ml tetracycline.

**Results**

**Transcriptional fluorescence reporter strain system for *S. mutans***

To monitor the expression of genes along the entire CSP-induced competence cascade of *S. mutans* on the single cell level, we constructed a toolbox of chromosomal fluorescent reporter strains. Reporters for the genes encoding the response regulator ComE, the XIP-precusor ComS, and the master regulator of competence SigX were established. Additionally reporters for various late competence genes (*lytFsm*, *smu_498*, *smu_625*, *smu_644,* *smu_1001*, *smu_1987*), bacteriocin encoding genes (*cipB*, *mutacin IV* and *VI*) and genes encoding immunity proteins (*cipI*, *smu_1913*) were constructed. All constructs were based on the nonreplicative plasmid pAE03 which allows chromosomal integration of the entire plasmid at the native locus of the promoter region via single homologous recombination [61]. Moreover we replaced the erythromycin resistance cassette of the plasmid pAE03 with the tetracycline resistance cassette *tetM* of plasmid pJWV25 [61], resulting in plasmid pMR1 (Figure S1). Based on this plasmid we constructed chromosomal reporter strains in different gene deletion backgrounds. As previously constructed gene deletion mutants used the erythromycin resistance cassette as selective marker [63]. The TetM gene was chosen as an additional resistance cassette for construction of dual reporter strains. Fluorescent reporter strains described above were constructed in the *ΔcomE*, *ΔcomS*, *ΔcomRS*, *ΔsigX*, *ΔcomD* and *ΔcomC* background. Table S1 shows all reporter strains constructed for this study.

**Improvement and validation of the *cipB* reporter strain**

Lemme *et al.* had previously reported that the fluorescence of their *cipB* reporter strain was low although qRT-PCR results indicated strong expression of the gene [14]. This discrepancy was most likely causedby inefficient translation of the *cipB*-GFP mRNA. Therefore we constructed a new *cipB* reporter plasmid (CipB pAE03) allowing chromosomal integration of the construct at the native locus. Moreover a seamless recombinase mediated cloning approach (Clone EZ; Genscript, USA) was used which does not add restriction sites into the 5`UTR region of the GFP gene, altering the native 5` UTR of *cipB*. In the constructs of Lemme *et al.* an additional ATC was located in the 5` UTR region. The distance between the ribosomal binding site and the start codon significantly influences the translational efficiency of mRNA [64] and therefore these additional three basepairs might provide an explanation for the weak GFP fluorescence of this construct. To further improve translation efficiency, additionally in our constructs the first three codons of the *cipB* gene were placed at the 5` end of the GFP coding sequence to mimic the translation start of the gene under study. Finally the codon-usage of the GFP-gene used here was optimized for bacteria with a low GC content to ensure efficient translation by *S. mutans* [36,61]. Consequently and in contrast to the findings of Lemme *et al.* the CSP-induced GFP expression from the *cipB* promoter was the highest amongst all reporter constructs studied here, in accordance with the results of the transcriptional analysis from Perry *et al.* [34], Lemme *et al.* [14] and own findings. The data show that the cipB pAE03 reporter strain reliably reflects expression of *cipB* and highlight the importance of a careful examination of all factors that might influence the translation efficiency of fluorescent proteins in the reporter strains.

**Dual fluorescent reporter strains for *S. mutans***

To simultaneously analyze the promoter activity of two different genes involved in competence development in *S. mutans* on the single cell level, we constructed dual fluorescent reporter strains. First we combined the previously established pMR1 based (GFP+) reporter strains with reporter constructs which were based on a second fluorescent protein with a longer emission wavelength in the orange to red range (mCherry, TurboRFP, tdTomato). However all those reporter strains showed a significantly delayed development of fluorescence compared to their GFP counterparts (figure S2). Codon optimization of these proteins for *S. mutans* and application of a folding Tag [65] did not improve the slow maturation of the fluorophores (data not shown). Thus we then utilized a fluorophore with a shorter emission wavelength than GFP for construction of dual reporter strains. TagBFP2 represents the brightest and most stable fluorophore emitting photons in the blue wavelength [66]. We utilized a *S. mutans* codon-optimized gene coding for TagBFP2 to construct the non-replicative reporter plasmid pMR2 (figure S1). In combination with constructs based on plasmid pMR1 dual fluorescent reporter strains were realized in *S. mutans*.

To validate the functionality of the dual fluorescence reporter strains and to prove the validity of the conclusion made from their analysis, we constructed a reporter strain (LytFsm pMR1 LytFsm pMR2) carrying the identical promoter region of *lytFsm*) 5` from both the promoterless GFP+ and the TagBFP2 encoding gene in the chromosome (table S1, figure S3). The stepwise genomic integration of the plasmids used for the generation of this particular reporter strain and the resulting genomic context is schematically shown in figure S3. *LytFsm* is a late competence gene controlled by SigX and showed the highest expression of all tested late competence genes (figure S4). Microscopic analysis revealed that the same cells expressing GFP also express TagBFP2 and vice versa (figure S5 A). Thus we conclude that the established dual reporter strain system is an appropriate tool to analyze coexpression and population heterogeneity in *S. mutans*. However, as reported before [66,67] we observed a considerably higher background fluorescence in the blue channel due the higher energy of the photons used for excitation of TagBFP2. The spectral properties of TagBFP2 and GFP+ allow a clear discrimination between both signals (figure S6), no spectral crosstalk was observed.

**CSP induced transcription of *comE* and *cipB***

Using the ComE pMR1 and CipB pMR1 reporter strains in the different deletion backgrounds we also tested whether CSP induction in chemically defined medium (CDM) promotes transcriptional activation of *comE* and expression of bacteriocins. Under these conditions competence is not induced by synthetic CSP, regardless of the used concentration [15]. It was speculated that CSP might be proteolytically degraded by the HtrA protease in CDM, while in complex medium this protease was thought to be saturated by small peptides that are constituents of the medium, thus protecting CSP from degradation [15]. However here we show (table1 and figure S13) that CSP promotes a strong induction of *cipB* expression in CDM and thus is present and biologically active. Induction of *cipB* expression occurs via ComDE, as deletion of either *comD* or *comE* completely abolished it. An influence of other regulatory systems apart from *comDE* can thus be excluded. ComE expression is only weakly induced by CSP addition (table 1 and figure S16) to CDM medium and its transcriptional activation is significantly delayed in comparison to the induction of its target *cipB* (figure S14). At timepoints (30-120 min) where *cipB* is highly induced, comE is not differentially expressed.

The finding that *comE* is weakly induced in CDM under CSP induced conditions for later timepoints after induction (180 min) is surprising since SigX should not be activated under these conditions and thus should not bind to *comE*. In the transcriptome analysis it was shown that comE is not transcriptionally regulated upon CSP induction in CDM (figure 5). Density dependent endogenous production of XIP by the growing culture (WT background) could be responsible for this observation. The endogenously produced XIP activates SigX and thus *comE* expression. Furthermore this would be in accordance with the finding that transcriptional activation of *comE* was abolished in the Δ*comS* and Δ*sigX* background. In these strains either internal production of XIP is abolished (Δ*comS*) or deletion of SigX prevents transcriptional activation of *comE*. We demonstrate accumulation of endogenously produced XIP by the growing culture in CDM in figure S18. A description of the conducted experiment can be found below.

We showed (figure 8) that the mechanism triggering induction of *comE* expression must be different from the mechanisms responsible for the induction of *cipB* expression. In CDM under CSP induced conditions we observed that *cipB* is transcriptionally activated fast while at corresponding timepoints the expression of its direct regulator ComE is not enhanced at all (figure S14). Thus both results indicate that ComE, while being expressed at a basal level, might be activated post-transcriptionally by CSP addition. In turn it activates transcription of its targets *cipB*. As ComE is a response regulator of a TCS we assume that most likely phosphorylation activates ComE to induce bacteriocin expression. We tested this hypothesis using phosphomimetic mutants (figure 9). The transcriptional induction of *comE* observed in THBY (see above) occurs delayed, via SigX binding to the *comE* promoter, in a distinct second mechanism. However in CDM under CSP induced conditions SigX is not activated, accordingly the transcriptional level of ComE is not enhanced.

**Accumulation of endogenously produced autoinducers in the supernatant upon growth in CDM**

Above we demonstrated that externally added XIP induces bacteriocin expression upon growth of *S. mutans* in CDM. We were curious to know whether bacteriocin expression in CDM also occurs upon growth of the culture by the accumulation of endogenously produced XIP or CSP. Thus *cipB* and *comE* reporter strains in different gene deletion backgrouds were cultivated in CDM and their fluorescence was analyzed after the cultures reached the stationary phase (8h growth). Fluorescence overlay images of the different strains are shown in figure S18. In the wildtype background a strong induction of the CipB reporter strain was observed (upper panel). This transcriptional activation of CipB is dependent on *comS* but not on *comC*. Thus we can conclude that XIP accumulates in the supernatant and not CSP. XIP induces bacteriocin expression via SigX, accordingly no CipB induction is observed in the *sigX* deletion background. In total agreement with the findings of the CipB reporter strain we observed dependency of *comE* expression on *sigX* and *comS* but not on *comC* and *comD* (lower panel).

**Bacteriocin Overlay Assay**

We tested bacteriocin production of the S. mutans WT strain and strains lacking the genes encoding the XIP and CSP autoinducers (Δ*comC* and Δ*comS*) using a bacteriocin overlay assay on CDM agar. As positive control these strains were tested on THBY agar with and without CSP induction and thus under conditions that allow bacteriocin production as reported previously. *L. lactis* and *S. sanguis* were utilized as indicator strains for mutacins CipB and NlmAB (MutacinV and IV, respectively). Surprisingly we did not find inhibitory activity of either XIP induced or uninduced strains on CDM agar (figure S17 B). For CSP induced WT strains growing on CDM agar also no inhibitory effect on the indicator strains was observed. In contrast, growth of the uninduced WT and the Δ*comS* deletion strain on THBY agar is accompanied by the secretion of bacteriocins, as indicated by the occurrence of inhibitory zones for *L. lactis* and *S. sanguis* (figure S17 A). Induction of these strains with CSP further increases the inhibitory zones suggesting that more bacteriocin is produced. For the uninduced Δ*comC* deletion strain no inhibitory effect on the indicator strains was observed upon growth on THBY agar. This finding is in accordance with the loss of capacity to endogenously produce CSP and self-induce bacteriocin expression. Addition of external CSP restored the phenotype and accordingly inhibitory zones were observed on the THBY agar plate for the CSP induced strain. Thus we can conclude that bacteriocins were not expressed upon growth on CDM agar, regardless if CSP or XIP were used for induction.

Moreover we tested the concentrated supernatants (ultrafiltration, precipitation with acetone, ammoniumsulfate, chloroform) of planktonic growing cultures in CDM post XIP and CSP supplement. Again we did not find inhibitory activity against the two indicator strain using a spot assay. Finally we tested the influence of the pH on bacteriocin production. As *S. mutans* is highly acidogenic and the CDM medium is strongly buffered (75 mM phosphate buffer) we utilized unbuffered CDM for the overlay assay. However inhibitory activity on the indicator was not detected. Thus we conclude that the bacteriocin expression is regulated posttranscriptionally upon growth in CDM. These findings are furthermore in accordance with the studies of Dawid *et al*. and Kochan *et al*. working on the orthologous Blp bacteriocin system in *S. pneumoniae*. The authors showed that the HtrA protease regulates bacteriocin activity posttranscriptionally and blocks secretion and processing of the pheromone BlpC in *S. pneumoniae*. Accordingly we found that CSP is either not produced or secreted in CDM (figure S18). Strikingly bacteriocins and CSP were exported and processed via the same ABC transporter (ComAB).

**SigX controls expression of *comE***

Table S4 shows that in complex medium *comE* is induced by CSP in the wildtype and in the Δ*comC* background. The latter is not surprising since *comC* encodes the precursor of CSP and has no role in the signal detection or transduction. As expectd, deletion of either *comD* or *comE* blocks induction of *comE* by CSP. *ComD* encodes the histidine kinase and *comE* the response regulator of the TCS which senses CSP (Fig. 1). However, deletion of *comRS* and surprisingly also *sigX* completely abolished transcriptional activation of *comE*. Thus *sigX* or one of its downstream targets, but not *comR* or *comS*, must be responsible for inducing *comE* transcription. A regulatory role of the alternative sigma-factor for the transcription of *comE* is demonstrated by this result.

**Quantitative PCR**

As an independent method to confirm the flow cytometric data we used quantitative reverse transcription PCR (qRT-PCR) (Fig. S15). *cipB* expression was already strongly (27 fold) induced 5 minutes post CSP supplementation and increased to 100 fold relative expression 15 minutes after induction. XIP supplementation did not induce *cipB* expression at all for these timepoints. By constrast, *comE* expression was only very slightly induced by the addition of CSP (1.5 fold after 5 min and 1.7 fold 15 minutes post supplementation of the CSP peptide). This shows that transcription of *comE* plays only a minor role for inducing *cipB* expression.

Interestingly, using XIP as inducer, *comE* was not differentially expressed 5 minutes after induction but significantly induced (3 fold) 15 minutes post XIP supplementation. At that timepoint its target *cipB* shows absolutely no differential expression. The temporal delay of *comE* expression is in accordance with the suggested mechanism of SigX binding to the promoter region of *comE*.

**Discussion**

The presently available toolbox of bright fluorescent proteins with distinct excitation and emission spectra opens the door to various multicolor *in-vivo* labelling approaches in the cell [68]. Reporter systems based on fluorescent proteins have been proven to be indispensable for the analysis of population heterogeneity and single cell analysis of various phenotypes [43,69,70]. Here we utilized for the first time, to the best of our knowledge, a dual fluorescent reporter system to analyze the signal propagation along a signaling cascade in a bacterium on the single cell-level.

*S. mutans* has evolved a complex regulatory system to simultaneously control competence and bacteriocin expression in a cell density dependent manner. As demonstrated by our results, the two QS circuits controlling the expression of the involved machineries are intimately linked. Moreover population heterogeneity is observed during competence development and regulation occurs at the transcriptional level as well as post-translationally by phosphorylation. Such complex regulatory systems cannot be understood by studying the expression of single genes in isolation or by global approaches measuring gene expression as an average of all cells in the population. Dual or multi-color single cell transcriptional reporter strains represent novel tools capable of unraveling such signaling cascades. They can be applied to determine the origin of heterogeneity and to gain insights into additional regulatory inputs and connections between different parts of the regulons. With the development of bright and stable fluorescent proteins in the near and the far range of the visible spectrum it should theoretically be possible to simultaneously monitor the transcription of 4 genes in parallel [67,68]. However, several drawbacks have to be solved until these reporter systems can be successfully applied in bacteria.

Monitoring signaling in the cell is often hampered by the relatively slow maturation of the fluorophores and a lower sensitivity when compared to conventional enzyme-based reporter systems [68]. Thus very few reports about the utilization of dual transcriptional reporters are found in the literature. By contrast, studying the expression of two genes in parallel is often accomplished by the combination of a fluorescent reporter with e.g. a bioluminescence based reporter [71]. Fluorescent transcriptional reporter strains constitutively expressing a second fluorescent protein have been used for normalization to subtract the effects of intrinsic noise on gene expression and thus enhance the accuracy in single cell transcriptional analyses [72]. More recently an attenuation based dual fluorescent protein reporter was constructed for the screening of translation inhibitors [73]. Kojima *et al*. [74] developed a dual fluorescence reporter assay to study gene silencing of a small hairpin RNA at the single cell level. In *V. cholera* the co-localization of a so called pseudopilus, which is part of the DNA uptake machinery, with the outer membrane secretin PilQ was demonstrated using a dual color approach [6]. Transcriptional fluorescent reporter strains in streptococci were until now limited to single color applications [14,15,34]. However Beilharz *et al*. visualized co-localization of the serin/threonine protein kinase StkP with the cell division proteins DivIVa and FtsA using RFP and GFP fusion proteins in *S. pneumoniae* [75].

Fluorescent proteins show maturation times between 15 minutes up to several hours [67,68]. Competence development in complex media in *S. mutans* is a slow process, the maximaum transformability of the bacterium being observed 3 hours after CSP stimulation [14,34]. Thus the application of fluorescence reporters for single cell analysis of competence development in *S. mutans* is feasible.

The successful application of dual fluorescent transcriptional reporter strains is complicated by differences in maturation times, spectral properties and stabilities of the fluorophores under different environmental conditions. Combinations of GFP+ with red fluorescent proteins were not successful in our approach since the maturation speed of the red fluorophore was considerably slower than that of GFP. Interestingly, this effect has already been utilized to construct tandem fluorescent proteins used as timers for an *in vivo* analysis of protein kinetics [76].

For mCherry and other DsRed derived fluorophores, sigmoidal maturation kinetics were found and maturation occurred in a three step process comprising two rate-limiting oxidation reactions [77]. In contrast, GFP maturates with a single oxidation step which can be described with an exponential curve [78]. Thus DsRed derived proteins need 2 molecules of molecular oxygen per fluorophore molecule for maturation and mature considerably slower when compared to GFP derivatives. The microaerophilic conditions present in the cultivation medium and the cytoplasm of streptococci thus limit the application of red fluorescent proteins in these organisms. Therefore mCherry and other DsRed derivatives do not represent appropriate reporter proteins for gene expression analysis in streptococci.

Consequently we utilized TagBFP2 for the construction of dual fluorescent reporter strains. TagBFP2 is clearly distinct from GFP in its spectral properties, and its brightness is comparable to most GFP variants [66]. Moreover as TagBFP2 is a derivative of the GFP fluorophore, its maturation time is in the same order of that observed for GFP. Thus TagBFP2 represents an excellent partner for GFP in dual color applications in streptococci.

Reference List

57. Heckman KL, Pease LR (2007) Gene splicing and mutagenesis by PCR-driven overlap extension. Nat Protoc 2: 924-932. nprot.2007.132 [pii];10.1038/nprot.2007.132 [doi].

58. McClure R, Balasubramanian D, Sun Y, Bobrovskyy M, Sumby P, Genco CA, Vanderpool CK, Tjaden B (2013) Computational analysis of bacterial RNA-Seq data. Nucleic Acids Res 41: e140. gkt444 [pii];10.1093/nar/gkt444 [doi].

59. Reck M, Rutz K, Kunze B, Tomasch J, Surapaneni SK, Schulz S, Wagner-Dobler I (2011) The biofilm inhibitor carolacton disturbs membrane integrity and cell division of Streptococcus mutans through the serine/threonine protein kinase PknB. J Bacteriol 193: 5692-5706. JB.05424-11 [pii];10.1128/JB.05424-11 [doi].

60. Pfaffl MW (2001) A new mathematical model for relative quantification in real-time RT-PCR. Nucleic Acids Res 29: e45.

61. Eberhardt A, Wu LJ, Errington J, Vollmer W, Veening JW (2009) Cellular localization of choline-utilization proteins in Streptococcus pneumoniae using novel fluorescent reporter systems. Mol Microbiol 74: 395-408. MMI6872 [pii];10.1111/j.1365-2958.2009.06872.x [doi].

62. Li YH, Tang N, Aspiras MB, Lau PC, Lee JH, Ellen RP, Cvitkovitch DG (2002) A quorum-sensing signaling system essential for genetic competence in Streptococcus mutans is involved in biofilm formation. J Bacteriol 184: 2699-2708.

63. Lau PC, Sung CK, Lee JH, Morrison DA, Cvitkovitch DG (2002) PCR ligation mutagenesis in transformable streptococci: application and efficiency. J Microbiol Methods 49: 193-205. S0167701201003694 [pii].

64. Chen H, Bjerknes M, Kumar R, Jay E (1994) Determination of the optimal aligned spacing between the Shine-Dalgarno sequence and the translation initiation codon of Escherichia coli mRNAs. Nucleic Acids Res 22: 4953-4957.

65. Henriques MX, Catalao MJ, Figueiredo J, Gomes JP, Filipe SR (2013) Construction of improved tools for protein localization studies in Streptococcus pneumoniae. PLoS One 8: e55049. 10.1371/journal.pone.0055049 [doi];PONE-D-12-32515 [pii].

66. Subach OM, Cranfill PJ, Davidson MW, Verkhusha VV (2011) An enhanced monomeric blue fluorescent protein with the high chemical stability of the chromophore. PLoS One 6: e28674. 10.1371/journal.pone.0028674 [doi];PONE-D-11-09521 [pii].

67. Shaner NC, Steinbach PA, Tsien RY (2005) A guide to choosing fluorescent proteins. Nat Methods 2: 905-909. nmeth819 [pii];10.1038/nmeth819 [doi].

68. Giepmans BN, Adams SR, Ellisman MH, Tsien RY (2006) The fluorescent toolbox for assessing protein location and function. Science 312: 217-224. 312/5771/217 [pii];10.1126/science.1124618 [doi].

69. Zaslaver A, Bren A, Ronen M, Itzkovitz S, Kikoin I, Shavit S, Liebermeister W, Surette MG, Alon U (2006) A comprehensive library of fluorescent transcriptional reporters for Escherichia coli. Nat Methods 3: 623-628. nmeth895 [pii];10.1038/nmeth895 [doi].

70. Maamar H, Dubnau D (2005) Bistability in the Bacillus subtilis K-state (competence) system requires a positive feedback loop. Mol Microbiol 56: 615-624. MMI4592 [pii];10.1111/j.1365-2958.2005.04592.x [doi].

71. Uliczka F, Pisano F, Kochut A, Opitz W, Herbst K, Stolz T, Dersch P (2011) Monitoring of gene expression in bacteria during infections using an adaptable set of bioluminescent, fluorescent and colorigenic fusion vectors. PLoS One 6: e20425. 10.1371/journal.pone.0020425 [doi];PONE-D-11-03036 [pii].

72. Figueira R, Watson KG, Holden DW, Helaine S (2013) Identification of salmonella pathogenicity island-2 type III secretion system effectors involved in intramacrophage replication of S. enterica serovar typhimurium: implications for rational vaccine design. MBio 4: e00065. mBio.00065-13 [pii];10.1128/mBio.00065-13 [doi].

73. Osterman IA, Prokhorova IV, Sysoev VO, Boykova YV, Efremenkova OV, Svetlov MS, Kolb VA, Bogdanov AA, Sergiev PV, Dontsova OA (2012) Attenuation-based dual-fluorescent-protein reporter for screening translation inhibitors. Antimicrob Agents Chemother 56: 1774-1783. AAC.05395-11 [pii];10.1128/AAC.05395-11 [doi].

74. Kojima S, Borisy GG (2014) An image-based, dual fluorescence reporter assay to evaluate the efficacy of shRNA for gene silencing at the single-cell level. F1000Res 3: 60. 10.12688/f1000research.3-60.v1 [doi].

75. Beilharz K, Novakova L, Fadda D, Branny P, Massidda O, Veening JW (2012) Control of cell division in Streptococcus pneumoniae by the conserved Ser/Thr protein kinase StkP. Proc Natl Acad Sci U S A 109: E905-E913. 1119172109 [pii];10.1073/pnas.1119172109 [doi].

76. Khmelinskii A, Keller PJ, Bartosik A, Meurer M, Barry JD, Mardin BR, Kaufmann A, Trautmann S, Wachsmuth M, Pereira G, Huber W, Schiebel E, Knop M (2012) Tandem fluorescent protein timers for in vivo analysis of protein dynamics. Nat Biotechnol 30: 708-714. nbt.2281 [pii];10.1038/nbt.2281 [doi].

77. Hebisch E, Knebel J, Landsberg J, Frey E, Leisner M (2013) High variation of fluorescence protein maturation times in closely related Escherichia coli strains. PLoS One 8: e75991. 10.1371/journal.pone.0075991 [doi];PONE-D-13-26277 [pii].

78. Heim R, Cubitt AB, Tsien RY (1995) Improved green fluorescence. Nature 373: 663-664. 10.1038/373663b0 [doi].
